# LC-MS/MS Strategy Using Substructure Searching for the Annotation of Cyanopeptide Classes: Implications for New Compound Discovery and Environmental Monitoring

**DOI:** 10.1021/acs.est.6c05607

**Published:** 2026-06-20

**Authors:** Runjie Xia, Lindsey Ahn, Michaela Burkhauser, Ross Youngs, Matthew J. Bertin

**Affiliations:** † Department of Chemistry, 2546 Case Western Reserve University, Cleveland, Ohio 44106, United States; ‡ Biosortia, Inc., 2545 Farmers Dr., Suite 370, Columbus, Ohio 43235, United States

**Keywords:** cyanoHABs, cyanopeptides, micropeptins, LC-MS/MS, protease inhibition

## Abstract

Cyanobacterial harmful algal blooms (cyanoHABs) are a major ecological and public health concern, commonly monitored for hepatotoxic microcystins and cylindrospermopsins and neurotoxic anatoxins and saxitoxins. However, the broader suite of bioactive metabolites produced during blooms remains undercharacterized. Here, we interrogated a chromatography fraction library generated from a cyanoHAB in Muskegon, Michigan. From this library, we isolated two new micropeptins (**1** and **2**), including an analog bearing a bishomologated tyrosine residue, and we confirmed the structure of ferintoic acid C (**3**). Structures were established using complementary spectrometric and spectroscopic methods. To expand chemical space coverage beyond isolated compounds, we analyzed liquid chromatography-tandem mass spectrometry (LC-MS/MS) data using the Global Natural Products Social Molecular Networking 2 (GNPS2) Analysis Hub query language for product ion searching, enabling annotation of cyanopeptide classes and class-specific modifications across the fraction set, which provided a practical and user-friendly strategy for identifying cyanopeptide classes. One of the new micropeptins (**1**) exhibited moderate inhibition of neutrophil elastase, consistent with roles in ecological interactions and potential relevance to human exposure. Analysis of field samples from ongoing Lake Erie blooms showed recurring micropeptins but no evidence of microcystins. Together, these results challenge microcystin-centric assessments of bloom hazard and support expanded monitoring of nonmicrocystin cyanopeptides.

## Graphical abstract

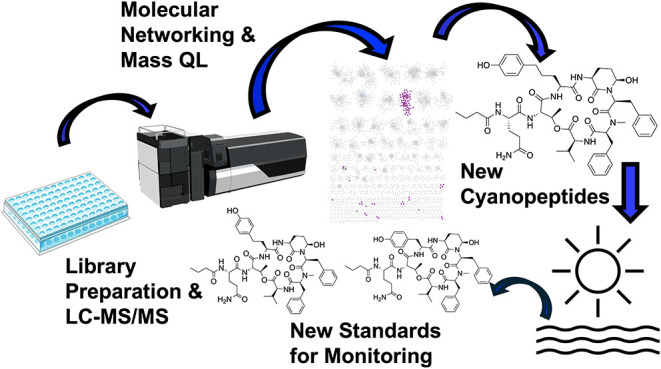

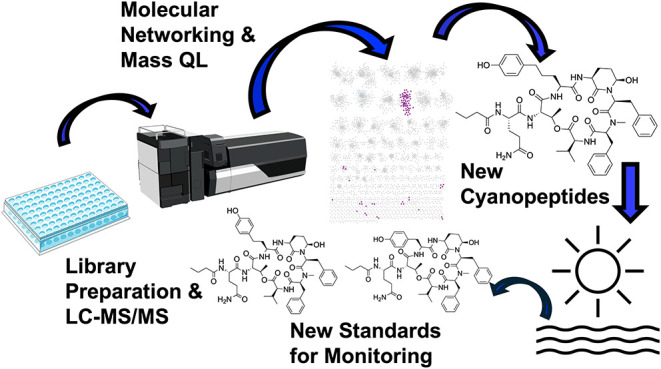

## Introduction

Cyanobacterial harmful algal blooms (cyanoHABs) are expanding globally and represent a persistent threat to water quality and human health through the production of toxic secondary metabolites.^1,2^ In most freshwater systems, monitoring and risk management are centered on microcystins, a structurally diverse family of hepatotoxins that is the basis for international guidance values and U.S. drinking-water health advisories.^3,4^ However, the current monitoring approach does not capture the broader metabolite mixtures produced during blooms, which is increasingly being shown in more and more studies.^5–7^

Cyanobacteria generate extensive suites of “cyanometabolites”, including numerous cyanopeptides assembled by nonribosomal peptide synthetases and other biosynthetic pathways, such as micropeptins/cyanopeptolins, anabaenopeptins, microviridins, microginins, and others in addition to the large class of microcystins. These compounds can exhibit potent bioactivities and may influence ecological interactions, food-web transfer, and human exposure profiles.^8^ A growing body of evidence indicates that nonmicrocystin cyanopeptides can occur as frequently as microcystins and sometimes at similar concentrations in surface waters, yet their environmental distributions, toxicity, and risk relevance remain less defined.^7–9^ Recent work has shown that anabaenopeptins/ferintoic acids and cyanopeptolins/micropeptins have similar abundance and temporal dynamics to that of microcystins,^10^ and time series tracking over five years showed that anabaenopeptins were detected in 95% of all samples, higher than that of microcystins (70% of all samples), while planktopeptin BL1125 (a cyanopeptolin/micropeptin congener) was observed in approximately 40% of all samples.^11^ Anabaenopeptins inhibit the same protein phosphatases as microcystins with many anabaenopeptin congeners showing nanomolar IC_50_ values in enzyme inhibition assays,^12^ and cyanopeptolin 1020 showed neurotoxic effects against zebrafish and toxicity to invertebrates.^13,14^ The imbalance in nonmicrocystin cyanopeptide analysis and monitoring reflects practical barriers: high structural diversity, limited availability of reference standards, and analytical complexity that challenges routine targeted monitoring. Consequently, there is a need for strategies that both enable structure-level characterization of newly observed congeners and expand class-level screening across complex bloom events.

High-resolution LC–MS/MS paired with open computational resources offers a scalable approach to address these challenges. GNPS provides widely used infrastructure for organizing and annotating untargeted MS/MS data, including molecular networking and repository-scale spectral interpretation.^15^ GNPS2 extends these capabilities through a web-based analysis hub and tools designed for accessible interrogation of MS/MS data sets including a newly available query language to assess a multitude of molecular features such as halogenation and product ion composition.^16,17^ Such platforms can facilitate class-level annotation via diagnostic fragments and product ion patterns, complementing targeted quantification and isolation-based structure elucidation.

Here, we apply an integrated discovery and annotation strategy to a cyanoHAB from Muskegon, Michigan. We interrogated a chromatography fraction library, enabling both targeted isolation for rigorous structural characterization and untargeted LC–MS/MS analysis for broader metabolite annotation. Using complementary spectrometric and spectroscopic methods, we report two new micropeptins (**1** and **2**) and confirm the structure of ferintoic acid C (**3**), a metabolite previously assigned via MS/MS spectrum interpretation.^18^ We then leveraged GNPS2 product ion searching to annotate cyanopeptide classes (e.g., microcystins, anabaenopeptins, microviridins, micropeptins, etc.) and modifications across the fraction set, which allowed for additional mass spectrometry based characterizations providing a practical screening approach that reduces reliance on reference standards for every congener. Finally, we assessed protease inhibitory activities and examined field relevance by surveying Lake Erie bloom samples to evaluate whether micropeptins represent a recurring component of Great Lakes cyanoHAB chemical profiles. This work ultimately provides an integrated strategy of mass spectrometry-driven class-level and new metabolite annotation in field samples and unequivocal identification of emerging cyanometabolites by chromatographic comparison to a standard library of rare cyanopeptides. Collectively, this work delivers a transferable and user-friendly LC–MS/MS annotation framework and reference library resources that can be applied to bloom surveillance to improve detection of emerging cyanopeptide hazards in source waters and recreational systems.

## Materials and Methods

### LC-MS/MS Analysis and Molecular Networking

We purchased a library of preparative chromatography fractions from Biosortia Microbiomics (20 fractions). The library was derived from a cyanobacterial bloom originally collected from Muskegon, MI in a holding lagoon at the Muskegon County Wastewater Management facility. Microscopic examination showed that the bloom was dominated by *Microcystis aeruginosa*.^9^ This library resulted from extraction and fractionation procedures that have been described previously,^9^ and 15 mg of MC-LR were isolated from 43 kg of the original lyophilized biomass.^9^ We have explored it previously using bioassay-guidance to characterize new microcystins and new micropeptins and other cyanopeptides.^5,19^ The individual fractions were reconstituted in LC-MS grade CH_3_OH and each fraction (10 μL injection volume) was subjected to LC-MS/MS analysis on an Agilent Revident QToF mass spectrometer with a 1290 Infinity II Bio LC and column for analytical separations. Analyte separation was performed using an Eclipse Plus C18 column (1.8 μm, 50 mm × 2.1 mm) with a flow rate of 0.4 mL/min. Mobile phases consisted of H_2_O with 0.1% formic acid (A) and CH_3_CN with 0.1% formic acid (B). Untargeted high-resolution mass spectrometry (HRMS) and data-dependent MS/MS acquisition were conducted in positive ion mode. The gradient method was as follows: 80% A and 20% B held for 5 min, followed by a linear increase of B to 80% over 20 min (5–25 min), then to 90% B over the next 5 min, with a return to initial conditions at 30.01 min. Full-scan MS data were acquired over an *m*/*z* range of 200–1700, and MS/MS data were acquired over an *m*/*z* range of 100–1700. MS/MS spectra were collected in Auto MS/MS mode using a collision energy of 40 V at an acquisition rate of 3 spectra s^–1^, with up to four precursor ions selected per cycle and an isolation width of 1.3 *m*/*z*. Active exclusion was enabled after 2 spectra and the precursor threshold count was set a 10,000. Precursor preference was set to the ‘common organic molecules’ isotope model with a preference for 1 or 2 charge states. Source parameters were as follows: gas temperature, 350 °C; capillary temperature, 300 °C; spray voltage, 3.5 kV; sheath gas flow rate, 11 L min^–1^. Targeted LC-MS/MS analysis of micropeptins used a modified gradient and the same solvents as above consisting of a 5 min isocratic hold at 95% A, followed by a 15 min linear gradient to 100% B, a 5 min hold at 100% B, and re-equilibration starting at 25.01 min. Full-scan MS and MS/MS data were acquired over an *m*/*z* range of 20–2000. Raw data files were exported as mzXML files and uploaded to the GNPS2 Web site using the classical molecular networking workflow (CMN) or feature-based molecular networking (FBMN, details below) workflow and the default parameters in most cases. Initial networks were constructed with a precursor *m*/*z* tolerance and a fragment *m*/*z* tolerance both left as the default parameters 2.0 and 0.5 Da, respectively with a minimum cosine score of 0.6 and two matched peaks and precursor filter of 1. Next after the network was generated, we used the Mass QL^17^ (query language) function available at GNPS2 to search for product ions associated with different cyanopeptide classes (e.g., *m*/*z* 282 and *m*/*z* 404 for micropeptins/cyanopeptolins) and product ions for other reported cyanopeptide groups such as the microcystins. Several new micropeptin structures were proposed based on the analysis of MS/MS spectra, details of which are described in the Results and Discussion section below. Following this first network generation via a small subset of chromatography fractions, we acquired a larger number of chromatography fractions from the Muskegon, MI cyanoHAB collection from Biosortia Microbiomics, which covered a much larger polarity range and contained a more complex portfolio of cyanometabolites. These fractions were subjected to the CMN tandem mass spectrometry workflow and product ion searches were conducted to identify different classes of cyanopeptides. However, precursor *m*/*z* tolerance and fragment *m*/*z* tolerance were both set to 0.02 Da with a minimum cosine score of 0.7 and six matched peaks to create more refined networks and class-level clusters. Tandem mass spectrometry annotations were performed using high-resolution product ion data for fragment assignment. Ambiguous isomers (e.g., Leu or Ile) were noted in proposed structures and annotation confidence levels were as follows: Level 1—metabolite confirmed via reference standard or full NMR characterization; Level 2—mass spectrometry feature strongly supported by HRMS and MS/MS data but not confirmed with authentic standard; Level 3—feature assigned to a compound class or family but isomer potential precludes full structure assignment.

### Feature-Based Molecular Networking (FBMN)

While CMN was initially utilized to provide naïve users with an accessible annotation pipeline, we also applied FBMN to the subset of chromatography fraction library containing the micropeptin clusters shown in Figure 1. Raw mass spectrometry data were converted to.mzML format and preprocessed using MZmine 4.2.0.^20^ Chromatograms were constructed over a retention time window of 5–25 min and deconvoluted using the local minimum resolver algorithm. After isotopic filtering, features alignment and gap-filling, the resulting quantification table and associated MS/MS spectra were exported to GNPS2 for downstream analysis using the feature-based molecular networking workflow and same parameters as described the initial and full chromatography fraction networks described above for CMN.

**Figure 1:**
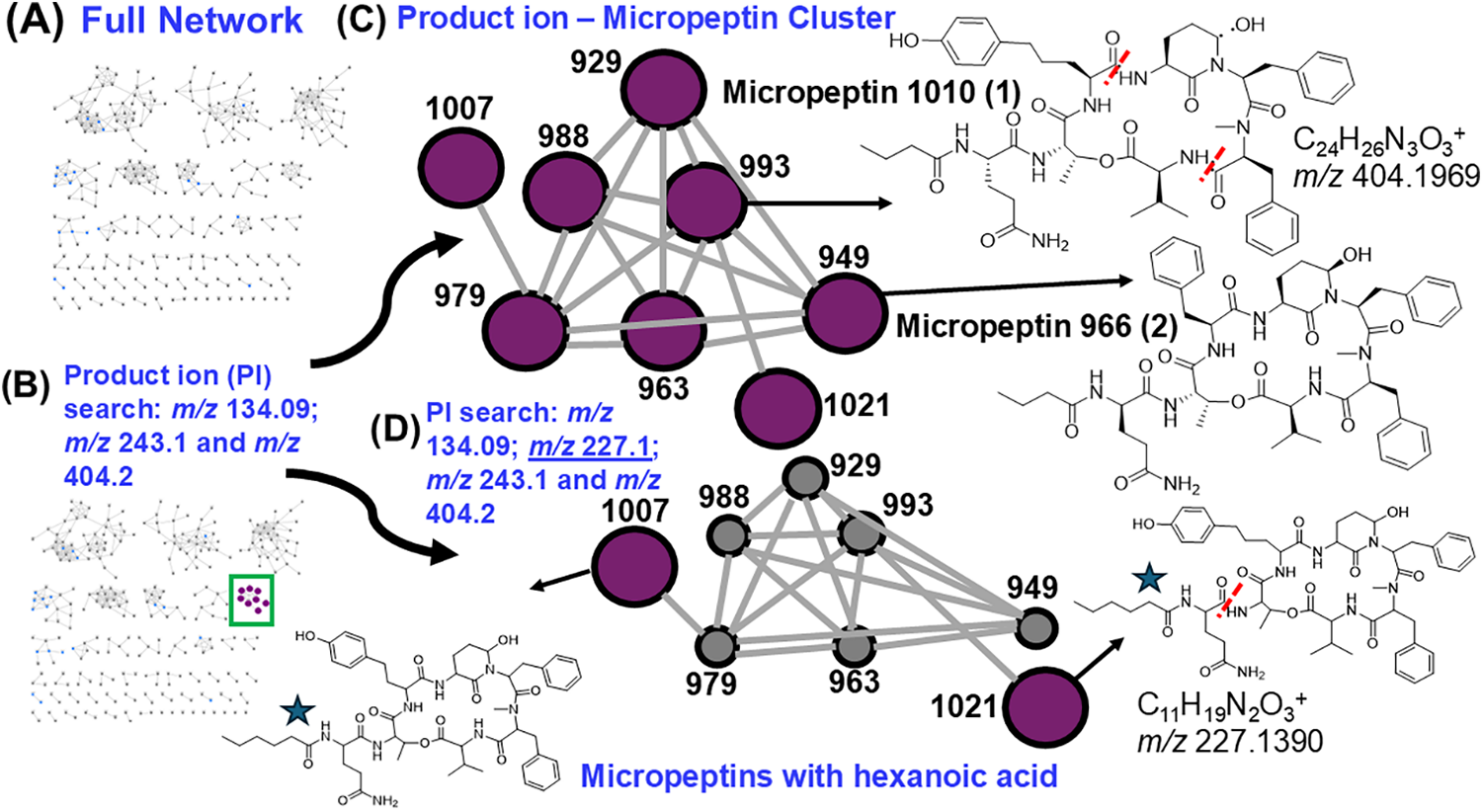
(A) Molecular network derived from analysis of the chromatography fraction library. (B) Product ion search (*m*/*z* 134.09, *m*/*z* 243.1, *m*/*z* 404.2), which highlighted a cluster of micropeptins in the network (purple nodes in the green box). (C) Enhanced view of the micropeptin cluster with new structures shown at right micropeptin 1010 (**1**) and micropeptin 966 (**2**). Red dashed lines show the fragmentation that results in *m*/*z* 404 in **1**. (D) A second product ion search adding in *m*/*z* 227.1, which annotated two putative new micropeptins (purple nodes: node 1021: micropeptin 1038 and node 1007: micropeptin 1024) with hexanoic acid units (blue stars). The red dashed line shows the fragmentation of micropeptin 1038 (*m*/*z* 1021) key in determining the putative structure. All nodes in the networks are designated by an [M+H–H_2_O]^+^ precursor ion.

### Isolation of 1–3 and [Leu^1^]­MC-LR

Preparative amounts of certain chromatography fractions were purchased from Biosortia Microbiomics, which corresponded to the samples analyzed by LC-MS/MS. Following the molecular networking procedures, these fractions were profiled by LC-MS to demonstrate that certain analytes of interest (e.g., *m*/*z* 993 [M+H–H_2_O]^+^, micropeptin 1010) from the molecular networks were present. Micropeptin 1010 (**1**) was isolated from a chromatography fraction using a Kinetex C18 column (5 μm, 250 mm × 10 mm) with H_2_O modified with 0.1% formic acid (solvent A) and CH_3_CN modified with 0.1% formic acid (solvent B). Separation was performed at a flow rate of 3.00 mL/min under isocratic conditions of 65% A and 35% B. A single peak was collected, yielding 4.7 mg of compound **1** (t_R_ 3.4 min).

A second chromatography fraction was reconstituted in CH_3_OH at a concentration of 10 mg/mL and subjected to separation using a YMC ODS column (5 μm, 250 mm × 10 mm). Isocratic elution was performed with 55% A and 45% B at a flow rate of 3.00 mL/min. Seven peaks were collected and analyzed by LC-MS. LC-MS analysis indicated that the first peak consisted of two coeluting components. Repurification of this fraction was performed on a Kinetex C18 column (2.6 μm, 150 mm × 4.6 mm) using an isocratic method consisting of 65% A and 35% B. A subfraction was collected, yielding 0.8 mg of micropeptin 966 (**2**) (t_R_ 9.5 min). The remaining 6 peaks contained known micropeptins and previously characterized compounds (determined via ^1^H NMR, HRMS, and t_R_ values): 0.6 mg of micropeptin 982 (t_R_ 6.3 min, peak 2), 1.0 mg ferintoic acid A (t_R_ 7.4 min, peak 3), 1.0 mg of ferintoic acid C (t_R_ 8.5 min, peak 4, **3**), and 0.9 mg of micropeptin 982 (l-Ser) (t_R_ 9.0 min, peak 5). Peaks 6 and 7 from the initial separation were combined and further purified using the same YMC ODS column under isocratic conditions with 60% A and 40% B. This final purification yielded an 8.4 mg mixture of micropeptin 957 and micropeptin 982 (d-Gln) and a 6.4 mg mixture of micropeptin 950 and micropeptin 996. While ferintoic acid C (**3**) has been characterized via MS/MS,^18^ it has not been unequivocally characterized using NMR, which we accomplished in this report.

A third chromatography fraction with an abundant *m*/*z* 1037 ion signal was subjected to reversed-phase semipreparative HPLC using a Kinetex C18 column (5 μm, 250 mm × 10 mm) with the same A and B solvents as detailed above. Separations were performed at a flow rate of 3.00 mL/min with starting conditions of 68% A and 32% B and a gradient as follows: 32% B to 42% B from 0 to 30 min and 42% B to 100% B from 35 to 40 min, returning to initial conditions at 41 min. A single peak was collected, yielding 6 mg of [Leu^1^]­MC-LR (t_R_ 20.8 min).

### Biological Assays

Neutrophil elastase inhibition was evaluated using a Human Neutrophil Elastase (NE) Inhibitor Screening Kit (Abcam, Cambridge, UK) following the manufacturer’s protocol. Briefly, 50 μL of NE enzyme solution (48 μL of assay buffer and 2 μL of NE stock) was added to each well of a 96-well plate. Subsequently, 25 μL of the test compounds (Compounds **1**, **2** and micropeptin 996, prepared at 4× the desired final concentrations in assay buffer), inhibitor control (SPCK), or assay buffer (enzyme control) were added and incubated at 37 °C for 5 min. Twenty-five μL of a substrate working solution (23 μL of assay buffer and 2 μL of substrate) was added afterward. Test compounds were evaluated across a serial dilution range of 1 nM to 10 μM (final concentrations), with micropeptin 996 included as a positive control. All treatments were performed in technical triplicate. Fluorescence was monitored at λ_ex/em_ = 400/505 nm at 10 min intervals for 30 min using a Tecan Spark multimode plate reader. Initial fluorescence *R*_1_ was recorded at *T*_1_ = 0 min, with the final reading *R*_2_ at *T*_2_ = 30 min. Relative activity was calculated using the following equation: % relative activity = (ΔRFU of compound/ΔRFU of enzyme control) × 100%, where ΔRFU represents the change in fluorescence over 30 min. IC_50_ values were calculated by fitting the data to a four-parameter logistic nonlinear regression model using Prism 9.5.1 (GraphPad, San Diego, CA, USA).

### Collection of Cyanobacterial Field Samples, Toxin Analysis, and Genomic Analysis

Surface water samples were collected from five sites along western Lake Erie on July 23, 2025 (1. Huntington Beach Park, Bay Village, OH, USA, 41.48990°N, 81.93071°W; 2. Miller Road Park, Avon Lake, OH, USA; 41.50275°N, 82.06157°W; 3. Showse Park, Vermilion, OH, USA, 41.43014°N, 82.31420°W; 4. Nickel Plate Beach, Huron, OH, USA, 41.39612°N, 82.54391°W; 5. Huron Harbor North, Huron, OH, USA, 41.39154°N, 82.55421°W), and two samples from East Sandusky Bay (41.45929°N, 82.70725°W) on August 11, 2025, during a cyanoHAB event. At each site, approximately 1 L of surface water was collected into opaque amber high-density polyethylene (HDPE) bottles and transported to the laboratory on ice. Upon arrival, 60 mL aliquots of each sample were filtered through 47 mm, 0.2 μm Sterlitech polyester track etch (PETE) membrane filters. Four replicate filters were prepared per site: two replicates stored at −20 °C for cyanotoxin analysis, and the remaining two replicates stored at −80 °C for DNA extraction and molecular analyses.

Laboratory enrichments were established by inoculating 1 mL of field water into 9 mL of BG-11 medium, with two cultures per site (*n* = 14 total). Cultures were maintained at 22 °C under a 12 h light/12 h dark photoperiod for 3 weeks under 4100K LED lights (15 μmol·photons·m^–2^·s^–1^) which were kept 22 cm from the cultures. Biomass was collected by filtration through 47 mm, 0.2 μm membrane filters. Metabolites from both field samples and laboratory cultures were extracted directly from the filters by three sequential extractions with 2 mL of 75% CH_3_OH each. The combined extracts were concentrated under reduced pressure and reconstituted in 100 μL of CH_3_OH. Samples were then loaded onto preconditioned 100 mg C18 solid-phase extraction (SPE) cartridges and eluted with CH_3_OH. The final eluates were collected in LC-MS vials at a final volume of 1 mL before LC-MS/MS analysis. LC-MS/MS analysis was carried out on the Agilent Revident QTOF as described above with identical molecular networking workflows at GNPS2. For quantification of MP982 and MP996, SPE eluates of field samples and enrichments were dried under a stream of nitrogen and reconstituted to appropriate concentrations in CH_3_OH. Quantification was conducted using parallel reaction monitoring (PRM) mode using the same method and parameters as described above, except that the collision energy was set to 20 V for each transition (MP982: *m*/*z* 965.4767 to 404.1969; MP996: *m*/*z* 979.4924 to 404.1969). Calibration curves were constructed for each compound over a concentration range of 1–100 ppb. Field sample extracts and enrichments were analyzed in duplicate or triplicate for each site, and solvent blanks were injected between sample sets to minimize carryover.

Genomic DNA was extracted from a 10 mL laboratory culture, which showed the presence of micropeptins using the Qiagen DNeasy Plant Mini Kit with minor modifications. Cells were pelleted by centrifugation at 4000*g* for 10 min, resuspended in 400 μL of AP1 buffer with 4 μL RNase A, and disrupted by bead beating for 1 min. Following lysis, DNA was purified and eluted in 200 μL of Buffer AE according to the manufacturer’s protocol. Concentration and purity were assessed using a NanoDrop 2000 spectrophotometer and a Qubit fluorometer prior to sequencing. Purified DNA was sequenced by Plasmidsaurus using the Oxford Nanopore Technologies platform and assembled by Plasmidsaurus into contigs using metaSPADES, which were analyzed via the bioinformatics pipeline described below. We divided the contigs into those over 1000 bp (1182 contigs; mean length 3805 bp; std. dev. 3453 bp; max length 35,178 bp) and those under 1000 bp (98 contigs; mean length 722 bp; std. dev. 142 bp; max length 993 bp). Contigs with GC content between 40%–50%, which were subjected to BLASTp analysis within the Geneious Prime 2019 software platform and a contig of interest with multiple NRPS domains (alignment hit to the *ociB* gene from *Planktothrix* sp.) was identified, which contained homologue to a micropeptin biosynthetic gene.

## Results and Discussion

### Feature-Based Molecular Networking: Mass QL and Product Ion Analysis for Cyanopeptide Annotation and MS/MS Characterization of Micropeptins

MS/MS-based feature-based molecular networking identified molecular features in the initial group of fractions from the Muskegon, MI cyanoHAB chromatography fraction library. We interrogated the network using product ion queries that have been reported for different cyanopeptide groups (Table S1).^21–23^ Intriguingly, this approach was not only able to clearly identify cyanopeptide classes but could also distinguish analog groups within classes in certain cases (Figure 1A–D). Using a Mass QL search of product ions for proposed fragments of [Ahp-Phe-*N*-Me-Phe+H–H_2_O]^+^ (*m*/*z* 404), [Ahp-Phe+H–H_2_O]^+^ (*m*/*z* 243), and the *N*-methyl phenylalanine immonium ion *(m*/*z* 134), we were able to highlight a cluster of molecules in the full network (Figure 1B). Analyzing the MS/MS fragmentation patterns and retention times of these analytes, identified *m*/*z* 979 [M+H–H_2_O]^+^ as micropeptin 996 (l-Gln), a previously characterized compound.^24^ We were able to isolate two of the metabolites in this cluster: *m*/*z* 993 [M+H–H_2_O]^+^ (**1**) and *m*/*z* 949 [M+H–H_2_O]^+^ (**2**) and characterize them as new micropeptins based on MS/MS and NMR data (described below) (Figure 1C).

The ability to identify and annotate new micropeptins via MS/MS, but difficulty in separation and purification, highlights a limitation of high-performance liquid chromatography (HPLC) isolation and nuclear magnetic resonance (NMR)-based characterization. We therefore proposed that more structurally related micropeptins coexist within the fractions yet remain overlooked as evidenced by the number of networks nodes corresponding to analytes that we could not purify and characterize by NMR (Figure 1C), and we conducted thorough HRMS and MS/MS analysis to characterize several new micropeptins (Tables S2 and S3). During repurification of the second chromatographic fraction from which several micropeptins were isolated including **2**, a minor compound was consistently observed coeluting with micropeptin 996 (l-Gln). This compound showed ions of *m*/*z* 973 and *m*/*z* 933, corresponding to [M + Na]^+^ and [M+H–H_2_O]^+^ ions, respectively. Different methods and stationary phases were used to separate the two compounds, but these attempts were unsuccessful. MS/MS comparison of the two network nodes (*m*/*z* 933 and *m*/*z* 979) showed extensive overlapping product ions in a mirror plot (Figure S1). Diagnostic ions at *m*/*z* 560 [butyric acid (BTA)-Gln-Thr-Val-*N*-MePhe+H]^+^ and *m*/*z* 404 [*N*-MePhe-Phe-Ahp+H–H_2_O]^+^ established the conserved scaffold and side chain, leaving one unresolved residue at position 5. The presence of methionine was supported by the immonium ion at *m*/*z* 104, while product ions at *m*/*z* 535 [Met-Ahp-Phe-*N*-MePhe+H–H_2_O]^+^ and *m*/*z* 374 [Met-Ahp-Phe+H–H_2_O]^+^ further confirmed the position of Met (Figure S2,S3). The compound was designated micropeptin 950, with the proposed sequence BTA-Gln-Thr-Met-Ahp-Phe-*N*-MePhe-Val. Delving further into the micropeptin network (Figure 1C), two compounds were rapidly characterized by MS/MS and named as micropeptin 1005 and micropeptin 980, with proposed sequences BTA-Gln-Thr-Trp-Ahp-Phe-*N*-MePhe-Val and BTA-Gln-Thr-Hphe-Ahp-Phe-*N*-MePhe-Val, respectively following careful product ion annotation (Figure S4,S5). We propose Hphe due to the dearth of *N*-methylated residues in micropeptins at position 5 and the common presence of homologated amino acid residues. Another node, characterized by a dehydrated ion at *m*/*z* 929, was named as micropeptin 946 and assigned the sequence BTA-Gln-Thr-Hleu-Ahp-Phe-*N*-MePhe-Val or BTA-Gln-Thr-Hile-Ahp-Phe-*N*-MePhe-Val, as MS/MS fragmentation could not distinguish between leucine and isoleucine (Figure S6). Structural elucidation followed the same approach applied to micropeptin 950: conserved fragments at *m*/*z* 560 and *m*/*z* 404 defined most of the residues in the cyclic scaffold and the side chain, while the residue at position 5 was resolved using HRMS data. Consistent fragmentation patterns across all annotated micropeptins, including ions corresponding to [BTA-Gln-Thr-X+H–H_2_O]^+^ and [X-Ahp-Phe-*N*-MePhe+H–H_2_O]^+^, further supported these assignments. Additional product ion searching incorporating *m*/*z* 227 annotated two molecules in the cluster as putative new micropeptins each proposed to contain a hexanoic acid (HA) starting unit based on key product ions at *m*/*z* 227 [HA-Gln]^+^ and *m*/*z* 310 [HA-Gln-Thr+H–H_2_O]^+^ (Figure 1D) (28 Da higher mass shifts than *m*/*z* 199 and *m*/*z* 282, respectively), which we assigned the names micropeptin 1038 and micropeptin 1024 (Figures S7 and S8).

We next searched for product ions associated with microcystins (*m*/*z* 105.05, *m*/*z* 135.08, and *m*/*z* 213.11).^21^ This highlighted a small cluster in the full network (Figure 2A). We isolated the analyte with *m*/*z* 1037 and characterized it via HRMS (found, 1037.6030; calculated, 1037.6030 for C_52_H_81_N_10_O_12_^+^) and ^1^H NMR as [Leu^1^]­MC-LR (Figures S9 and S10) (Figure 2B),^25^ and the MS/MS pattern comparisons between *m*/*z* 1037 and *m*/*z* 1051 strongly suggested that the *m*/*z* 1051 compound was [Leu^1^,Glu­(OCH_3_)^6^]­MC-LR (Figure 2C).^21^

**Figure 2:**
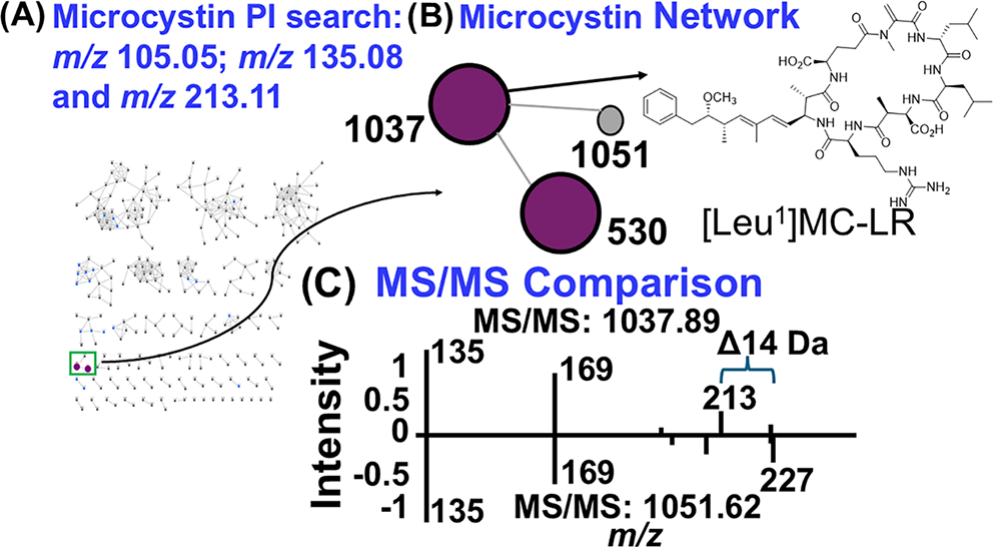
(A) Molecular network of the chromatography fraction library with product ion search for microcystins designated by purple nodes (*m*/*z* 105.05, *m*/*z* 135.08, and *m*/*z* 213.11). (B) Microcystin cluster with *m*/*z* 1037, which was later identified as [Leu^1^]­MC-LR. (C) Comparison of the MS/MS fragmentation patterns of *m*/*z* 1037 and *m*/*z* 1051 in a mirror plot showing a difference of 14 mass units and a *m*/*z* 227 fragment in *m*/*z* 1051 consistent with the fragmentation pattern of [Leu,^1^,Glu­(OCH_3_)^6^]­MC-LR.^15^

### Comparison of Classical and Feature-Based Molecular Networking

The FBMN workflow produced a more refined network compared to classical molecular networking (CMN) for both the subset of chromatography fractions and the full fraction library analysis (Figure S11). Using default GNPS2 parameters (2.0 Da precursor *m*/*z* tolerance, 0.5 Da fragment *m*/*z* tolerance, minimum cosine score of 0.7, and 6 matched peaks), FBMN yielded 143 nodes and 270 edges (excluding singletons), whereas CMN generated a larger and more complex network comprising 190 nodes and 323 edges. The reduction in nodes and edges in FBMN resulted from the removal of artifacts (e.g., column wash features) and the consolidation of redundant isotopic signals into single representative features (Figure S11B), but interestingly increased features in the micropeptin cluster. This may be due to CMN losing lower abundance features when the MS clustering parameter is implemented. Furthermore, the feature reductions were also seen in the full chromatography network (Figure S11D) as well in the micropeptin cluster (Figure S11C).

To facilitate broader feature clustering, the molecular networking thresholds were adjusted to a minimum cosine score of 0.5 and 4 matched peaks, resulting in an expanded network of 328 nodes and 545 edges. Within the micropeptin clusters, a new node emerged at *m*/*z* 916.4809 (Figure S11A). Based on MS/MS fragmentation analysis, this feature was assigned as a new micropeptin with the sequence BTA-Glu-Thr-Leu/Ile-Ahp-Phe-N-MePhe-Val and was named as micropeptin 933 (Figure S12).

Overall, FBMN improves network quality by reducing chemical noise, minimizing false positives, and reducing redundant features in compound discovery. However, it is important to note that FBMN relies on external preprocessing software (e.g., MZmine),^20^ and strict filtering or inappropriate parameter selection during data preprocessing might accidentally remove low-abundance features. We primarily used the feature-based molecular networking workflow here to provide a more accurate network with a comprehensive representation of true molecular diversity. However, given its user-friendly workflow, CMN remains a reasonable approach for broad environmental metabolomics and monitoring applications, but users should be cautioned that CMN often overestimates feature number and can lead to false positive annotation.

### Structure Characterization of **1–3**

Following the MS/MS network annotation, we identified high abundance ions *m*/*z* 1033 [M + Na]^+^ (**1**) and *m*/*z* 989 [M + Na]^+^ (**2**) as putative new micropeptins and targets for isolation and structure elucidation. Compound **1** had a precursor *m*/*z* of 1033.5009 [M + Na]^+^ suggesting a molecular formula of C_53_H_70_N_8_O_12_. A thorough analysis of product ions supported fragments for Ahp-Phe-*N*-Me-Phe–H_2_O (*m*/*z* 404), Ahp-Phe–H_2_O (*m*/*z* 243) and *m*/*z* 282 likely corresponding to a BTA-Gln-Thr–H_2_O fragment (Figure S13), and these fragment data were in harmony with the 1D and 2D NMR data (Figures S14–S19). This left two amino acid positions unassigned, which NMR was able to unequivocally address. A ^1^H–^1^H TOCSY spin system δ_H_ 4.73 (CH), 2.07 (CH), and two methyl signals at δ_H_ 0.87 and 0.73 strongly supported a valine residue which NOE correlations positioned adjacent to the *N*-methyl-Phe. The final amino acid was assigned as bis-homologated tyrosine (bHtyr) based on the TOCSY correlations between three CH_2_ signals in the side chain of a modified Tyr residue (δ_H_ 1.83, 1.41, and 2.40 and 2.35) (Table S4). NOE correlations positioned this residue between the Ahp residue and the threonine. The bHtyr residue was also supported by product ions of *m*/*z* 595 (bHtyr-Ahp-Phe-*N*-Me-Phe–H_2_O) and *m*/*z* 434 (bHtyr-Ahp-Phe-*N*-Me-Phe–H_2_O) (Figure S13). This is the first time the bHtyr has been reported in a micropeptin and we named this molecule micropeptin 1010. Compound **2** gave a high-resolution precursor *m*/*z* value of 989.4737 [M + Na]^+^ supporting a formula of C_51_H_66_N_8_O_11_. NMR analysis assigned a structure nearly identical to **1**, but with a Phe residue in place of the bHtyr residue (cf. Table S5 and Figures S20–S23). Again, this structure was also supported by a product ion at *m*/*z* 551 in the MS/MS spectrum (Phe-Ahp-Phe-*N*-Me-Phe–H_2_O) (Figure S24). We named this compound micropeptin 966. A third molecule was isolated, although it was not present in the first molecular network (**3**). The precursor *m*/*z* of 899.4128 supported a molecular formula of C_46_H_59_N_8_O_9_S (Figure S25) and database searching identified ferintoic acid C as a possible identity. This molecule has been characterized via MS/MS.^18^ The 1D and 2D NMR data were fully supportive of the previously proposed structure including the methionine residue as supported by the correlations observed in the multiplicity-edited HSQC spectrum for δ_H_ 2.08 (CH_3_) and δ_C_ 14.2 (CH_3_) (Figures S26–S29). The amino acid configurations of **1** were all of the l-configuration as determined by Marfey’s analysis (Table S6, Figure S30), while micropeptin 966 (**2**) possessed a d-glutamine (Figure S31) with the remaining amino acids in the l-configuration (Table S6). Ferintoic acid C (**3**) had a d-lysine, which is the convention in this group of molecules, with the remaining amino acids in the l-configuration (Table S6).

### Protease Inhibition Activity

Micropeptins are well-established inhibitors of serine proteases, and our previous work showed that many micropeptins show potent activity against human neutrophil elastase.^5^ To continue this structure–activity relationship, compounds **1** and **2** were evaluated for their inhibitory activity against human neutrophil elastase with micropeptin 996 (l-Gln) used as a positive control, which showed an IC_50_ of 0.52 μM. Micropeptin 1010 (**1**) showed moderate inhibitory activity, with an IC_50_ of 3.4 μM (Figure S32). In contrast, **2** was not active at concentrations up to 10 μM. Micropeptins 996 (l-Gln), **1**, and **2** are closely related analogues that differ only at position 5, which is occupied by homologated tyrosine, bishomologated tyrosine, and phenylalanine, respectively. The observed differences in inhibitory activity suggest that the presence of a phenolic hydroxyl group at this position plays a critical role in protease inhibition, likely by stabilizing key interactions within the protease active site, and its absence results in markedly reduced inhibition.

### Additional Metabolite Annotation Strategies of Cyanopeptides Using Product Ion Searching

After the initial chromatography fraction library molecular networking and micropeptin and microcystin annotation, we analyzed a larger chromatography fraction data set of 99 samples derived from the same Muskegon, MI cyanoHAB material to access more chemical space and additional cyanopeptide groups. We also had a standard library of cyanopeptides from our previous work consisting of microcystins, micropeptins/cyanopeptolins, anabaenopeptins/ferintoic acids, and microviridins for authentication of network hits.^5,19^ The new feature-based network had over 1200 total nodes and 40 nodes in a putative micropeptin/cyanopeptolin network (Figure 3A). Product ion searching via Mass QL could decipher two subgroups in the micropeptin/cyanopeptolin network. Product ion searching using *m*/*z* 209.128 and *m*/*z* 370.21 showed a group of molecules containing either leucine or isoleucine,^23^ while searching for *m*/*z* 243.114 and *m*/*z* 404.198 showed a group with phenylalanine (Figure 3B). We were also able to designate different groups of microcystins one group with [Glu­(OCH_3_)^6^] modifications (product ions: *m*/*z* 135.08 and *m*/*z* 389.2),^21,22^ validated by previously characterized microcystins in our own laboratory standard library (Figure S33).^19^ An anabaenopeptin/ferintoic acid cluster was highlighted by searching product ions *m*/*z* 114.055 and *m*/*z* 405.2,12 which included ferintoic acids A and C (**3**) (Figure S34).^18^ We searched the network for analytes with product ions of *m*/*z* 116.07 and *m*/*z* 159.09 to annotate a cluster of microviridins (Figure S35),^26^ again annotating specific library molecules in these clusters.

**Figure 3:**
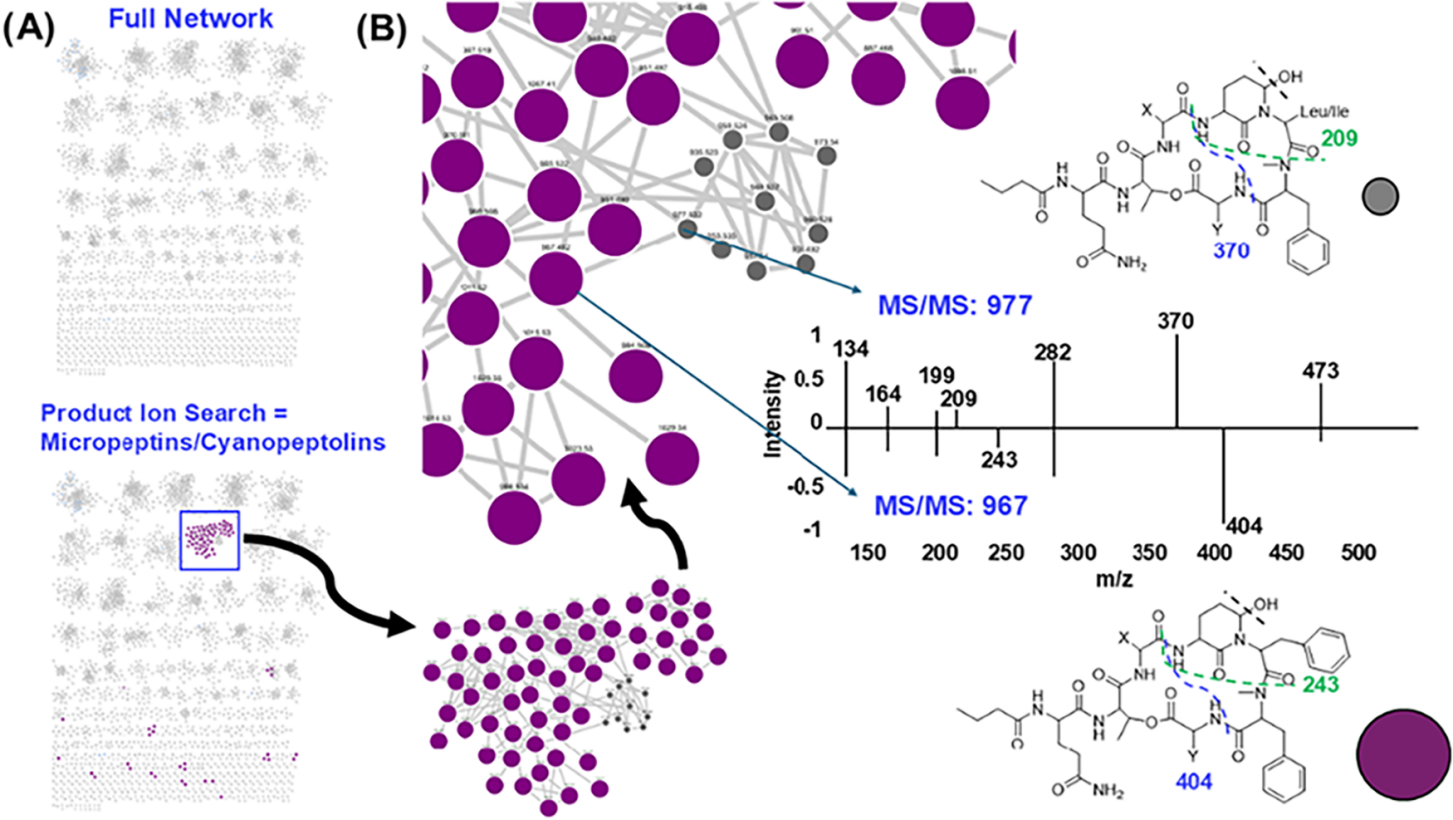
(A) Full network of analytes from the full Muskegon, MI cyanoHAB chromatography library (top network) with product ion searching for cyanopeptolins/micropeptins in the bottom network. (B) Enhanced view of the cyanopeptolin/micropeptin network showing cluster members with product ion *m*/*z* 243 and *m*/*z* 404 (purple nodes). The remaining small gray nodes had a different product ion profile and thus remained as gray nodes (e.g., *m*/*z* 209 and *m*/*z* 370) supporting the presence of a leucine or isoleucine residue versus the phenylalanine residue present in molecules with *m*/*z* 243 and *m*/*z* 404, illustrated with the mirror plot of MS/MS spectra and putative fragmentations shown with dashed lines.

### Cyanopeptides in Field Samples and Enrichments Established from Lake Erie and Analysis of a Metagenome

We screened both initial field collections and enrichments collected from several Lake Erie sites using two pipelines. The first examined HRMS MS1 data against the CyanoMetDB^27^ using the compound identification function available in Agilent’s Mass Explorer software. We assigned a putative ID to analytes showing a scoring mark of 80 or greater and a mass accuracy within 5 ppm. There were 13 putative compound identifications that met these criteria: carmaphycins A & B, [d-Asp]­MC-RF, fisherellin B, lyngbyapeptin D, malyngamide H microcolins A & F, micropeptins 996 and 982, neo-debromoaplysiatoxin H, nostocyclyne A, ribocyclophane D. The second pipeline utilized the LC-MS/MS acquisition with product ion searching and comparison of MS/MS data to literature and the GNPS library. We were able to identify both micropeptin 982 and micropeptin 996 in the molecular network created from the field samples (Figure 4A), and as these standards were both in our library,^28^ we were also able to use these authentic standards for verification (Figure 4B), show that they were in all field samples (Figure 4C), and validate them using the standards that we have accumulated over the years from previous research (Table S7, Figure 4D).^28^ However, we could not unequivocally annotate any other MS1 level cyanopeptide “hits” in the field samples that satisfied MS/MS fragmentation data validation (performed by manual inspection of MS/MS spectra and comparison to literature data or *in silico* predictions) other than these two micropeptins. Following quantification procedures (Figure S36), as shown in Table S8, MP982 and MP996 were detected at ng/L concentrations in field water samples (mean MP982 = 28 ± 12 ng/L), while their concentration increased substantially with biomass enrichment after 21 days. In enrichments, MP982 reached up to 19,000 ng/L after 21 days (Showse Park) and 3000–4000 ng/L after 14 days in samples from East Sandusky Bay (Table S8). These enrichment values are similar to what other groups have found in the field with respect to cyanopeptolins/micropeptins (low μg/L values), and in some cases higher than the concentrations of microcystins previously found in field measurements (mean ± sd = 3.9 ± 3.3 μg/L in Miller et al. 2019).^10^ It is likely that we sampled before the biomass maxima for the Lake Erie bloom and thus found lower concentrations of micropeptins. However, as community dynamics change, the enrichments make it clear that the capacity for greater production is there. Compared to MP982, MP996 was consistently detected at lower abundance in both field and enrichment samples. The two micropeptins showed comparable concentrations across different field sites but were not detected in certain enrichment cultures. This absence may be attributed to variations in community composition, loss of producing strains during cultivation, different growth dynamics, or environmental factors influencing secondary metabolite biosynthesis. Further temporal and spatial resolution will be necessary to fully understand the cyanopeptolin/micropeptin dynamics in Lake Erie.

**Figure 4:**
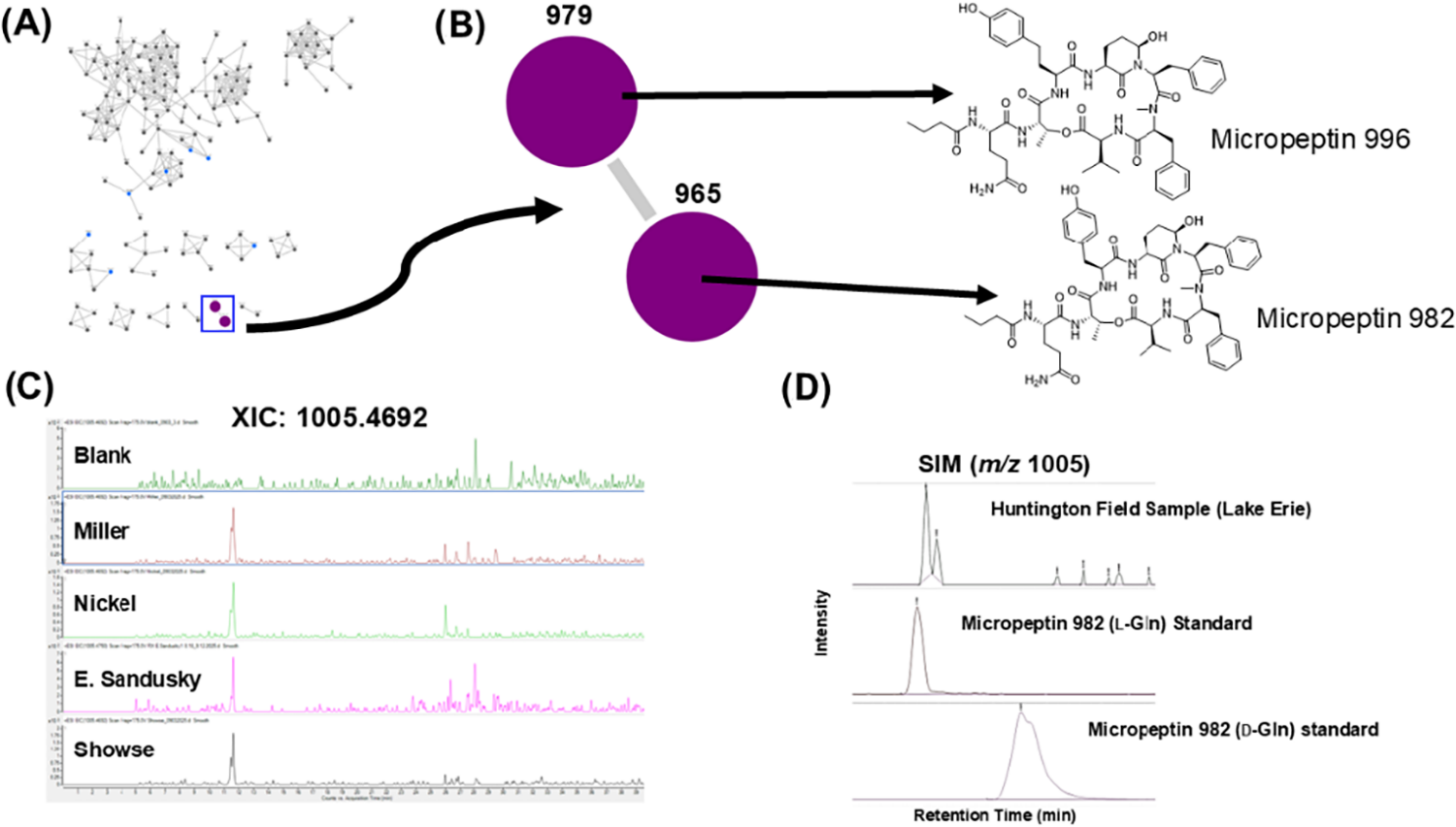
(A) LC-MS/MS-based molecular network of field samples from Lake Erie with micropeptin cluster highlighted in the blue box. (B) Network cluster showing micropeptin nodes [M+H–H_2_O]^+^ and identified micropeptin compounds in our compound library (micropeptin 996 and micropeptin 982). (C) Extracted ion chromatograms of selected field samples for micropeptin 982 (*m*/*z* 1005.4692, [M + Na]^+^). (D) Comparison of Huntington field sample with two authentic standards of micropeptin 982 (l-Gln) and micropeptin 982 (d-Gln) monitoring *m*/*z* 1005.

Metagenomic sequencing and analysis of the sample from Showse Park identified a 2214 bp section that BLAST searching matched to the *ociB* gene in cyanopeptolin biosynthesis from *Planktothrix agardhii* (Figure S37),^29^ supporting a likely connection between the chemistry found from the field sample and the genetic architecture that produces cyanopeptolins/micropeptins. In this work, we investigated the MAG sequence data for orthogonal evidence for the presence of cyanopeptolins/micropeptins, but the data can be further mined for additional community, and biosynthetic information or future field work can also incorporate DNA metabarcoding for community analysis. We are currently trying to establish pure active cultures of the micropeptin producer and other Lake Erie strains in the laboratory.

These results indicate that Great Lakes cyanoHABs and cyanoHABs in other water bodies (e.g., Muskegon, MI) contain diverse nonmicrocystin cyanopeptide chemistry that is largely invisible to routine targeted monitoring. Product ion–driven substructure GNPS2 annotation provides a path to class-level screening of these metabolites in complex bloom samples, enabling mixture assessment, and prioritization for toxicological and treatment studies.

Together, the chemistry, bioactivity, and genetic evidence we provide in this report suggest expanded surveillance beyond canonical toxins to better represent real-world bloom exposure profiles. Cyanopeptolins/micropeptins have shown aquatic toxicity,^30^ but their environmental impact and toxicological profiles are unknown and our SAR approach and large library generation can prioritize metabolites for further *in vitro* and *in vivo* studies. The molecular networks showed multiple cyanopeptide classes (micropeptins, microcystins, anabaenopeptins/ferintoic acids, microviridins) and dozens of individual metabolites in each cluster reinforcing that blooms are chemical mixtures, and the ecological/toxic effects from these events may reflect additivity/synergy/antagonism rather than one compound-one effect paradigms (e.g., microcystin-LR). This can be supported by mixture-aware monitoring and bioassays (effect-directed analysis). While there are thousands of nodes in the full chromatography library network (Figure 3A), it is important to note that the CMN network does overrepresent analyte space due to in-source fragmentation and adduct profiles.^31^ Some of these issues can be abrogated with additional post acquisition workflows as we did with FBMN.^32^ While molecular networking has been used previously in cyanopeptide annotation and substructure searching is routine in natural products chemistry discovery.^18,26,33,34^ However, we use the Mass QL tool to probe and identify subclasses for improved levels of annotation and characterization. To examine how Mass QL application can improve cyanopeptide annotations, we examined the publicly available data used in Kust et al. 2020 (MassIVE: MSV000085840).^33^ We remade networks according to their previously used methods and identified the previously annotated micropeptins: MPT-A, MPT-B, and MPT-SD944 in the initial network (Figure S38). We then utilized product ion searching to differentiate isoleucine or leucine containing micropeptins from phenylalanine containing micropeptins by examining either *m*/*z* 243.1 or *m*/*z* 209.1 product ions. We were able to provide additional annotations for putative cyanopeptolin 1020B (OA-Glu-Thr-Lys-Ahp-Phe-*N*-MeTyr-Val) and cyanopeptolin 1006 (HA-Asp-Thr-Arg-Ahp-Phe-*N*-MeTyr-Val),^35^ and we annotated a putatively new cyanopeptolin/micropeptin 978B (HA-Asp-Thr-Lys-Ahp-Phe-*N*-MeTyr-Val) (Figure S38).

The GNPS2-based product ion annotation may be particularly useful to resource managers and public health officials as it is broadly generalizable across mass spectrometry platforms and reduces the need for standards for every congener providing both untargeted data for continued mining but also preserves an element of targeted analysis with respect to cyanometabolite classes and annotation within classes. Rather than replacing targeted methods, this approach is best viewed as a screening and prioritization tool: product-ion queries can be used to flag the presence of cyanotoxin and cyanopeptide classes (and, where possible, subfamilies) in complex samples, while resource-intensive confirmatory analyses (e.g., MRM methods or standard-based quantification) can be reserved for a limited number of prioritized features. This tiered strategy aligns with the operational realities of HAB surveillance programs, where staffing, instrumentation, and funding often preclude exhaustive chemical characterization and where bloom composition can change rapidly in space and time. At the same time, product ion searching has clear and well recognized limitations, including an inability to resolve isomers, assign stereochemistry, or provide absolute quantitation, and sensitivity to acquisition parameters. In this study, these limitations were addressed through targeted isolation and full structural characterization of selected compounds, including the first identification of bis-homologated tyrosine incorporation in micropeptins, and through the use of a limited in-house standard library to support retention time matching and orthogonal metagenomic evidence for cyanopeptolin/micropeptin biosynthesis. Our environmental sampling was restricted to a short temporal window prior to peak biomass in the Lake Erie bloom,^36^ which likely limited detection of certain toxins, such as microcystins, known to correlate with bloom maxima.^19^ Expanded spatial or temporal sampling could improve chemical coverage, but such efforts may not be feasible for many monitoring programs. Instead, these results suggest that flexible, site specific, and time adaptive suspect screening approaches may be more realistic than fixed toxin panels for routine monitoring. While additional computational and multiomics approaches could further expand annotation depth, their application is likely best suited to research settings, with simplified product ion-based screening in the future providing a practical entry point for routine environmental surveillance.

## Supplementary Material



## Data Availability

NMR data for compounds **1–3** (NP0352196, NP0352197, NP0352198) and [Leu^1^]­MC-LR (NP0352199) have been deposited at https://np-mrd.org/, and tandem mass spectrometry data have been deposited at MassIVE under accession number MSV000100694.

## Notes

Additional experimental details and physical data of **1–3**, NMR data, HRMS, and MS/MS data for compounds **1–3**, additional molecular networks, Marfey’s analysis, bioassay data, calibration curves, and genomic data (PDF)

## References

[1] Wang Y., Zhao D., Woolway R. I., Yan H., Paerl H. W., Zheng Y., Zheng C., Feng L. (2025). Global Elevation of
Algal Bloom Frequency in Large Lakes over the Past Two Decades. Natl. Sci. Rev..

[2] Merder J., Harris T., Zhao G., Stasinopoulos D. M., Rigby R. A., Michalak A. M. (2023). Geographic Redistribution of Microcystin
Hotspots in Response to Climate Warming. Nat.
Water.

[3] Environmental Protection Agency . Drinking Water Health Advisory for the Cyanobacterial Microcytins Toxins, EPA, Office of Water, EPA document number:820R15100, 2015.

[4] Toxic Cyanobacteria in Water . A Guide to Their Public Health Consequences, Monitoring and Management, 2nd ed.; Chorus, I. ; Welker, M. , Eds.; CRC Press, London, 2021.

[5] Xia R., Xie H., Mahmud A. M. S., Bertin M. J. (2025). Alteration in Amino Acid Composition
and Configuration in Cyanobacterial Peptides Affects Biological Activity. J. Nat. Prod..

[6] Yancey C. E., Hart L., Hefferan S., Mohamed O. G., Newmister S. A., Tripathi A., Sherman D. H., Dick G. J. (2024). Metabologenomics
Reveals Strain-Level Genetic and Chemical Diversity of *Microcystis* Secondary Metabolism. mSystems.

[7] Stringer B. B., Szlag Silva R. G., Kodanko J. J., Westrick J. A. (2025). Structure, Toxicity,
Prevalence, and Degradation of Six Understudied Freshwater Cyanopeptides. Toxins.

[8] Janssen E. M.-L. (2019). Cyanobacterial
Peptides beyond Microcystins – A Review on Co-Occurrence, Toxicity,
and Challenges for Risk Assessment. Water Res..

[9] He H., Wu S., Wahome P. G., Wahome P. G., Bertin M. J., Bertin M. J., Pedone A. C., Pedone A. C., Beauchesne K. R., Beauchesne K. R., Moeller P. D. R., Moeller P. D. R., Carter G. T. (2018). Microcystins
Containing Doubly Homologated Tyrosine Residues from a *Microcystis Aeruginosa* Bloom: Structures and Cytotoxicity. J. Nat. Prod..

[10] Miller T. R., Bartlett S. L., Weirich C. A., Hernandez J. (2019). Automated
Subdaily Sampling of Cyanobacterial Toxins on a Buoy Reveals New Temporal
Patterns in Toxin Dynamics. Environ. Sci. Technol..

[11] Wang X., Wullschleger S., Jones M., Reyes M., Bossart R., Pomati F., Janssen E. M.-L. (2024). Tracking Extensive Portfolio of Cyanotoxins
in Five-Year Lake Survey and Identifying Indicator Metabolites of
Cyanobacterial Taxa. Environ. Sci. Technol..

[12] Quandt M. L., Westrick J., Kodanko J. J. (2026). Structure–Activity Relationships
of Anabaenopeptins as Carboxypeptidase and Phosphatase Inhibitors. ACS Chem. Biol..

[13] Faltermann S., Zucchi S., Kohler E., Blom J. F., Pernthaler J., Fent K. (2014). Molecular Effects of
the Cyanobacterial Toxin Cyanopeptolin (CP1020)
Occurring in Algal Blooms: Global Transcriptome Analysis in Zebrafish
Embryos. Aquat. Toxicol..

[14] Gademann K., Portmann C., Blom J. F., Zeder M., Jüttner F. (2010). Multiple Toxin
Production in the Cyanobacterium *Microcystis*: Isolation
of the Toxic Protease Inhibitor Cyanopeptolin 1020. J. Nat. Prod..

[15] Wang M., Carver J. J., Phelan V. V., Sanchez L. M., Garg N., Peng Y., Nguyen D. D., Watrous J., Kapono C. A., Luzzatto-Knaan T., Porto C., Bouslimani A., Melnik A. V., Meehan M. J., Liu W.-T., Crüsemann M., Boudreau P. D., Esquenazi E., Sandoval-Calderón M., Kersten R. D., Pace L. A., Quinn R. A., Duncan K. R., Hsu C.-C., Floros D. J., Gavilan R. G., Kleigrewe K., Northen T., Dutton R. J., Parrot D., Carlson E. E., Aigle B., Michelsen C. F., Jelsbak L., Sohlenkamp C., Pevzner P., Edlund A., McLean J., Piel J., Murphy B. T., Gerwick L., Liaw C.-C., Yang Y.-L., Humpf H.-U., Maansson M., Keyzers R. A., Sims A. C., Johnson A. R., Sidebottom A. M., Sedio B. E., Klitgaard A., Larson C. B., Boya
P C. A., Torres-Mendoza D., Gonzalez D. J., Silva D. B., Marques L. M., Demarque D. P., Pociute E., O’Neill E. C., Briand E., Helfrich E. J. N., Granatosky E. A., Glukhov E., Ryffel F., Houson H., Mohimani H., Kharbush J. J., Zeng Y., Vorholt J. A., Kurita K. L., Charusanti P., McPhail K. L., Nielsen K. F., Vuong L., Elfeki M., Traxler M. F., Engene N., Koyama N., Vining O. B., Baric R., Silva R. R., Mascuch S. J., Tomasi S., Jenkins S., Macherla V., Hoffman T., Agarwal V., Williams P. G., Dai J., Neupane R., Gurr J., Rodríguez A. M.
C., Lamsa A., Zhang C., Dorrestein K., Duggan B. M., Almaliti J., Allard P.-M., Phapale P., Nothias L.-F., Alexandrov T., Litaudon M., Wolfender J.-L., Kyle J. E., Metz T. O., Peryea T., Nguyen D.-T., VanLeer D., Shinn P., Jadhav A., Müller R., Waters K. M., Shi W., Liu X., Zhang L., Knight R., Jensen P. R., Palsson BØ., Pogliano K., Linington R. G., Gutiérrez M., Lopes N. P., Gerwick W. H., Moore B. S., Dorrestein P. C., Bandeira N. (2016). Sharing and Community Curation of Mass Spectrometry
Data with Global Natural Products Social Molecular Networking. Nat. Biotechnol..

[16] Petras D., Phelan V. V., Acharya D., Allen A. E., Aron A. T., Bandeira N., Bowen B. P., Belle-Oudry D., Boecker S., Cummings D. A., Deutsch J. M., Fahy E., Garg N., Gregor R., Handelsman J., Navarro-Hoyos M., Jarmusch A. K., Jarmusch S. A., Louie K., Maloney K. N., Marty M. T., Meijler M. M., Mizrahi I., Neve R. L., Northen T. R., Molina-Santiago C., Panitchpakdi M., Pullman B., Puri A. W., Schmid R., Subramaniam S., Thukral M., Vasquez-Castro F., Dorrestein P. C., Wang M. (2022). GNPS Dashboard: Collaborative Exploration
of Mass Spectrometry Data in the Web Browser. Nat. Methods.

[17] Damiani T., Jarmusch A. K., Aron A. T., Petras D., Phelan V. V., Zhao H. N., Bittremieux W., Acharya D. D., Ahmed M. M. A., Bauermeister A., Bertin M. J., Boudreau P. D., Borges R. M., Bowen B. P., Brown C. J., Chagas F. O., Clevenger K. D., Correia M. S. P., Crandall W. J., Crüsemann M., Fahy E., Fiehn O., Garg N., Gerwick W. H., Gilbert J. R., Globisch D., Gomes P. W. P., Heuckeroth S., James C. A., Jarmusch S. A., Kakhkhorov S. A., Kang K. B., Kessler N., Kersten R. D., Kim H., Kirk R. D., Kohlbacher O., Kontou E. E., Liu K., Lizama-Chamu I., Luu G. T., Luzzatto Knaan T., Mannochio-Russo H., Marty M. T., Matsuzawa Y., McAvoy A. C., McCall L.-I., Mohamed O. G., Nahor O., Neuweger H., Niedermeyer T. H. J., Nishida K., Northen T. R., Overdahl K. E., Rainer J., Reher R., Rodriguez E., Sachsenberg T. T., Sanchez L. M., Schmid R., Stevens C., Subramaniam S., Tian Z., Tripathi A., Tsugawa H., van der Hooft J. J. J., Vicini A., Walter A., Weber T., Xiong Q., Xu T., Pluskal T., Dorrestein P. C., Wang M. (2025). A Universal Language for Finding Mass Spectrometry Data Patterns. Nat. Methods.

[18] Teta R., Della Sala G., Glukhov E., Gerwick L., Gerwick W. H., Mangoni A., Costantino V. (2015). Combined LC–MS/MS and Molecular
Networking Approach Reveals New Cyanotoxins from the 2014 Cyanobacterial
Bloom in Green Lake, Seattle. Environ. Sci.
Technol..

[19] Maurer J. A., Xia R., Kim A. M., Oblie N., Hefferan S., Xie H., Slitt A., Jenkins B. D., Bertin M. J. (2024). Temporal Dynamics
of Cyanobacterial Bloom Community Composition and Toxin Production
from Urban Lakes. ACS ES&T Water.

[20] Schmid R., Heuckeroth S., Korf A., Smirnov A., Myers O., Dyrlund T. S., Bushuiev R., Murray K. J., Hoffmann N., Lu M., Sarvepalli A., Zhang Z., Fleischauer M., Dührkop K., Wesner M., Hoogstra S. J., Rudt E., Mokshyna O., Brungs C., Ponomarov K. (2023). Integrative Analysis of Multimodal Mass Spectrometry Data in MZmine
3. Nat. Biotechnol..

[21] Cottrill K. A., Miles C. O., Krajewski L. C., Cunningham B. R., Bragg W., Boise N. R., Victry K. D., Wunschel D. S., Wahl K. L., Hamelin E. I. (2024). Identification of
Novel Microcystins
in Algal Extracts by a Liquid Chromatography–High-Resolution
Mass Spectrometry Data Analysis Pipeline. Harmful
Algae.

[22] Qi Y., Rosso L., Sedan D., Giannuzzi L., Andrinolo D., Volmer D. A. (2015). Seven New Microcystin
Variants Discovered
from a Native *Microcystis Aeruginosa* Strain – Unambiguous Assignment of Product Ions by Tandem
Mass Spectrometry. Rapid Commun. Mass Spectrom..

[23] McDonald K., Renaud J. B., Pick F. R., Miller J. D., Sumarah M. W., McMullin D. R. (2020). Diagnostic Fragmentation
Filtering for Cyanopeptolin
Detection. Environ. Toxicol. Chem..

[24] Strangman W. K., Stewart A. K., Herring M. C., Wright J. L. C. (2018). Identification
of the new chymotrypsin inhibitor micropeptin 996 by metabolomics-guided
analysis. Tetrahedron Lett..

[25] Schripsema J., Dagnino D. (2002). Complete Assignment
of the NMR Spectra of [D -Leu^1^]-microcystin-LR and Analysis
of Its Solution Structure. Magn. Reson. Chem..

[26] McDonald K., DesRochers N., Renaud J. B., Sumarah M. W., McMullin D. R. (2023). Metabolomics
Reveals Strain-Specific Cyanopeptide Profiles and Their Production
Dynamics in *Microcystis Aeruginosa* and *M. flos-aquae*. Toxins.

[27] Janssen, E. M.-L. ; Jones, M. R. ; Pinto, E. ; Dörr, F. ; Torres, M. A. ; Jacinavicius, F. R. ; Mazur-Marzec, H. ; Szubert, K. ; Konkel, R. ; Tartaglione, L. ; Dell’Aversano, C. ; Miglione, A. ; McCarron, P. ; Beach, D. G. ; Miles, C. O. ; Fewer, D. P. ; Sivonen, K. ; Jokela, J. ; Wahlsten, M. ; Niedermeyer, T. H. J. ; Schanbacher, F. ; Pedro, L. ; Preto, M. ; D’Agostino, P. M. ; Baunach, M. ; Dittmann, E. ; Miguel-Gordo, M. ; Reher, R. ; Sieber, S. S75 | CyanoMetDB | Comprehensive database of secondary metabolites from cyanobacteria (NORMAN-SLE-S75.0.3.0) [Data set]. 2024, 10.5281/zenodo.13854577.

[28] Kirk R. D., He H., Wahome P. G., Wu S., Carter G. T., Bertin M. J. (2021). New Micropeptins
with Anti-Neuroinflammatory Activity Isolated from a Cyanobacterial
Bloom. ACS Omega.

[29] Rounge T. B., Rohrlack T., Tooming-Klunderud A., Kristensen T., Jakobsen K. S. (2007). Comparison of Cyanopeptolin Genes
in *Planktothrix*, *Microcystis*, and *Anabaena* Strains:
Evidence for Independent Evolution within Each Genus. Appl. Environ. Microbiol..

[30] de
Almeida Torres M., Jones M. R., vom Berg C., Pinto E., Janssen E. M. L. (2023). Lethal and Sublethal Effects towards Zebrafish Larvae
of Microcystins and Other Cyanopeptides Produced by Cyanobacteria. Aquat. Toxicol..

[31] Xue J., Domingo-Almenara X., Guijas C., Palermo A., Rinschen M. M., Isbell J., Benton H. P., Siuzdak G. (2020). Enhanced In-Source
Fragmentation Annotation Enables Novel Data Independent Acquisition
and Autonomous METLIN Molecular Identification. Anal. Chem..

[32] Nothias L.-F., Petras D., Schmid R., Dührkop K., Rainer J., Sarvepalli A., Protsyuk I., Ernst M., Tsugawa H., Fleischauer M., Aicheler F., Aksenov A. A., Alka O., Allard P.-M., Barsch A., Cachet X., Caraballo-Rodriguez A. M., Da Silva R. R., Dang T., Garg N., Gauglitz J. M., Gurevich A., Isaac G., Jarmusch A. K., Kameník Z., Kang K. B., Kessler N., Koester I., Korf A., Le Gouellec A., Ludwig M., Martin H. C., McCall L.-I., McSayles J., Meyer S. W., Mohimani H., Morsy M., Moyne O., Neumann S., Neuweger H., Nguyen N. H., Nothias-Esposito M., Paolini J., Phelan V. V., Pluskal T., Quinn R. A., Rogers S., Shrestha B., Tripathi A., van der
Hooft J. J. J., Vargas F., Weldon K. C., Witting M., Yang H., Zhang Z., Zubeil F., Kohlbacher O., Böcker S., Alexandrov T., Bandeira N., Wang M., Dorrestein P. C. (2020). Feature-Based Molecular Networking in the GNPS Analysis
Environment. Nat. Methods.

[33] Kust A., Řeháková K., Vrba J., Maicher V., Mareš J., Hrouzek P., Chiriac M.-C., Benedová Z., Tesařová B., Saurav K. (2020). Insight into Unprecedented
Diversity of Cyanopeptides in Eutrophic Ponds Using an MS/MS Networking
Approach. Toxins.

[34] Berger T., Alenfelder J., Steinmüller S., Heimann D., Gohain N., Petras D., Wang M., Berger R., Kostenis E., Reher R. (2024). A MassQL-Integrated
Molecular Networking Approach for the Discovery
and Substructure Annotation of Bioactive Cyclic Peptides. J. Nat. Prod..

[35] Czarnecki O., Henning M., Lippert I., Welker M. (2006). Identification of Peptide
Metabolites of *Microcystis* (Cyanobacteria) That Inhibit
Trypsin-like Activity in Planktonic Herbivorous *Daphnia* (Cladocera). Environ. Microbiol..

[36] National Centers for Coastal Ocean Science . 2025 Lake Erie Harmful Algal Bloom Seasonal Assessment. 2025.

