# Supplementary material for: LC-MS/MS Strategy Using Substructure Searching for the Annotation of Cyanopeptide Classes: Implications for New Compound Discovery and Environmental Monitoring
Source: Environ Sci Technol. 2026 Jun 20;60(26):18545–54. doi: 10.1021/acs.est.6c05607 (PMC13348029; doi:10.1021/acs.est.6c05607)
Supplement: Supplementary file 1 [file es6c05607_si_001.pdf]

Supporting Information: LC-MS/MS Strategy using Substructure Searching for the Annotation of Cyanopeptide Classes: Implications for New Compound Discovery and Environmental Monitoring

Runjie Xia,[a] Lindsey Ahn,[a] Michaela Burkhauser,[a] Ross Youngs,[b] and Matthew J. Bertin[a,]*

[a]Department of Chemistry, Case Western Reserve University, Cleveland, OH 44106, United States

[b]Biosortia, Inc., 2545 Farmers Dr., Suite 370, Columbus, OH 43235, United States

**Corresponding author. Email address: mxb1224@case.edu*

Contents

Additional Experimental Details……………………………………………………………S2-S4

Tables………………………………………………………………………………………S8-S17

Figures……………………………………………………………………………………S18-S55

References…………………………………………………………………………………..S56

Additional Experimental Procedures and Physical Data

General Experimental Procedures. Optical rotation values were measured using a Jasco P-2000 polarimeter. NMR spectra were recorded on a Bruker 500 MHz Ascend Advance III NMR instrument. Chemical shifts reported for **1-3** were referenced to the residual solvent peak of $(CD_3)_2SO$ (δ_H 2.50 and δ_C 39.5). LC-HRMS and LC-HRMS^2 data were collected on an Agilent Revident QTOF mass spectrometer equipped with a Jet Stream source and 1290 Infinity II Bio LC (with multisampler and multicolumn thermostat) and MassHunter Workstation software. Additional LC-MS analyses were conducted on an Agilent LC-MSD single-quadrupole mass spectrometer equipped with an Agilent 1260 HPLC system with autosampler. Semipreparative HPLC separations were carried out using an Agilent 1260 Infinity system equipped with a vacuum degasser, autosampler, and diode array detector.

Configuration Analysis of 1-3. To determine the absolute configuration of the α-amino acids in compounds **1–3**, 0.4 mg of each compound was hydrolyzed in 0.5 mL of 6 N HCl at 110°C for 16 h. After cooling to room temperature, the hydrolysates were dried under a stream of nitrogen, reconstituted in 100 µL of water and 100 µL of 1 M $NaHCO_3$. Derivatization was performed by the addition of 500 µL of a 1% (w/v) solution of *N*-α-(2,4-dinitro-5-fluorophenyl)-L-valinamide (L-FDVA) in acetone. The reaction mixtures were stirred and heated at 40°C for 1 h, then quenched by the addition of 50 µL of 2 N HCl. The derivatized hydrolysates were subsequently diluted 1:10 with a 1:1 mixture of H_2O/CH_3CN to a final volume of 1 mL. Separations were performed on a Luna C18 column (5 µm, 150 × 2.0 mm) using a linear gradient of water (A) and acetonitrile (B), each modified with 0.1% formic acid, at a flow rate of 0.4 mL/min. The gradient was programmed from 20% to 50% B over 30 min, followed by a return to initial conditions from 31 to 36 min. Absolute configurations were assigned by comparison of retention times with those of L-FDVA-

derivatized authentic amino acid standards prepared under identical conditions. Using this approach, all proteinogenic amino acid residues were unambiguously assigned. For the bishomologated tyrosine (bHtyr) residue present in compound **1**, an authentic D-bHtyr standard was not available. To resolve its configuration, two aliquots of compound **1** (0.25 mg each) were independently hydrolyzed as described above and derivatized using 1% solutions of *N*-α-(2,4-dinitro-5-fluorophenyl)-L-leucinamide (L-FDLA) and *N*-α-(2,4-dinitro-5-fluorophenyl)-D-leucinamide (D-FDLA) in acetone, respectively. The resulting derivatives were analyzed by LC-MS using the same column, with a modified gradient elution from 20% to 80% B over 30 min, followed by re-equilibration to initial conditions from 31 to 36 min. Configuration was assigned based on the interaction of the derivatives with the reversed-phase column chemistry as has been previously described in cyanopeptide analysis.[1]

Physical Data for 1-3

Micropeptin 1010 (**1**): colorless oil; $[\alpha]^{21}_{D}$ = -27.4 (*c* 0.05, MeOH); UV λ_{max}: 223, 280 nm (from HPLC-DAD); ^{1}H NMR (500 MHz, DMSO-d_6) and ^{13}C NMR (125 MHz, DMSO-d_6) see Table S1, HRMS (ESI-QTOF) *m/z* calcd for $C_{53}H_{70}N_8O_{12}Na^+$: 1033.5005 $[M+Na]^+$; found 1033.5009 (error 0.39 ppm).

Micropeptin 966 (D-Gln) (**2**): colorless oil; UV λ_{max}: 280 nm (from HPLC-DAD); ^{1}H NMR (500 MHz, DMSO-d_6) and ^{13}C NMR (125 MHz, DMSO-d_6) see Table S2, HRMS (ESI-QTOF) *m/z* calcd for $C_{51}H_{66}N_8O_{11}Na^+$: 989.4743 $[M+Na]^+$; found 989.4771 (error 2.83 ppm).

Ferintoic acid C (**3**): colorless oil; UV λ_{max}: 220, 280 nm (from HPLC-DAD); ^{1}H NMR (500 MHz, DMSO-d_6) δ Trp^1: 4.00 (CH, m), 3.15, 2.99 (CH_2, m), 7.48 (CH, d, *J*=7.9 Hz), 6.90 (CH, t, *J*=7.4 Hz), 7.00 (CH, ovlp), 7.28 (CH, d, *J*=8.1 Hz), 7.03 (CH, ovlp), 6.04 (NH, m), 10.66 (NH, s); Lys^2: 4.01 (CH, m), 1.55 (CH_2, m), 1.28, 1.15 (CH_2, m), 1.42 (CH_2, m), 3.53, 2.77 (CH_2, m),

6.50 (NH, m); Phe[3]: 4.36 (CH, m), 3.26, 2.74 (CH_2, m), 7.05 (2CH, d, *J*=7.2 Hz), 7.20 (2CH, t, *J*=7.3 Hz), 7.16 (CH, d, *J*=7.2 Hz), 8.53 (NH, d, *J*=8.9 Hz); *N*-Me-Ala[4]: 4.77 (CH, m), 1.06 (CH_3, d, *J*=6.6 Hz), 1.76 (*N*CH_3, s); Htyr[5]: 4.75 (CH, m), 1.88, 1.73 (CH_2, m); 2.63, 2.43 (CH_2, m), 7.00 (2CH, d, *J*=8.5 Hz), 6.68 (2CH, d, *J*=8.0 Hz), 9.01 (NH, d, *J*=5.0 Hz); Met[6]: 4.20 (CH, m), 1.99 (CH_2, m), 2.62 (CH_2, m), 2.08 (CH_3, s); ^{13}C NMR (125 MHz, DMSO-d_6) δ Trp[1]: 54.5 (CH), 27.5 (CH_2), 118.5 (CH), 117.5 (CH), 120.0 (CH), 110.6 (CH), 123.0 (CH); Lys[2]: 54.5 (CH), 31.8 (CH_2), 19.5 (CH_2), 27.8 (CH_2), 37.5 (CH_2); Phe[3]: 54.6 (CH), 37.2 (CH_2, m), 128.4 (2CH), 128.0 (2CH), 125.8 (CH); *N*-Me-Ala[4]: 54.0 (CH), 13.4 (CH_3), 26.5 (NCH_3); Htyr[5]: 48.2 (CH), 32.9 (CH_2), 28.9 (CH_2), 128.8 (2CH), 114.7 (2CH); Met[6]: 51.8 (CH), 31.1 (CH_2), 28.9 (CH_2), 14.2 (CH_3). HRMS (ESI-QTOF) *m/z* calcd for $C_{46}H_{59}N_8O_9S^+$: 899.4120 $[M+H]^+$; found 899.4128 (error 0.89 ppm).

__Descriptions of Tables and Figures__

Table S1. Product ions used to define each cyanopeptide class and structural origins.

Table S2. Molecular formulas, exact masses, and high-resolution mass spectrometric data for newly identified micropeptins and other cyanopeptides and confidence level of identification.

Table S3. Sequences of micropeptin variants isolated/identified in this study (BTA=butanoic acid, HA=hexanoic acid).

Table S4. NMR Data for Micropeptin 1010 (**1**).

Table S5. NMR data for micropeptin 966 (D-Gln) (**2**).

Table S6. Marfey's derivatization data and assignments for **1–3.**

Table S7. Retention times of micropeptin stereoisomers and field sample peaks under LC-MSD conditions.

Table S8. Concentration of micropeptin 982 and micropeptin 996 in field samples and enrichments.

Figure S1. Mirror plot of MS/MS spectra of micropeptin 950 and micropeptin 996.

Figure S2. HRMS measurement (*m/z* 973.4456) and annotated MS/MS fragmentation pattern of micropeptin 950.

Figure S3. Annotated MS/MS spectrum of micropeptin 950.

Figure S4. Annotated MS/MS spectrum of micropeptin 1005.

Figure S5. Annotated MS/MS spectrum of micropeptin 980.

Figure S6. Annotated MS/MS spectrum of micropeptin 946.

Figure S7. Annotated MS/MS spectrum of micropeptin 1038.

Figure S8. Annotated MS/MS spectrum of micropeptin 1024.

Figure S9. Isolation and characterization of [Leu^1]MC-LR.

Figure S10. ^1H NMR (500 MHz, MeOH-d_4) of [Leu^1]MC-LR.

Figure S11. CMN vs. FBMN.

Figure S12. Annotated MS/MS spectrum of micropeptin 933.

Figure S13. Mass spectrometry data of **1**.

Figure S14. ^{1}H NMR (500 MHz, DMSO-d_6) of micropeptin 1010 (**1**).

Figure S15. ^{13}C NMR (125 MHz, DMSO-d_6) of micropeptin 1010 (**1**).

Figure S16. Multiplicity-edited HSQC of micropeptin 1010 (**1**).

Figure S17. HMBC of micropeptin 1010 (**1**).

Figure S18. TOCSY of micropeptin 1010 (**1**).

Figure S19. NOESY of micropeptin 1010 (**1**).

Figure S20. ^{1}H NMR (500 MHz, DMSO-d_6) of micropeptin 966 (D-Gln) (**2**).

Figure S21. Multiplicity-edited HSQC of micropeptin 966 (D-Gln) (**2**).

Figure S22. TOCSY of micropeptin 966 (D-Gln) (**2**).

Figure S23. NOESY of micropeptin 966 (D-Gln) (**2**).

Figure S24. Mass spectrometry data of **2.**

Figure S25. HRMS of ferintoic acid C *m/z* 899.4128 $[M+H]^+$.

Figure S26. ^{1}H NMR (500 MHz, DMSO-d_6) of ferintoic acid C (**3**).

Figure S27. Multiplicity-edited HSQC of ferintoic acid C (**3**).

Figure S28. TOCSY of ferintoic acid C (**3**).

Figure S29. NOESY of ferintoic acid C (**3**).

Figure S30. LC-MS analysis of the hydrolysate of **1** reacted with L-FDLA (top panel) and a mixture of L- and D-FDLA (bottom panel) to determine the configuration of the bHtyr in **1**.

Figure S31. LC-MS analysis of the hydrolysate of **2**.

Figure S32. Activity of micropeptin 996 (L-Gln), micropeptin 1010 (**1**), and micropeptin 966 (D-Gln) (**2**) against human neutrophil elastase.

Figure S33. Microcystin cluster in MS/MS molecular network subjected to two different product ion searches.

Figure S34. MS/MS cluster of anabaenopeptins/ferintoic acids annotated via product ion searching.

Figure S35. Microviridin cluster annotated using product ion searching in MS/MS networks.

Figure S36. Calibration curves of micropeptin 982 and 996.

Figure S37. Blast hit to the *ociB* gene (cyanopeptolin biosynthetic pathway) in the metagenomic sequence data from Lake Erie (Showse Park).

Figure S38. Mass QL annotations of original networks from Kust et al. 2020.

Table S1. Product ions used to define each cyanopeptide class and structural origins.

Class of Compounds	Product Ion (*m/z*)	Structural Origin
**Microcystin**	389.20	[Adda-Glu(OCH_3)-Mdha+H–134]$^+$
	375.19	[Adda-Glu-Mdha+H–134]$^+$
	213.09	[Glu-Mdha+H]$^+$
	135.08	[Ph-CH_2-CH(OMe)]$^+$
	105.10	[Ph-CH_2-CH+H]$^+$
**Ferintoic acid**	405.21	[Lys-Phe-*N*Me-Ala+CO+H_2O]$^+$
**Anabaenopeptin**	114.06	[*N*MeAla+CO+H]$^+$
**Micropeptin / Cyanopeptolin**	404.20	[*N*Me-Phe-Phe-Ahp+H–H_2O]$^+$
	370.21	[NMe-Phe–Leu/Ile–Ahp+H–H_2O]$^+$
	310.18	[HA-Gln-Thr+H–H_2O]$^+$
	282.14	[BTA-Gln-Thr+H–H_2O]$^+$
	227.14	[HA-Gln]$^+$
	199.10	[BTA-Gln]$^+$
	243.11	[Phe–Ahp+H–H_2O]$^+$
	209.13	[Leu/Ile–Ahp+H–H_2O]$^+$
	134.10	*N*Me-Phe immonium ion
**Microviridin**	159.09	Trp immonium ion
	116.07	[Asp+H]$^+$

Table S2. Molecular formulas, exact masses, high-resolution mass spectrometric measurements, and retention times for newly identified micropeptins and other cyanopeptides and confidence level of identification.

Name	Molecular formula	Calcd $[M+H–H_2O]^+$	Exact mass	Calcd $[M+H]^+$ or $[M+Na]^+$	Found	Mass error (ppm)	t_R (min)	Confidence Level
MP 1010	$C_{53}H_{70}N_8O_{12}$	993.5080	1010.5113	1033.5005	1033.5009	0.39	12.18	1
MP 966 (D-Gln)	$C_{51}H_{66}N_8O_{11}$	949.4818	966.4851	989.4743	989.4771	2.83	11.92	1
MP 980	$C_{52}H_{68}N_8O_{11}$	963.4975	980.5008	1003.4900	1003.4903	0.30	12.34	2
MP 950	$C_{47}H_{66}N_8O_{11}S$	933.4539	950.4572	973.4464	973.4456	-0.82	11.33	2
MP 1005	$C_{53}H_{67}N_9O_{11}$	988.4927	1005.496	1028.4852	1028.4856	0.39	12.91	2
MP 946	$C_{49}H_{70}N_8O_{11}$	929.5131	946.5164	969.5056	969.5060	0.41	13.13	3
MP 933	$C_{48}H_{67}N_7O_{12}$	916.4815	933.4848	956.4745	916.4809	-0.76	13.03	3
MP 1038	$C_{55}H_{74}N_8O_{12}$	1021.5393	1038.5426	1061.5318	1061.5318	0.00	13.98	2
MP 1024	$C_{54}H_{72}N_8O_{12}$	1007.5237	1024.5270	1047.5162	1047.5167	0.48	13.20	2
[Leu^1[MC-LR]	$C_{52}H_{80}N_{10}O_{12}$		1036.5957	1037.6030	1037.6030	0.00	10.80	1
[Leu^1,Glu(OCH_3)^6] MC-LR	$C_{53}H_{82}N_{10}O_{12}$		1050.6114	1051.6192	1051.6188	-0.38	11.74	2
Ferintoic acid C	$C_{46}H_{58}N_8O_9S$		898.4047	899.4120	899.4128	0.89	10.88	1

Table S3. Sequences of micropeptin variants isolated/identified in this study (BTA=butanoic acid, HA=hexanoic acid).

	Residue 1	Residue 2	Residue 3	Residue 4	Residue 5	Residue 6	Residue 7	Side chain
MP 996	Val	*N*Me-Phe	Phe	Ahp	Htyr	Thr	Gln	BTA
MP 982	Val	*N*Me-Phe	Phe	Ahp	Tyr	Thr	Gln	BTA
MP 982 (L-Ser)	Val	*N*Me-Phe	Phe	Ahp	Htyr	Ser	Gln	BTA
MP 957	Val	*N*Me-Trp	Phe	Ahp	Val	Thr	Gln	BTA
MP 1010	Val	*N*Me-Phe	Phe	Ahp	bHtyr	Thr	Gln	BTA
MP 966 (D-Gln)	Val	*N*Me-Phe	Phe	Ahp	Phe	Thr	Gln	BTA
MP 980	Val	*N*Me-Phe	Phe	Ahp	Hphe	Thr	Gln	BTA
MP 950	Val	*N*Me-Phe	Phe	Ahp	Met	Thr	Gln	BTA
MP 1005	Val	*N*Me-Phe	Phe	Ahp	Trp	Thr	Gln	BTA
MP 946	Val	*N*Me-Phe	Phe	Ahp	Hleu/ HIle	Thr	Gln	BTA
MP 933	Val	*N*Me-Phe	Phe	Ahp	Leu/ Ile	Thr	Glu	BTA
MP 1038	Val	*N*Me-Phe	Phe	Ahp	bHtyr	Thr	Gln	HA
MP 1024	Val	*N*Me-Phe	Phe	Ahp	Htyr	Thr	Gln	HA

Table S4. NMR Data for Micropeptin 1010 (**1**) (500 MHz for ^{1}H NMR, 125 MHz for ^{13}C NMR; DMSO-d_6)

Position	*δ*C, mult	*δ*H, mult, *J* (Hz)	TOCSY	ROESY
**Val-1**				
2	55.3, CH	4.73, ovlp	3, NH	
3	30.3, CH	2.07, m	2, 4, 5, NH	
4	18.9, CH_3	0.87, ovlp	3, 5, NH	
5	16.8, CH_3	0.73, d (6.4)	3, 4, NH	
NH		7.41, ovlp	2, 3, 4, 5	*N*-MePhe-2, *N*-Me
***N*-MePhe-1**				
2	60.1, CH	5.03, ovlp	3a, 3b	Val-NH, Phe-2
3a	33.3, CH_2	3.23, m	2	
3b		2.84, m	2	
4				
5/9	129.1, CH	7.25, d (7.3)	6, 7, 8	Phe-2
6/8	128.3, CH	7.40, t (7.3)	5, 7, 9	
7	126.3, CH	7.31, t (7.2)	5, 6, 8, 9	
N-Me	30.0, CH_3	2.79, s		Val-NH
**Phe-1**	170.8, C			
2	49.7, CH	4.73, ovlp	3a, 3b	*N*-MePhe-2, 5, 9, Ahp-5
3a	34.9, CH_2	2.83, m	2, 3b	Ahp-5
3b		1.65, m	2, 3a	Ahp-5
4				
5/9	129.1, CH	6.78, d (7.4)	6, 7, 8	Ahp-5
6/8	127.5, CH	7.18, t (7.3)	5, 7, 9	
7	125.9, CH	7.13, d (7.4)	5, 6, 8, 9	
**Ahp-1**				
2	48.2, CH	3.58, m	3a, 3b, 4a, 4b, 5, NH	Ahp-2, 3b, 4b
3a	21.2, CH_2	2.38, m	2, 3b, 4a, 4b, 5, NH	Ahp-NH
3b		1.56, m	2, 3a, 4b, NH	Ahp-2
4a	28.7, CH_2	1.66, m	2, 3a, 4b, 5, NH	Ahp-3a, 5
4b		1.48, m	2, 3a, 4a, 5, NH	Ahp-2, 5
5	73.2, CH	5.03, ovlp	2, 3a, 4a, 4b, OH	Ahp-4a, 4b, Phe-2, 3a, 5, 9
NH		7.10, ovlp	2, 3a, 3b, 4a, 4b	Ahp-3a, bHtyr-2, bHtyr-NH
OH		6.05, br	5	
**bHtyr-1**				
2	51.3, CH	4.22, m	3, 4, 5a, 5b, NH	Ahp-NH
3	29.1 CH_2	1.83, m	2, 4, 5a, 5b, NH	
4	27.1 CH_2	1.41, m	2, 3, 5a, 5b, NH	
5a	33.4, CH_2	2.40, m	2, 3, 4, 5b	
5b		2.35, m	2, 3, 4, 5a	
6	132.4, C			
7/9	128.9, CH	6.91, d (8.2)	7, 9	
8/10	114.7, CH	6.64, d (8.2)	6, 8	
11	155.8, C			
NH		8.42, d (8.7)	2, 3, 4	Ahp-NH, Thr-2, 3
**Thr-1**				
2	54.2, CH	4.62, m	NH	bHtyr-NH
3	71.6, CH	5.41, m	4	bHtyr-NH
4	17.3, CH_3	1.17, d (6.4)	3	
NH		7.91, d (9.2)	2	Gln-2, 3a
**Gln-1**				
2	51.7, CH	4.37, m	3a, 3b, 4, NH	Thr-NH
3a	27.3, CH_2	1.90, m	2, 3b, 4, NH	Thr-NH
3b		1.71, m	2, 3a, 4, NH	
4	31.4, CH_2	2.17, m	2, 3a, 3b, NH	

5				
NH		8.05, d (7.7)	2, 3a, 3b, 4	BTA-2
NH_2		7.27, br<br>6.75, br		
**BTA-1**				
2	36.6, CH_2	2.12, m	3, 4	Gln-NH
3	18.4. CH_2	1.53, m	2, 4	
4	13.3, CH_3	0.88, ovlp	2, 3	

Table S5. NMR data for micropeptin 966 (D-Gln) (**2**) (500 MHz for ^{1}H NMR, 125 MHz for ^{13}C NMR; DMSO-d_6).

Position	δC, mult	δH, mult, *J* (Hz)	TOCSY	NOESY
**Val-1**				
2	54.2, CH	4.74, m	3, 4, 5, NH	
3	30.3, CH	2.07, m	2, 4, 5, NH	
4	18.7, CH_3	0.86, d (7.3)	2, 3, 5, NH	
5	16.8, CH_3	0.72, d (6.8)	2, 3, 4, NH	
NH		7.44, ovlp[a]	2, 3, 4, 5	*N*-MePhe-2
***N*-MePhe-1**				
2	60.6, CH	5.04, m	3a, 3b	Val-NH, Phe-2, 3a
3a	33.6, CH_2	3.23, m	2	
3b		2.87, m	2	
4				
5/9	129.2, CH	7.25, d (7.5)	6, 7, 8	Phe-3a
6/8	128.3, CH	7.42, t (7.5)	5, 7, 9	
7	125.7, CH	7.32, d (7.4)	5, 6, 8, 9	
N-Me	30.0, CH_3	2.79, s		
**Phe^1-1**				
2	49.5, CH	4.75, ovlp	3a, 3b	*N*-MePhe-2, Ahp-5, NH
3a	34.7, CH_2	2.83, m	2	*N*-MePhe-5, 9, Ahp-5
3b		1.67, m	2	Ahp-5
4				
5/9	129.2, CH	6.78, d (7.6)	6, 7, 8	
6/8	127.5, CH	7.17, t (7.3)	5, 7, 9	
7	125.9, CH	7.13, d (7.3)	5, 6, 8, 9	
**Ahp-1**				
2	48.4, CH	3.60, m	3a, 3b, 4a, NH	Ahp-3a, 3b, Ahp-NH
3a	20.9, CH_2	2.40, m	2, 3b, 4a, 4b, 5, NH	Ahp-NH
3b		1.59, m	2, 3a	Ahp-2
4a	29.2, CH_2	1.66, ovlp	2, 3a, 4b, NH	Ahp-3a, 5, OH
4b		1.51, m	4a, 5, NH	Ahp-5
5	73.5, CH	5.04, ovlp	3a, 4a, 4b, OH	Ahp-4a, 4b, Phe^1-2, 3a, 3b, 4a
NH		7.10, d (9.3)	2, 3a, 4a, 4b	Ahp-2, 3a, Phe^2-NH
OH		6.06, br	5	Ahp-4a
**Phe^2-1**				
2	48.4, CH	4.32, m	3a, 3b, NH	
3a	36.9, CH_2	2.64, m	2, 3b, NH	
3b		1.79, m	2, 3a, NH	
4				
5/9	123.8, CH	7.11, ovlp	6, 8	
6/8	122.6, CH	7.08, ovlp	5, 7	
7	122.2, CH	6.91, m		
NH		8.59, d (8.3)	2, 3a, 3b	Ahp-NH, Thr-2, 3
**Thr-1**				
2	54.6, CH	4.53, m	NH	Phe-NH
3	71.3, CH	5.39, m	4	Phe-NH
4	17.5, CH_3	1.18, d (6.5)	3	
NH		7.91, m	2	Gln-2
**Gln-1**				
2	52.1, CH	4.41, m	3a, 3b, 4, NH	Thr-NH
3a	27.6, CH_2	1.89, m	2, 3b, 4, NH	
3b		1.75, m	2, 3a, 4, NH	
4	31.3, CH_2	2.11, m	2, 3a, 3b, NH	
5				
NH		8.10, d (8.0)	2, 3a, 3b, 4	BTA-2

NH_2		7.28, br<br>6.78, br		
**BTA-1**				
2	36.8, CH_2	2.13, m	3, 4	Gln-NH
3	18.4. CH_2	1.54, m	2, 4	
4	13.1, CH_3	0.89, d (7.3)	2, 3	

[a]overlapping signals

Table S6. Marfey's derivatization data and assignments for **1–3.**

Amino Acid	t_R (min)	Ferintoic acid C (3)	Micropeptin 966 (D-Gln) (2)	Micropeptin 1010 (1)
L-Glutamine	13.36			
D-Glutamine	16.99			
L-Glutamic acid	15.10			15.12 (L)
D-Glutamic acid	16.61		16.64 (D)<br>(from D-Gln)	
L-Serine	13.09			
D-Serine	13.92			
L-Tryptophan	25.11	25.14 (L)		
D-Tryptophan	28.68			
L-Valine	20.86		20.59 (L)	20.67 (L)
D-Valine	27.66			
L-*N*-Me-Phenylalanine	26.12		25.74 (L)	25.81 (L)
D-*N*-Me Phenylalanine	27.48			
L-Phenylalanine	25.44	25.09 (L)	25.00 (L)	25.12 (L)
D-Phenylalanine	30.95			
L-Homotyrosine	21.45	20.87 (L)		
D-Homotyrosine	24.37			
L-Methionine	20.52	20.96 (L)		
D-Methionine	26.22			
L-Threonine	12.86		12.47 (L)	12.54 (L)
L-allo-Threonine	13.66			
D-allo-Threonine	15.73			
D-Threonine	17.66			
L-*N*-Me-Alanine	18.57	18.66 (L)		
D-*N*-Me-Alanine	18.98			
L-Lysine	6.20			
D-Lysine	5.56	5.58 (D)		
Bis-homotyrosine (L-FDLA)	17.61			(L)
Bis-homotyrosine (D-FDLA)	18.89			

Table S7. Detection of micropeptins in Lake Erie samples. Retention times of micropeptin stereoisomers and field sample peaks under LC-MSD conditions (Luna C18, 150 × 2 mm, 5 µm, 55% A / 45% B, 0.6 mL/min).

Sample / Standard	Name	*m/z*	t_R (min)
Standard	Micropeptin 982 (L-Gln)	1005	1.80
Standard	Micropeptin 982 (L-Ser)	1005	2.56
Standard	Micropeptin 982 (D-Gln)	1005	2.72
Standard	Micropeptin 982 (L-*allo*-Thr)	1005	2.90
Field Extract (Huntington)	Micropeptin 982 (L-Gln)	1005	1.86
Standard	Micropeptin 996 (L-Gln)	1019	2.75
Standard	Micropeptin 996 (D-Gln)	1019	2.86
Field Extract (Huntington)	Micropeptin 996 (L-Gln)	1019	2.78

Table S8. Concentration of micropeptin 982 and micropeptin 996 in field samples and enrichments.

	MP982 (Field, ng/L)	MP996 (Field, ng/L)	MP982 (Enrichment, ng/L)	MP996 (Enrichment, ng/L)
Huntington Beach	24	10	340	ND
Miller Road Park	23	9	ND	ND
Showse Park	21	7	19000	160
Nickel Plate Beach	20	16	650	ND
Huron Harbor North	36	9	360	ND
East Sandusky Bay-1	52	8	3000	870
East Sandusky Bay-2	18	13	4000	1800

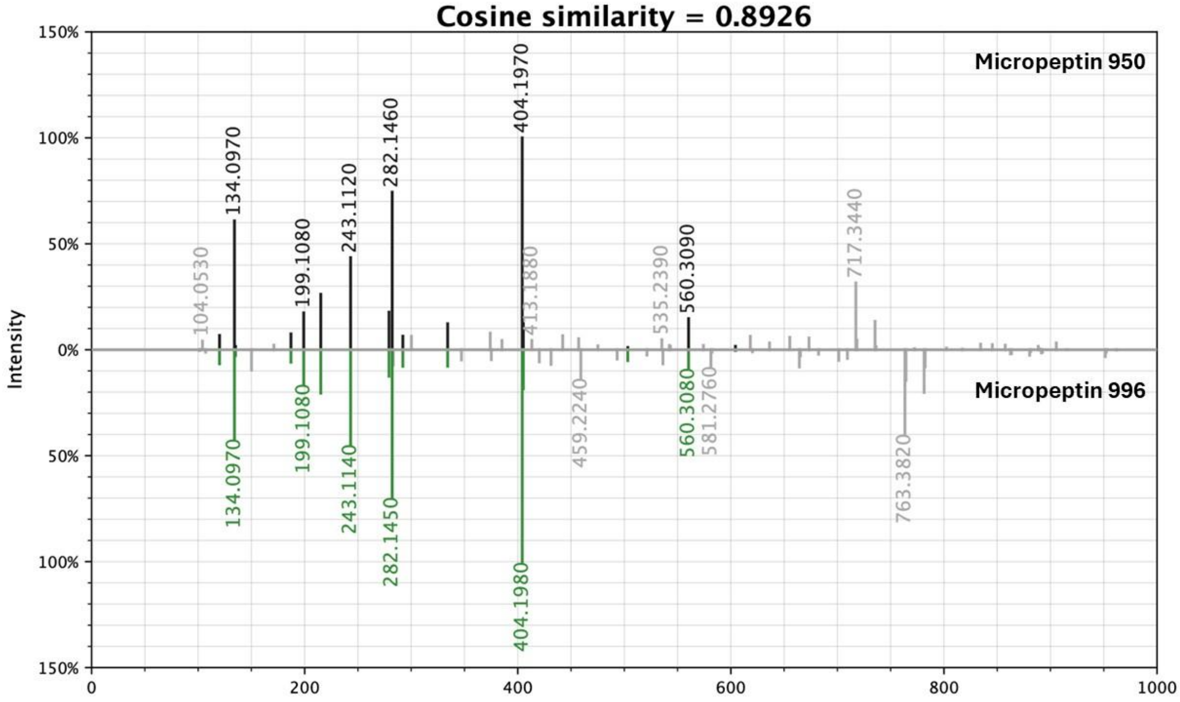

Figure S1. Mirror plot of MS/MS spectra of micropeptin 950 and micropeptin 996.

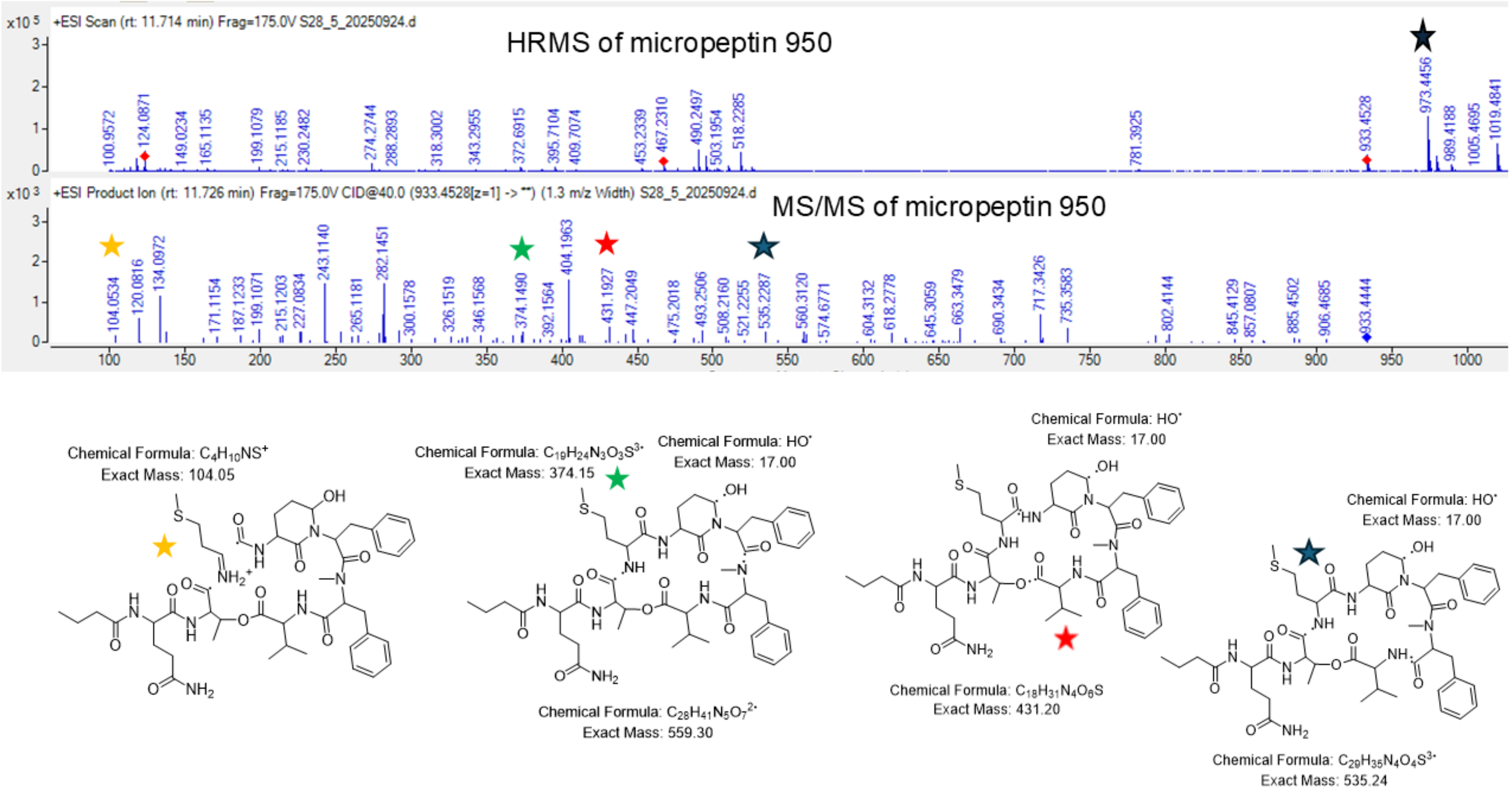

Figure S2. HRMS measurement (*m/z* 973.4456) and annotated MS/MS fragmentation pattern of micropeptin 950.

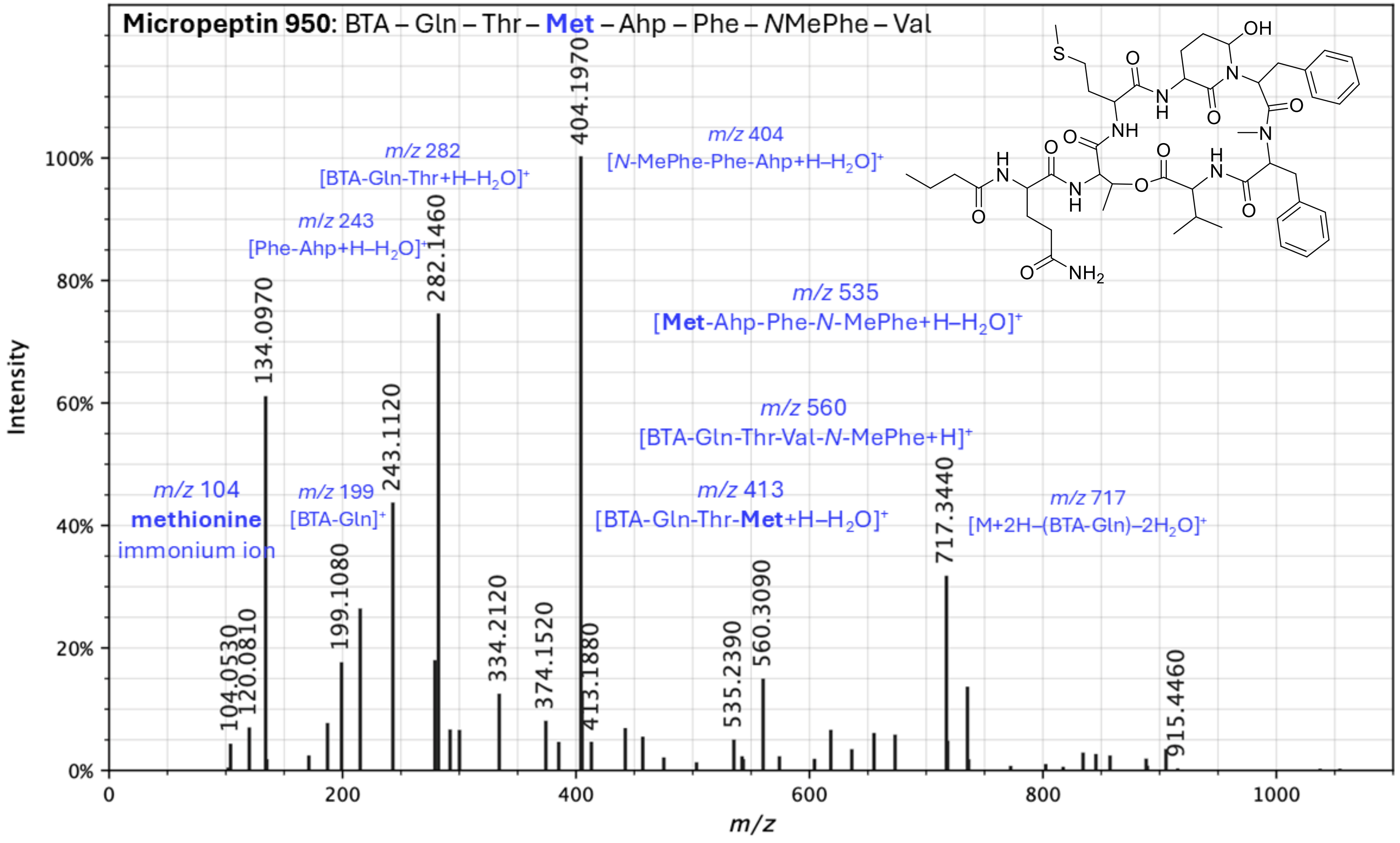

Figure S3. Annotated MS/MS spectrum of micropeptin 950.

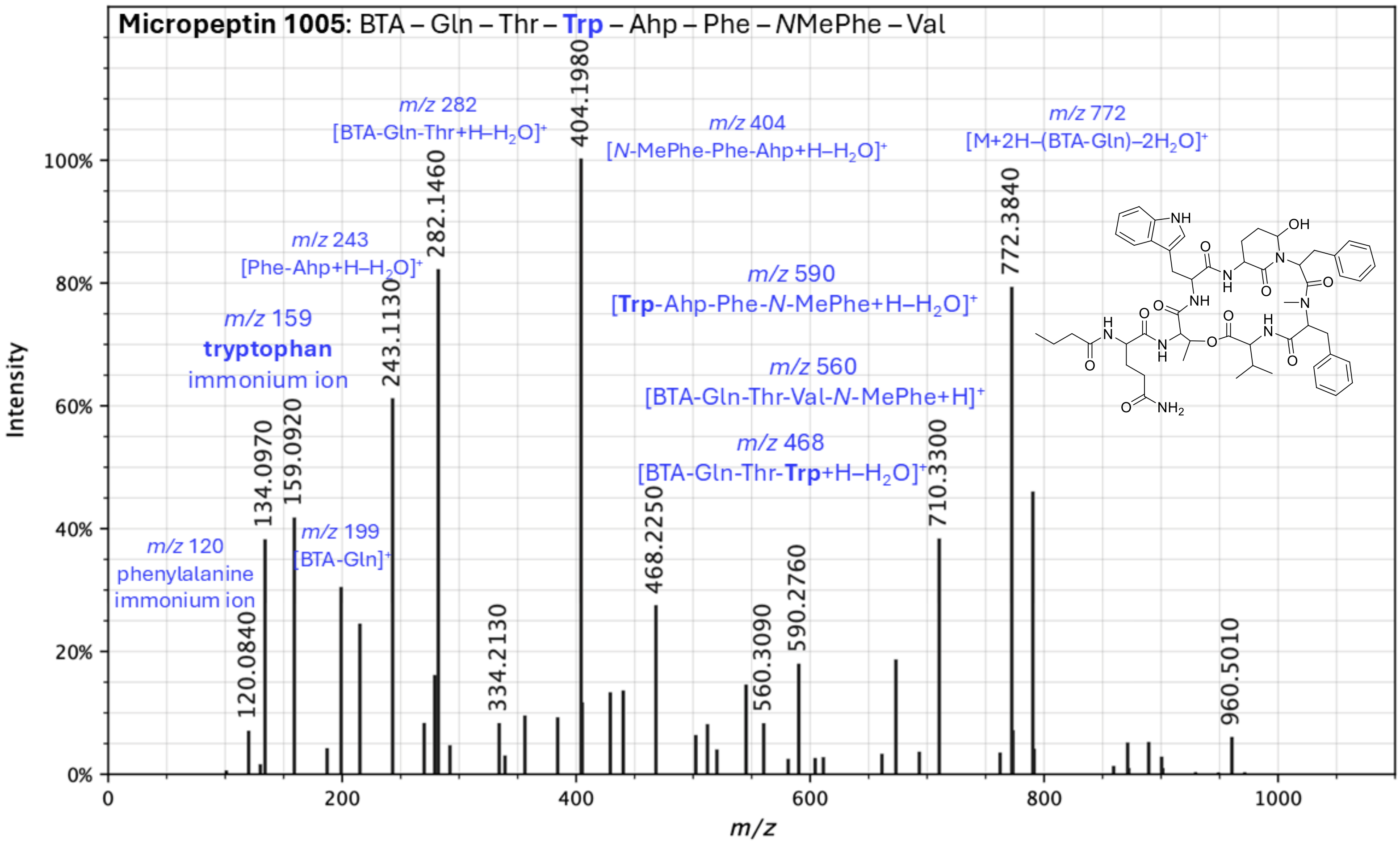

Figure S4. Annotated MS/MS spectrum of micropeptin 1005.

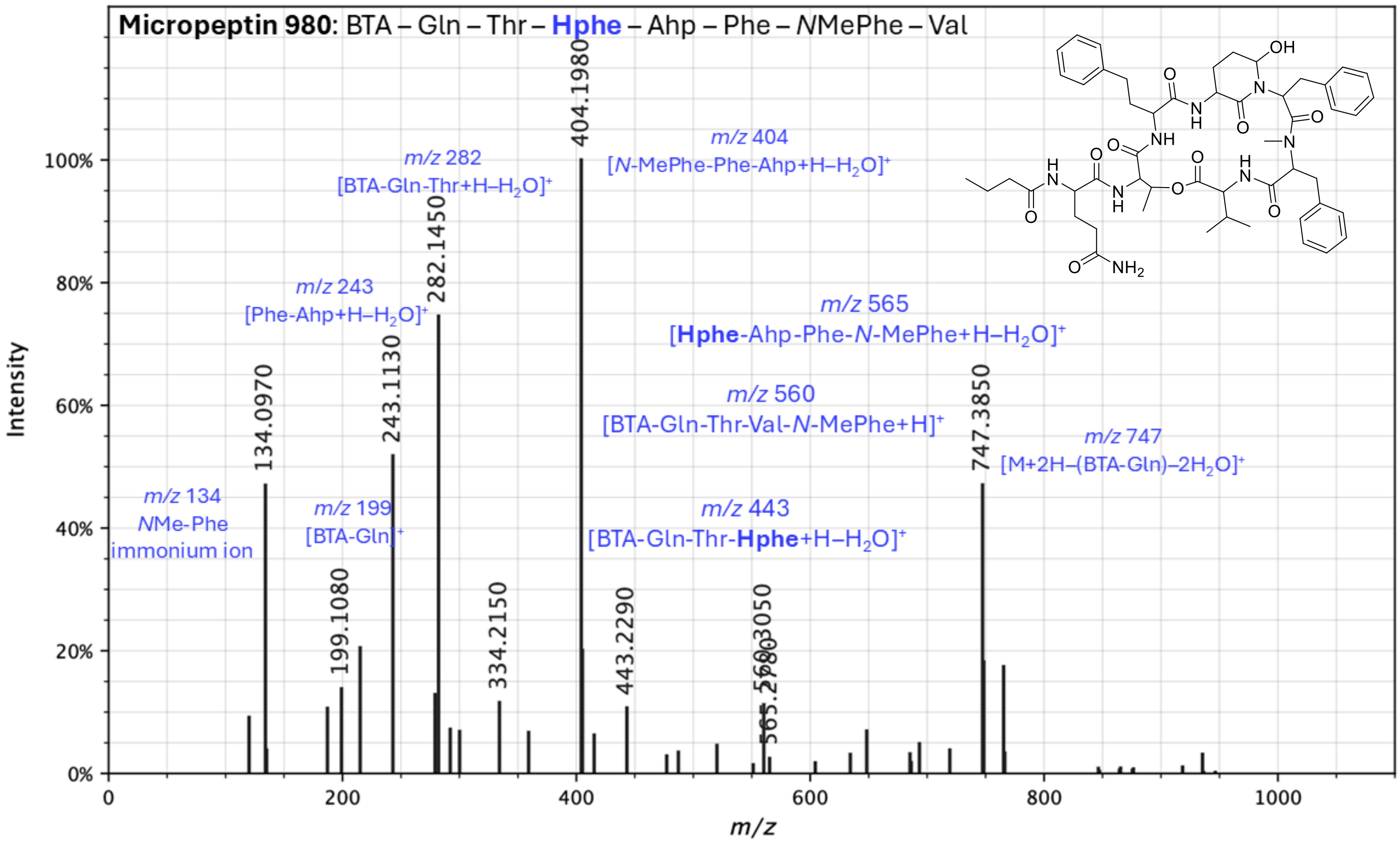

Figure S5. Annotated MS/MS spectrum of micropeptin 980.

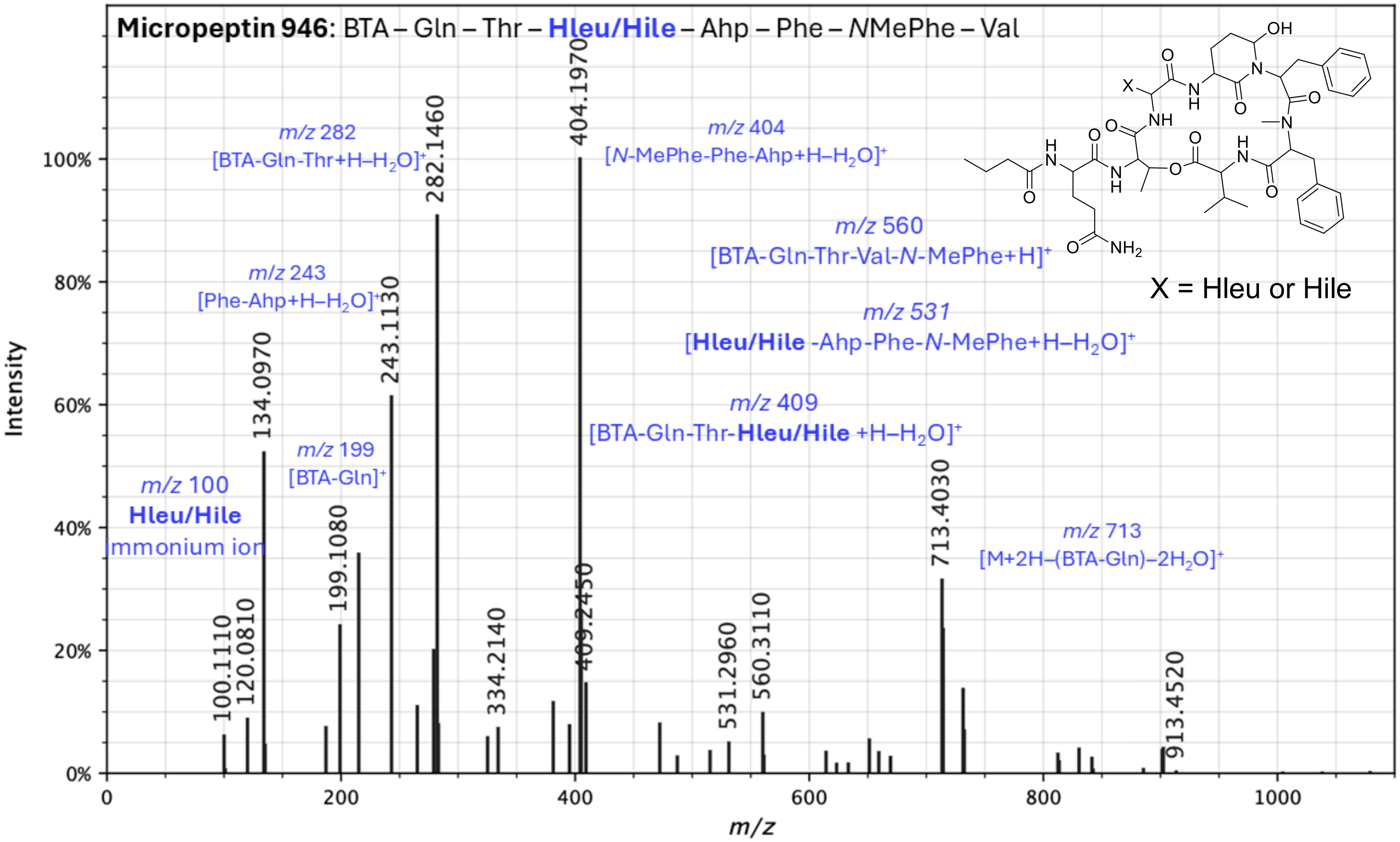

Figure S6. Annotated MS/MS spectrum of micropeptin 946.

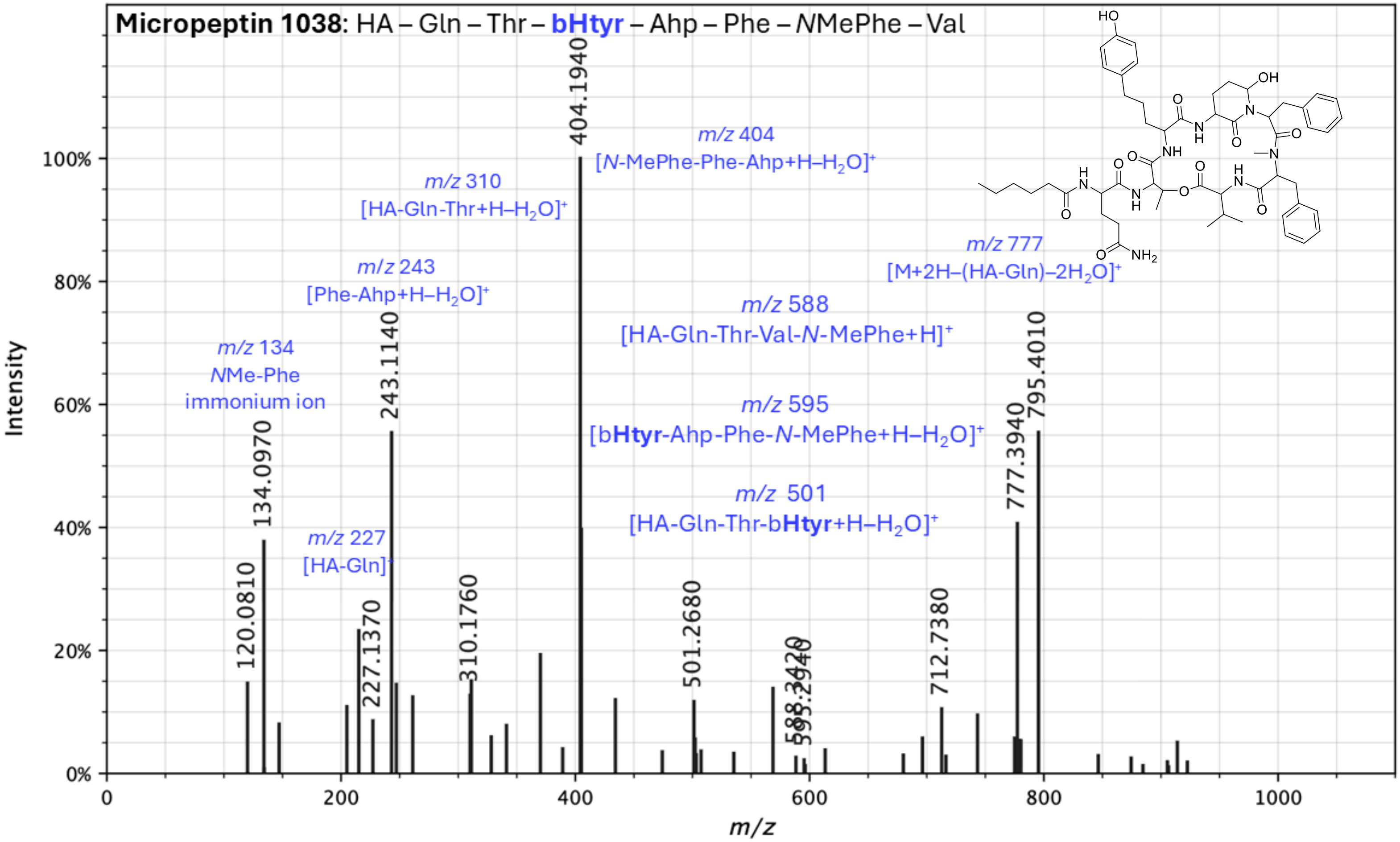

Figure S7. Annotated MS/MS spectrum of micropeptin 1038.

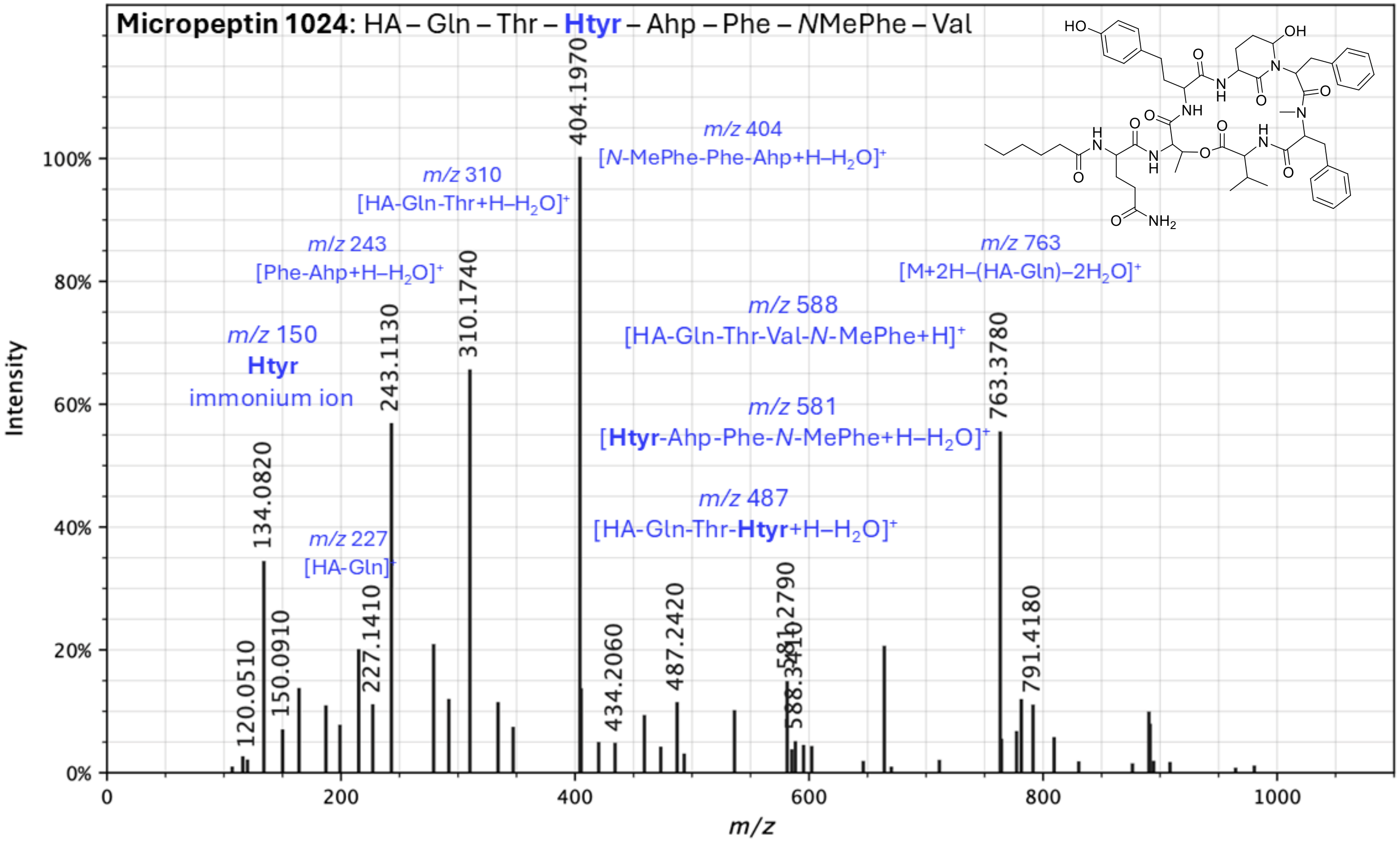

Figure S8. Annotated MS/MS spectrum of micropeptin 1024.

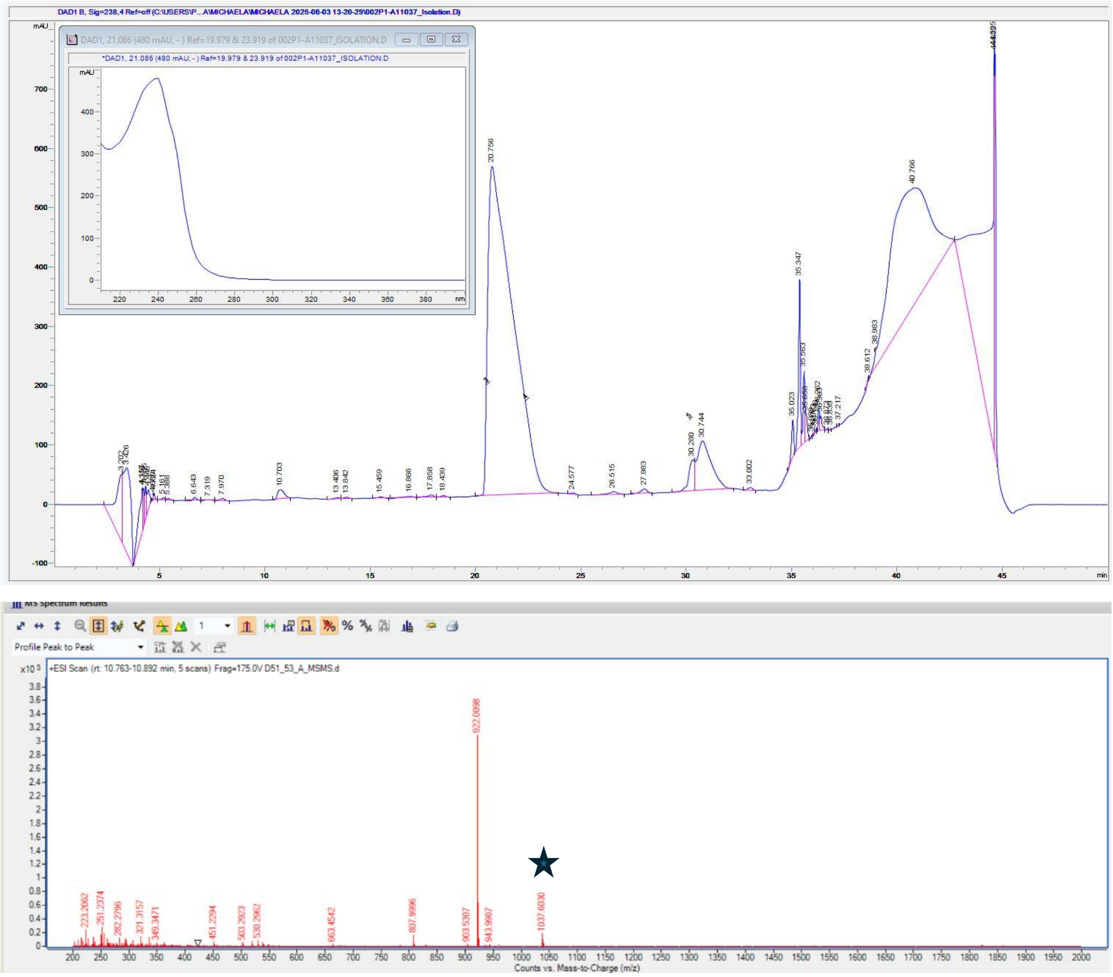

Figure S9. Isolation and characterization of [Leu[1]]MC-LR. Top panel: HPLC-DAD analysis with UV spectrum with λ_{max} of 238 nm (inset) of [Leu[1]]MC-LR. Bottom panel: LC-HRMS analysis of [Leu[1]]MC-LR with *m/z* 1037.6030 (star).

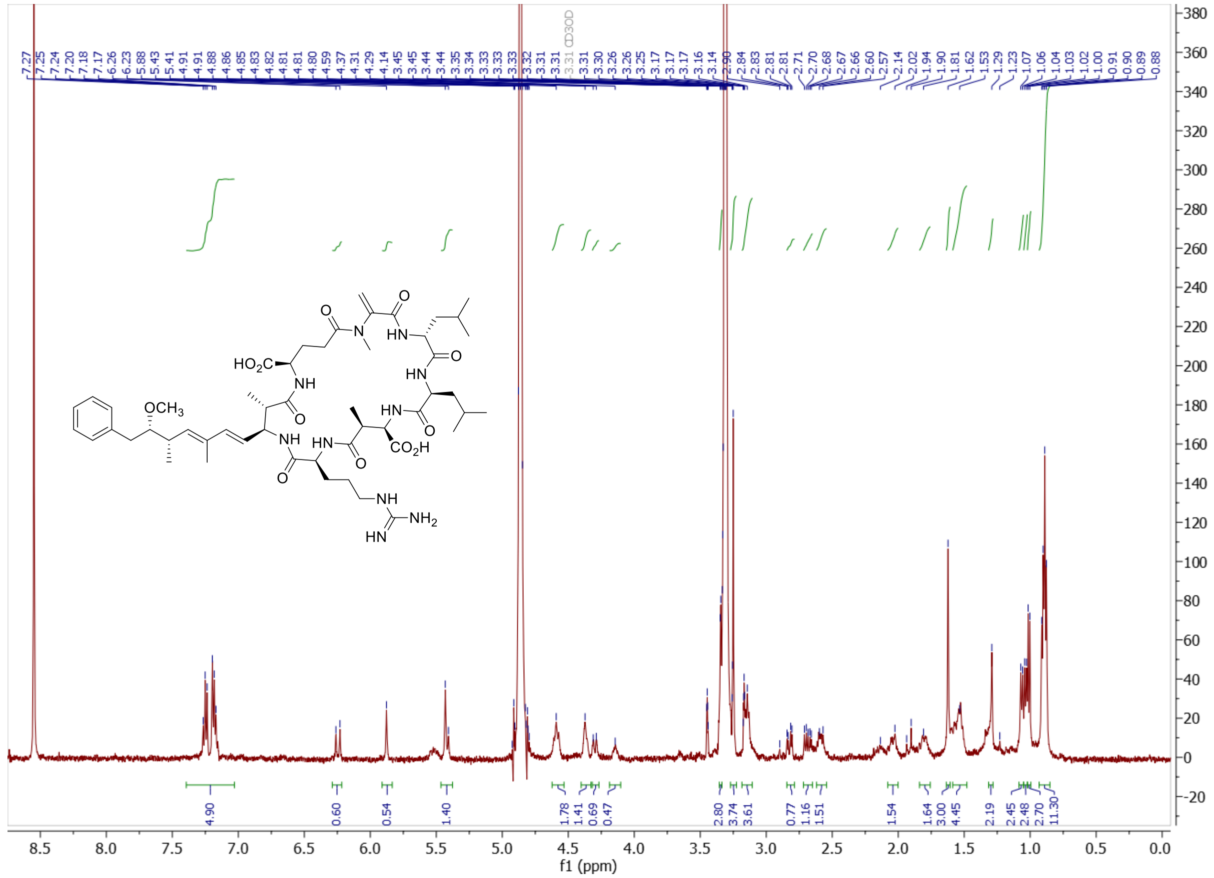

Figure S10. ^{1}H NMR (500 MHz, MeOH-d_4) of [Leu[1]]MC-LR.

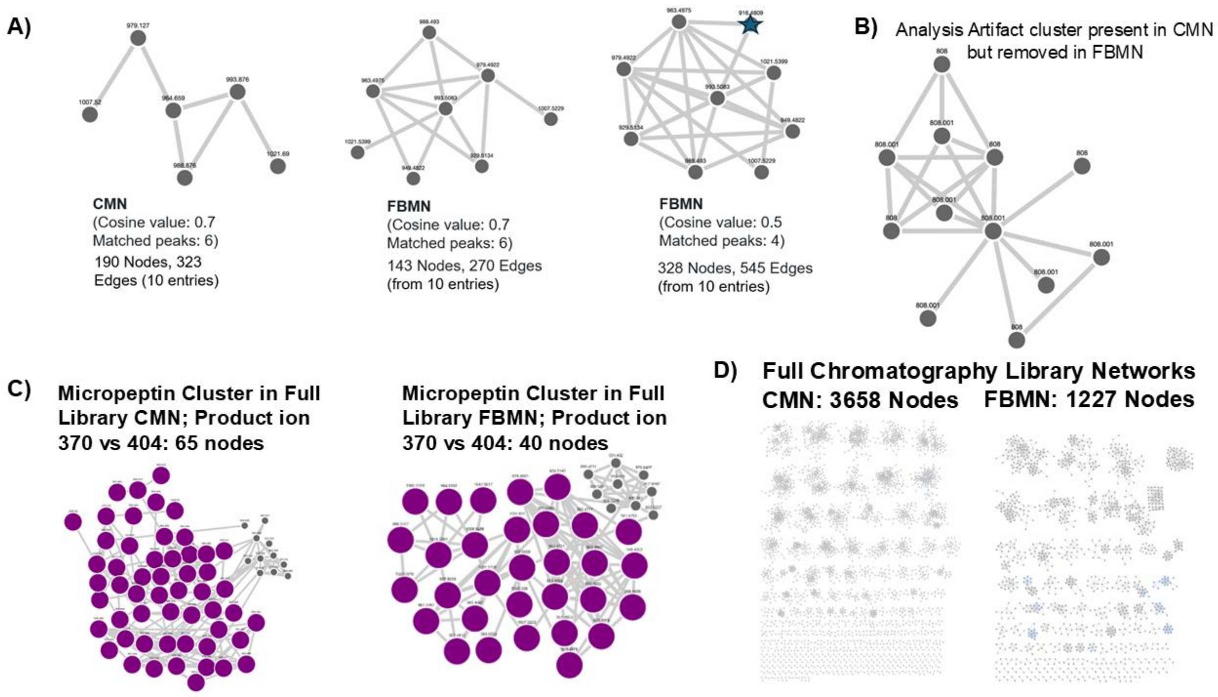

Figure S11. CMN vs. FBMN. (A) The micropeptin cluster (from the initial subset of chromatography fractions) under different workflows and different parameter settings. (B) Artifact cluster in classical molecular networking that was removed in FBMN. (C) CMN vs. FBMN for the micropeptin cluster in the full chromatography fraction network with product ion searching. (D) CMN vs. FBMN for the full chromatography fraction library.

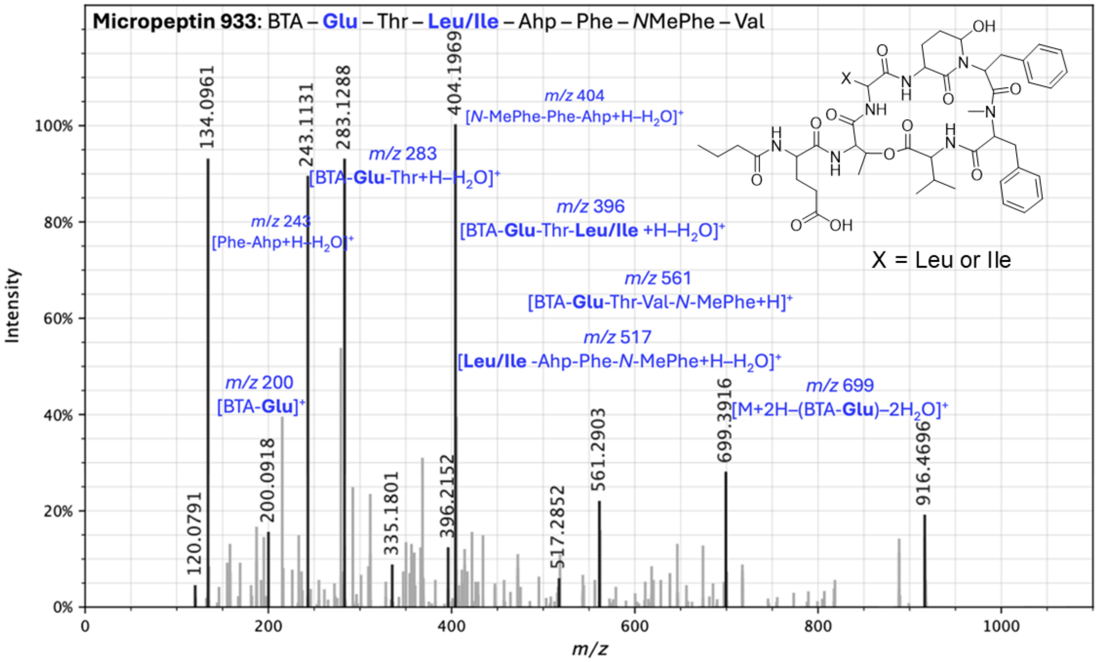

Figure S12. Annotated MS/MS spectrum of micropeptin 933.

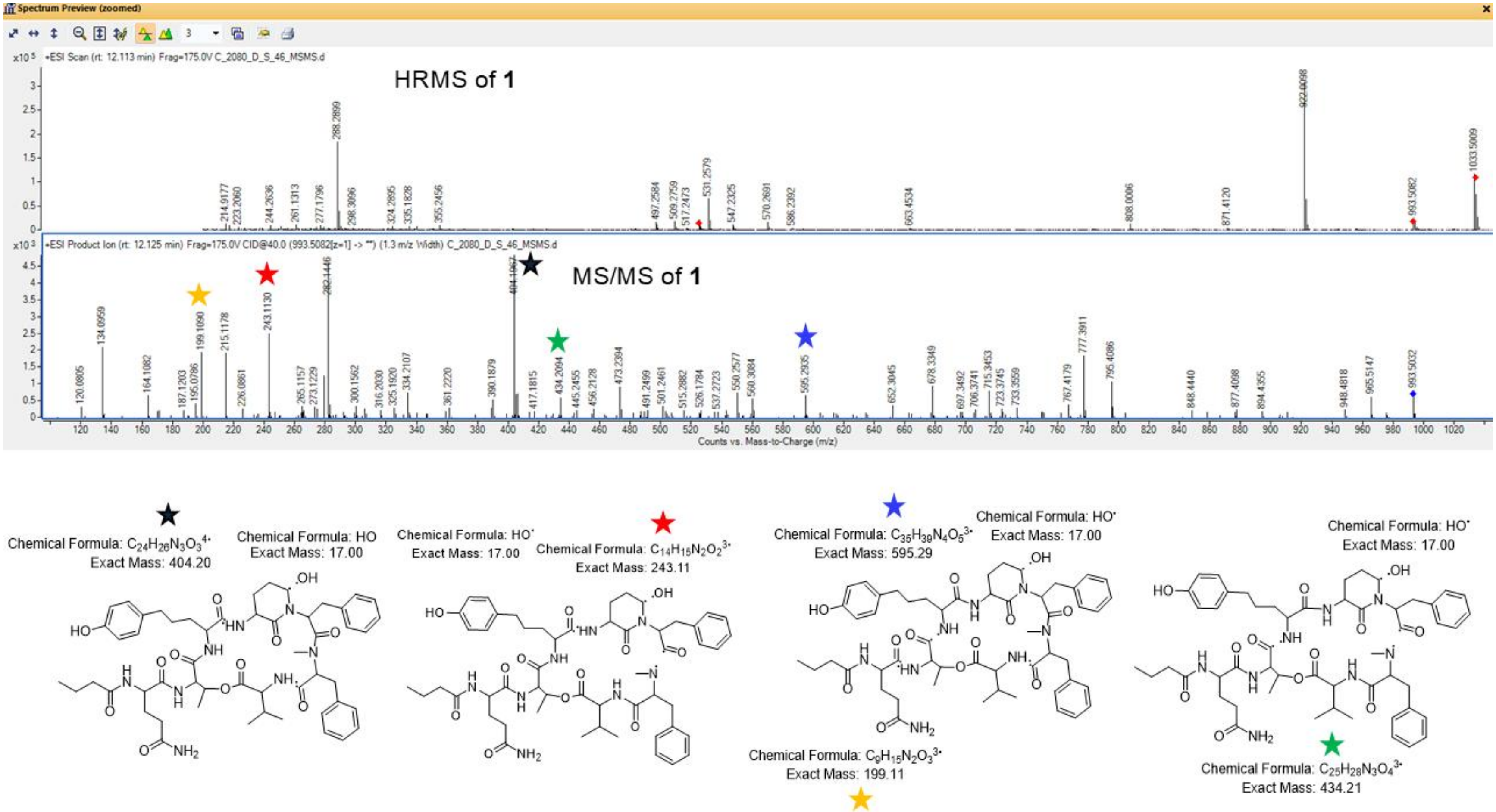

Figure S13. Mass spectrometry data of **1**. Top panel: HRMS of compound **1** *m/z* 1033.5009 $[M+Na]^+$. Middle panel: MS/MS of **1** with key fragmentation ions noted with stars, which correspond to the putative fragmentations illustrated in the bottom panel.

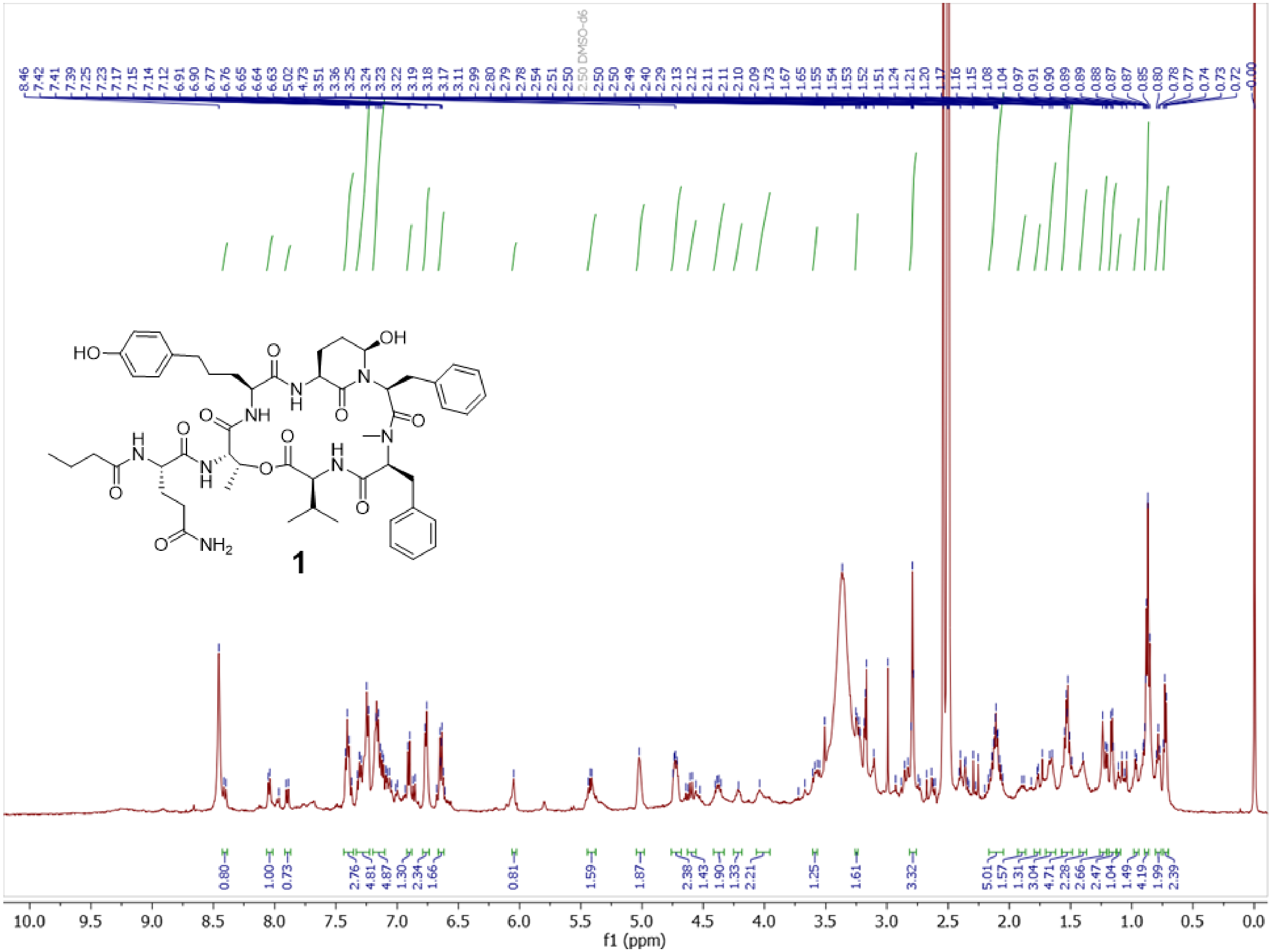

Figure S14. ^{1}H NMR (500 MHz, DMSO-d_6) of micropeptin 1010 (**1**).

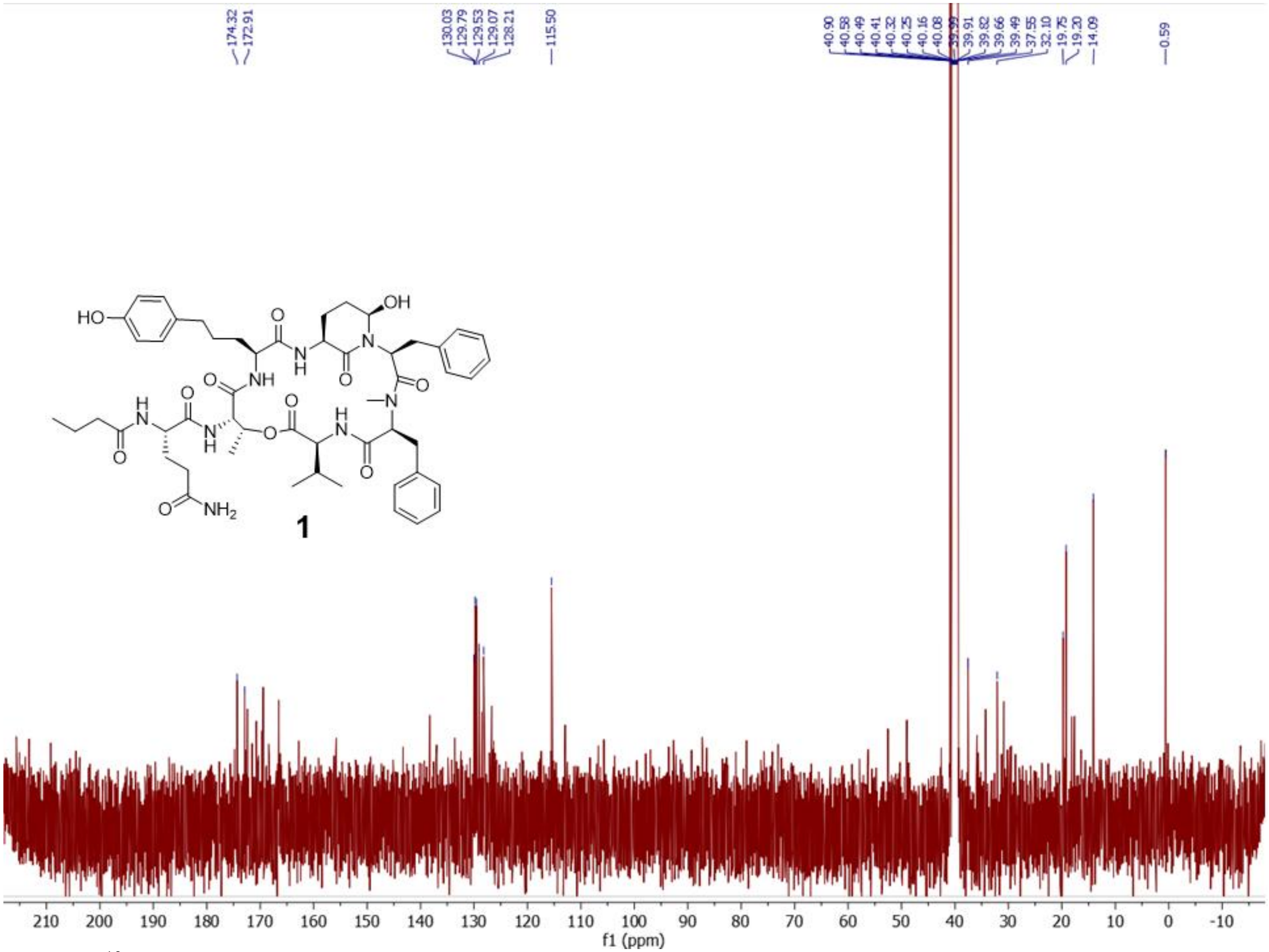

Figure S15. ^{13}C NMR (125 MHz, DMSO-d_6) of micropeptin 1010 (**1**).

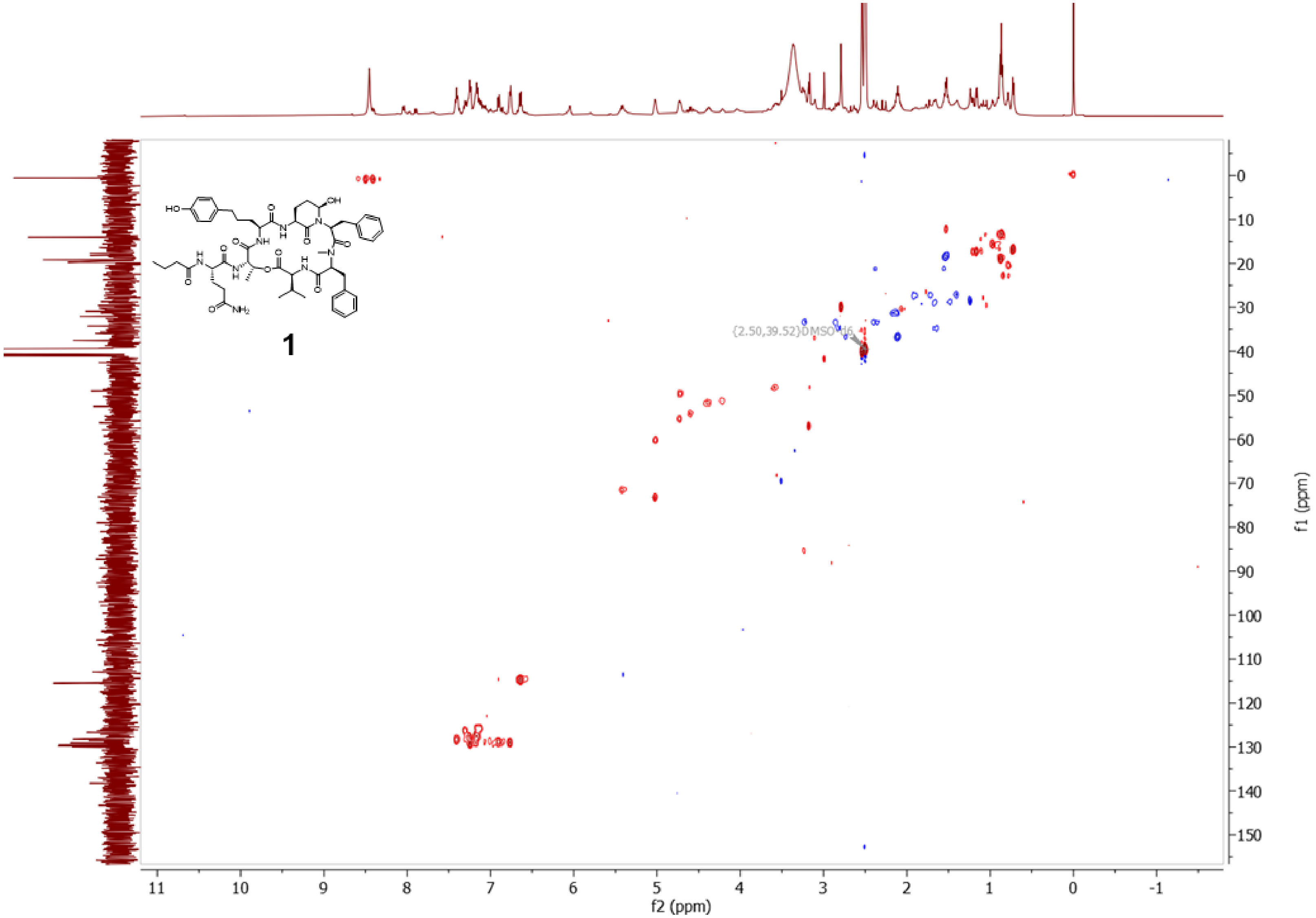

Figure S16. Multiplicity-edited HSQC of micropeptin 1010 (**1**).

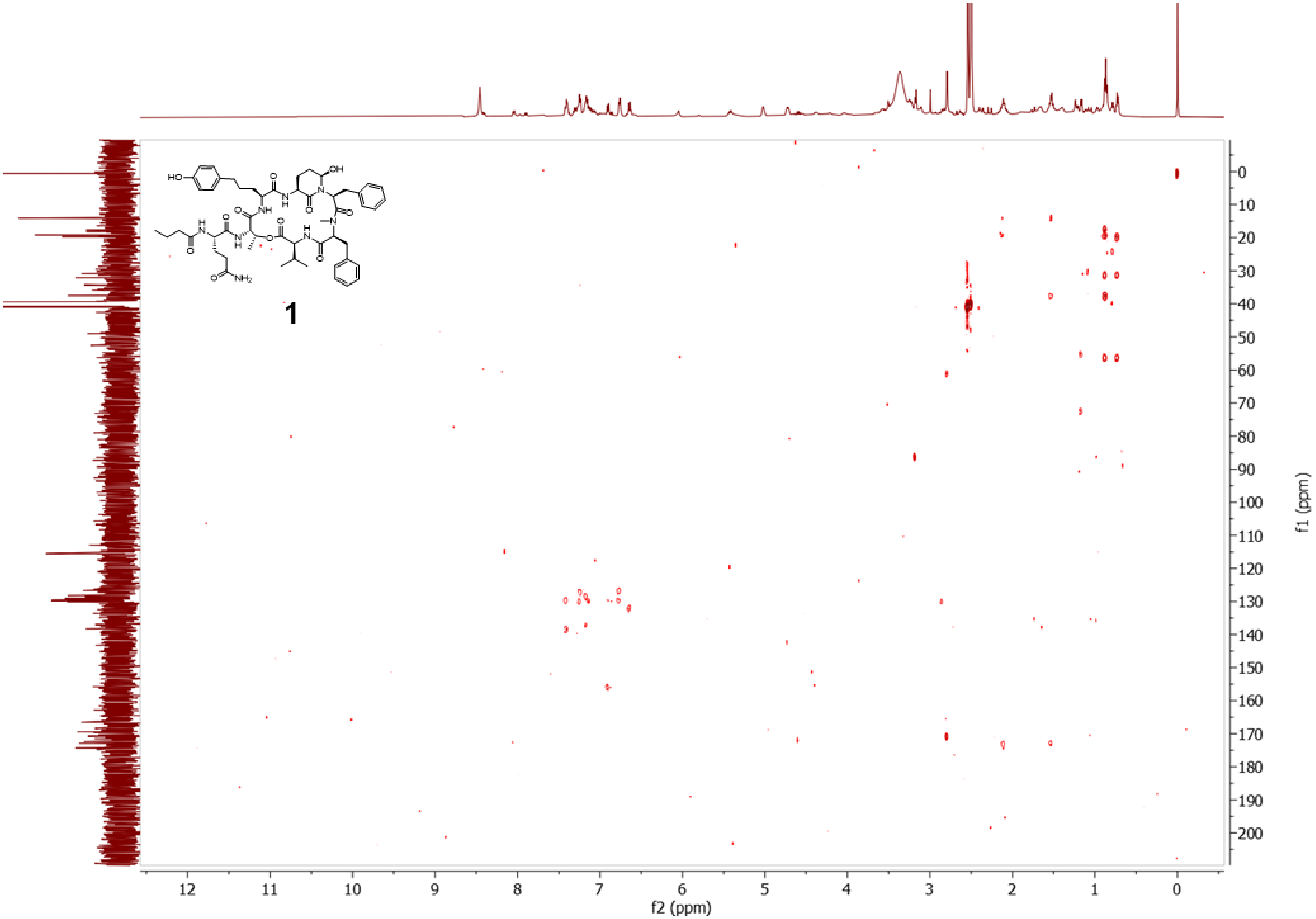

Figure S17. HMBC of micropeptin 1010 (**1**).

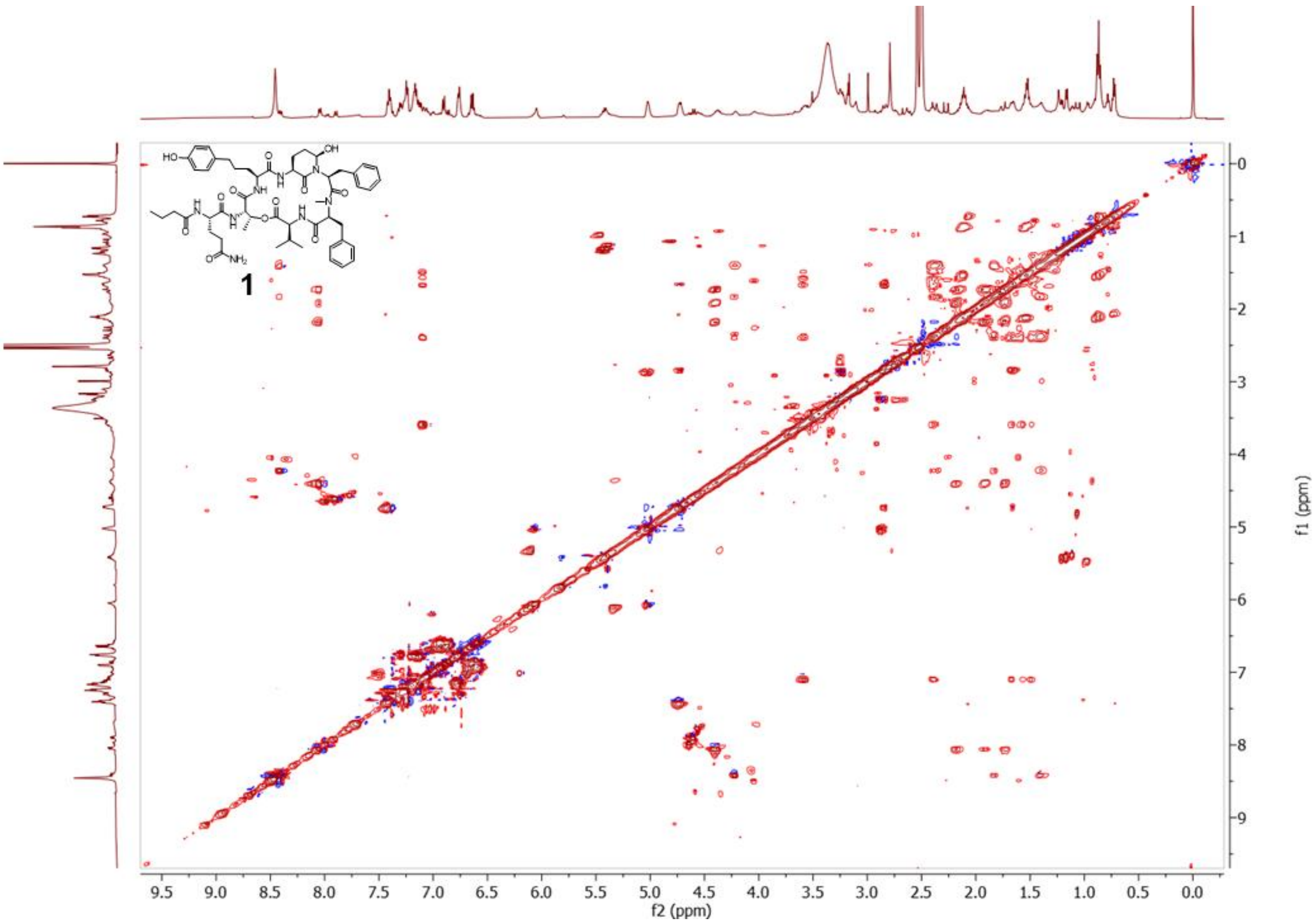

Figure S18. TOCSY of micropeptin 1010 (**1**).

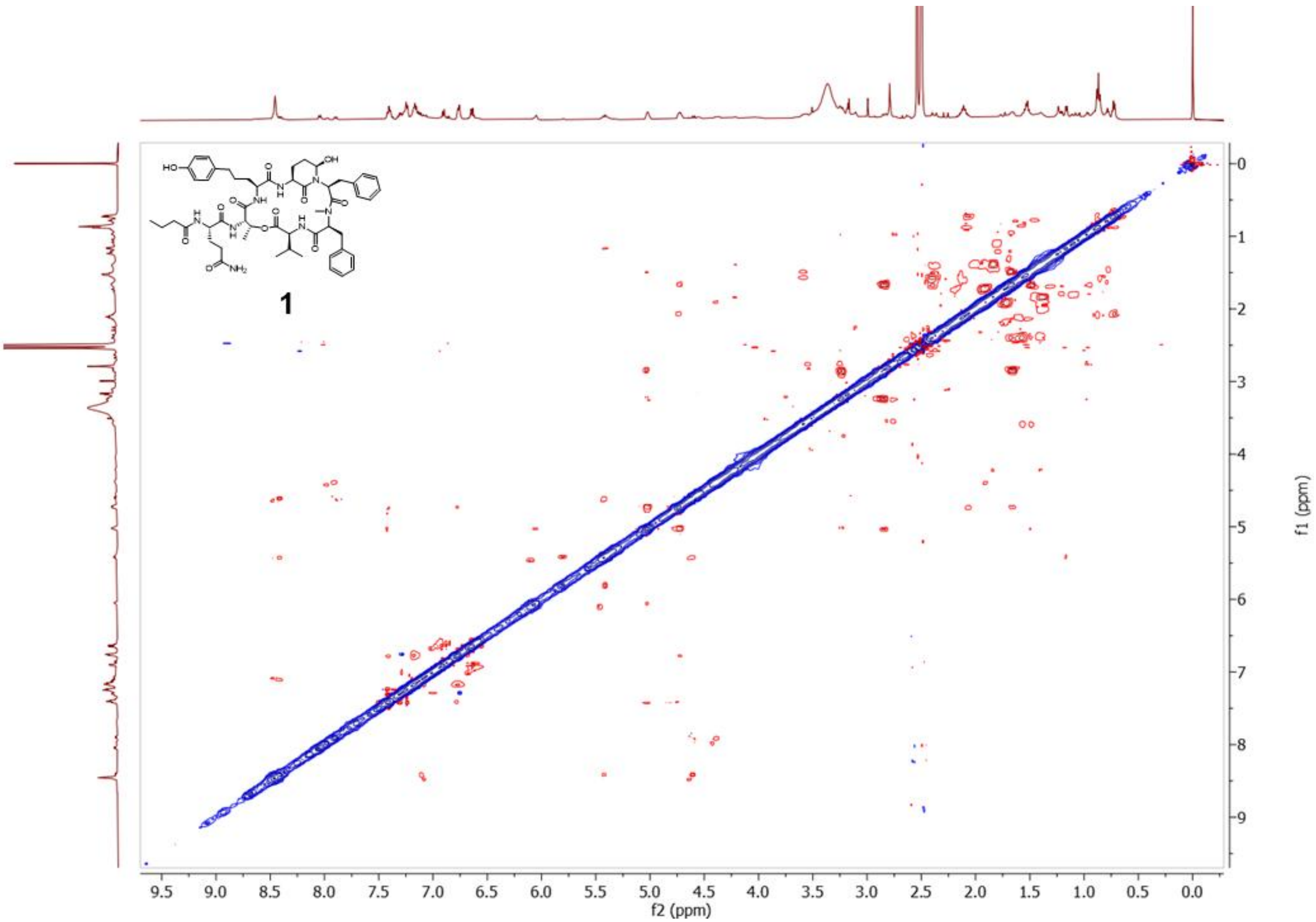

Figure S19. NOESY of micropeptin 1010 (**1**).

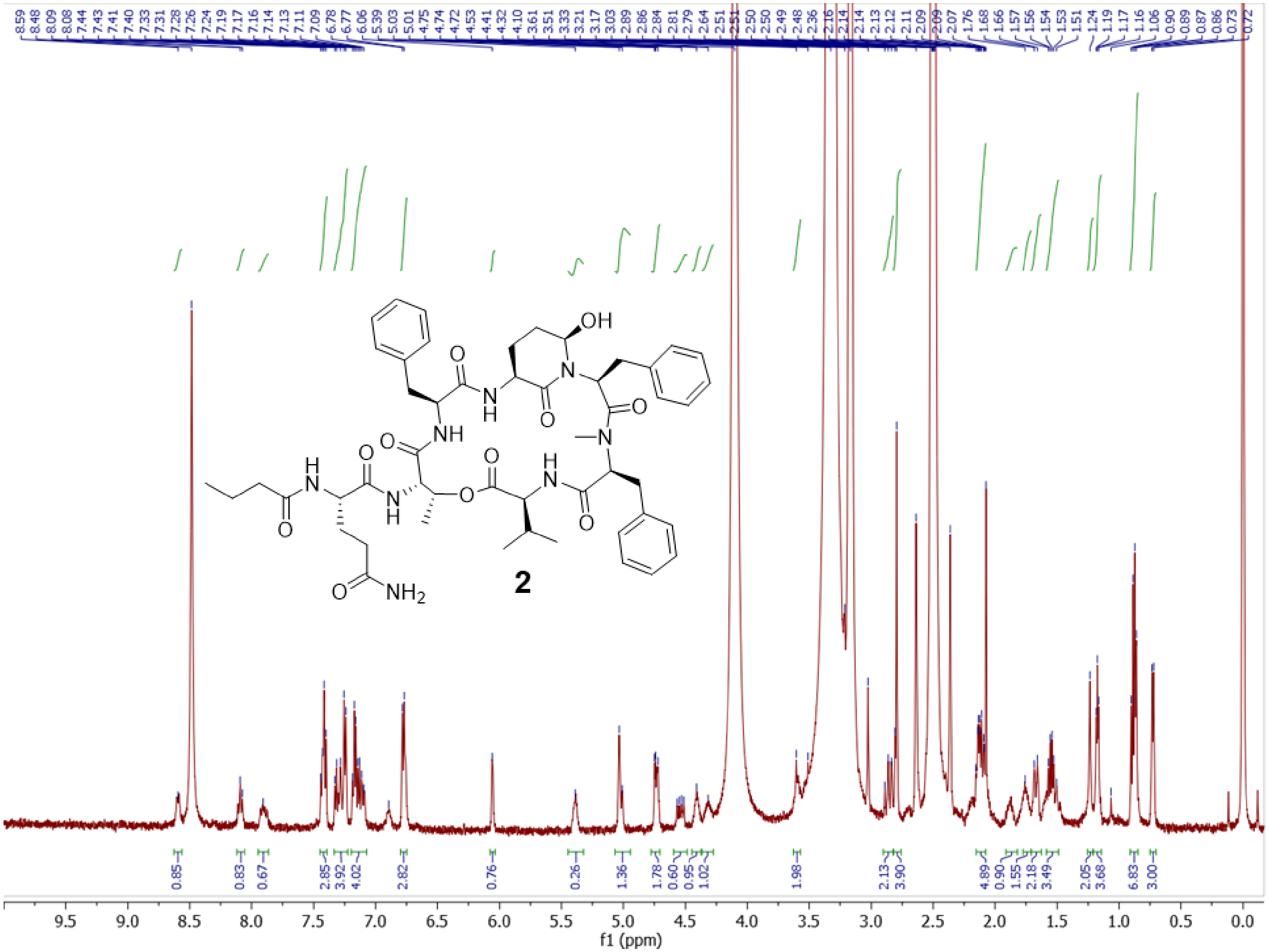

Figure S20. ^{1}H NMR (500 MHz, DMSO-d_6) of micropeptin 966 (D-Gln) (**2**).

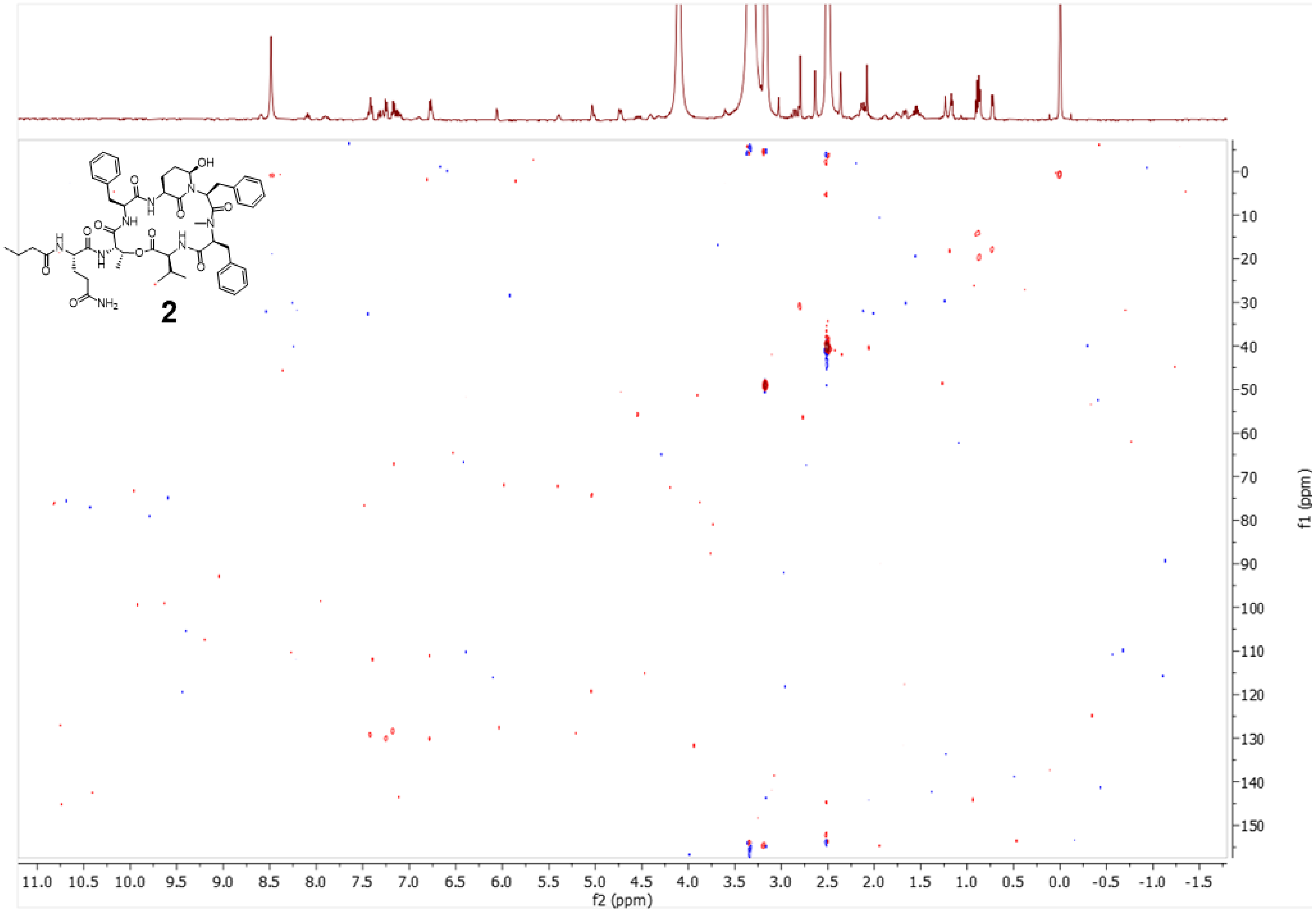

Figure S21. Multiplicity-edited HSQC of micropeptin 966 (D-Gln) (**2**).

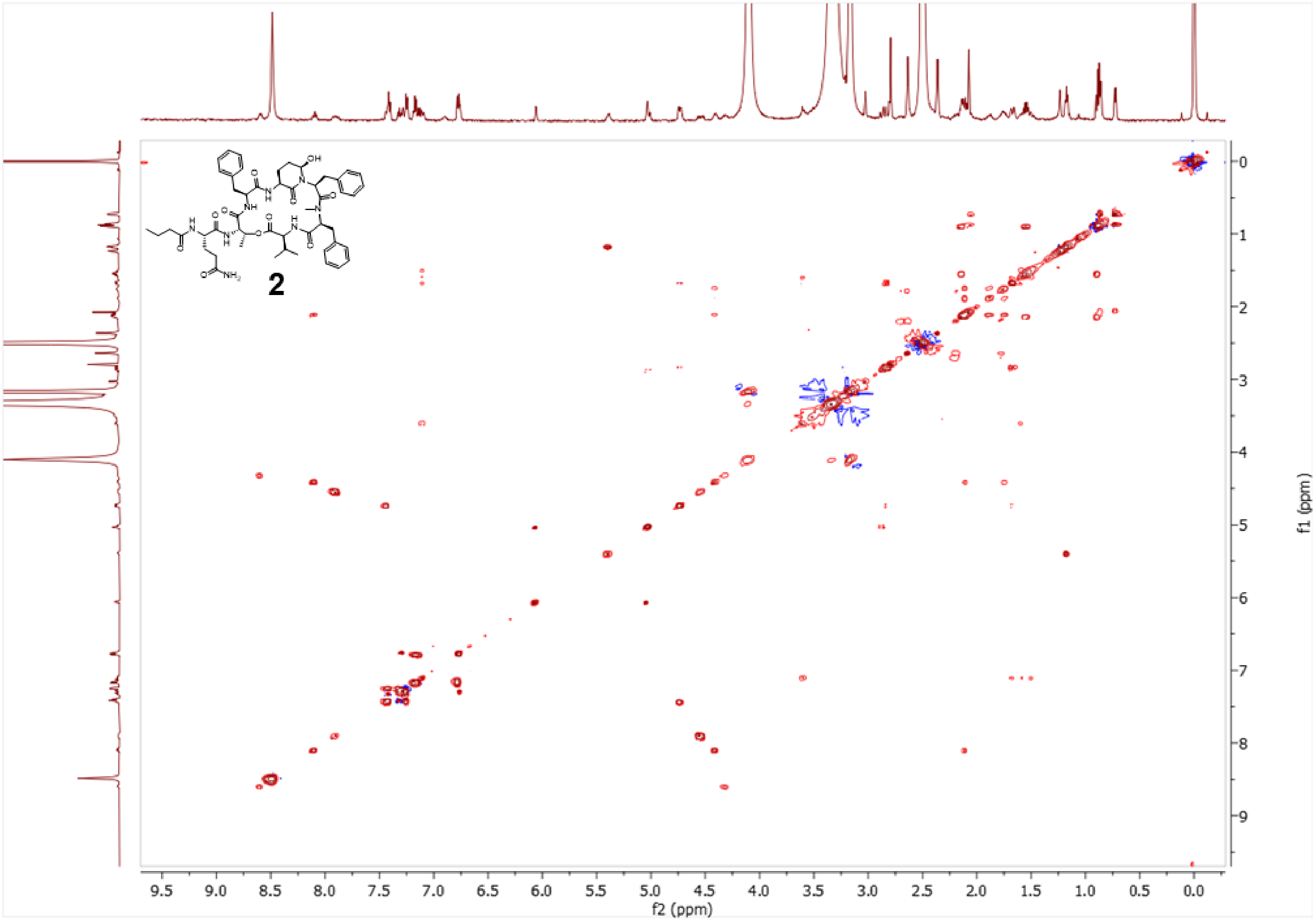

Figure S22. TOCSY of micropeptin 966 (D-Gln) (**2**).

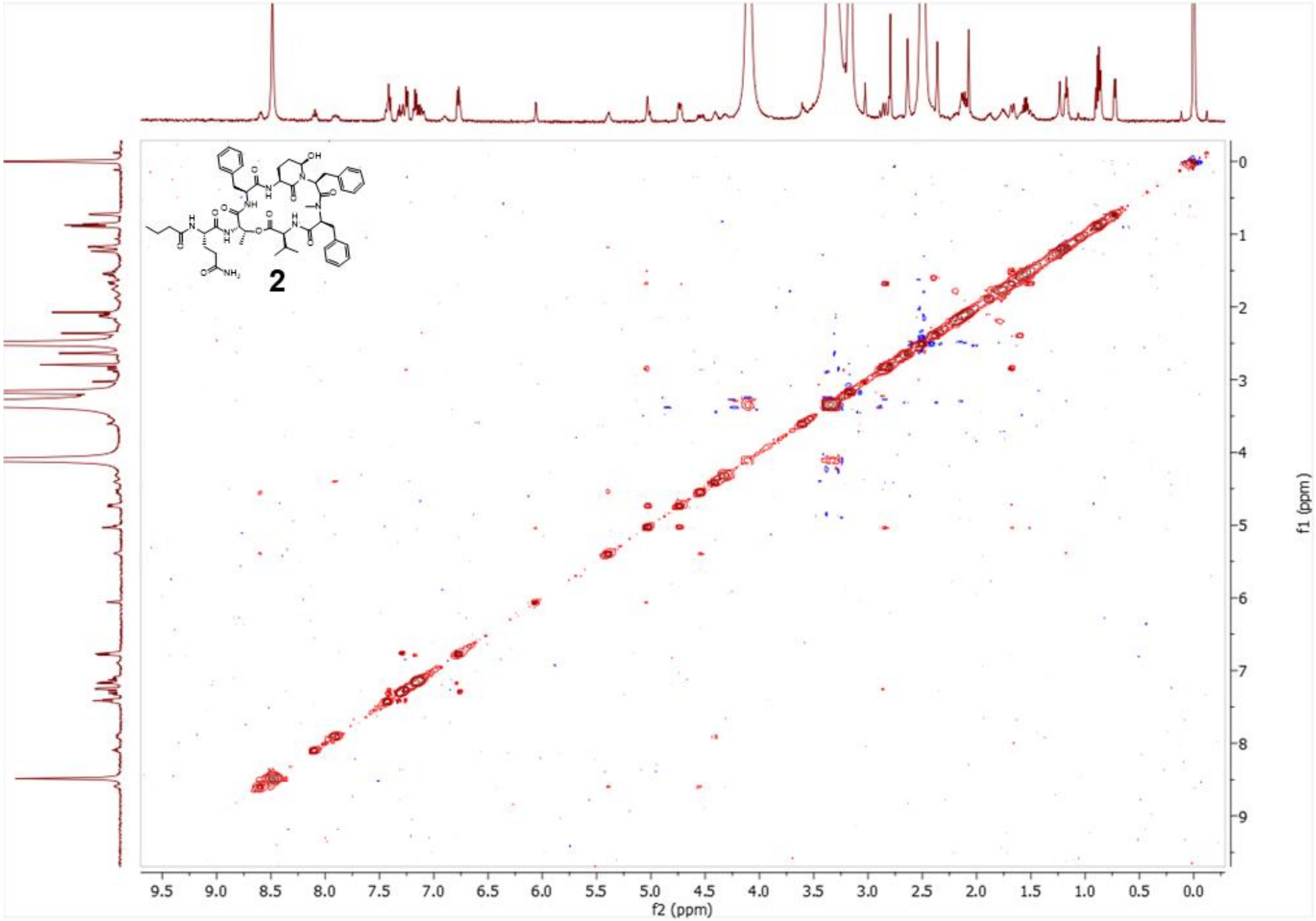

Figure S23. NOESY of micropeptin 966 (D-Gln) (**2**).

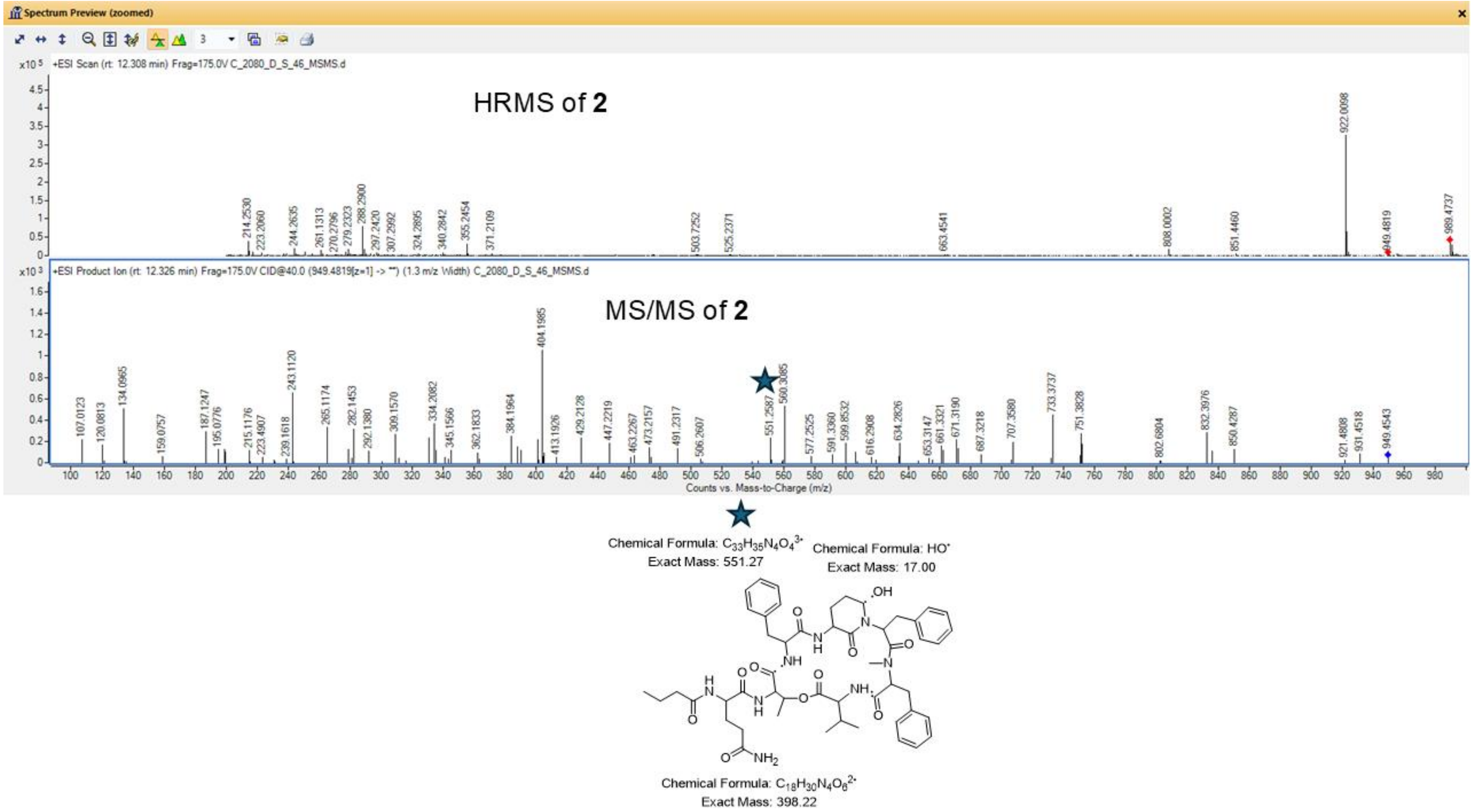

Figure S24. Mass spectrometry data of **2.** Top panel: HRMS of compound **2** *m/z* 989.4737 $[M+Na]^+$. Middle panel: MS/MS of **2** with a key fragmentation ion noted with a star, which corresponds to the putative fragmentation illustrated in the bottom panel.

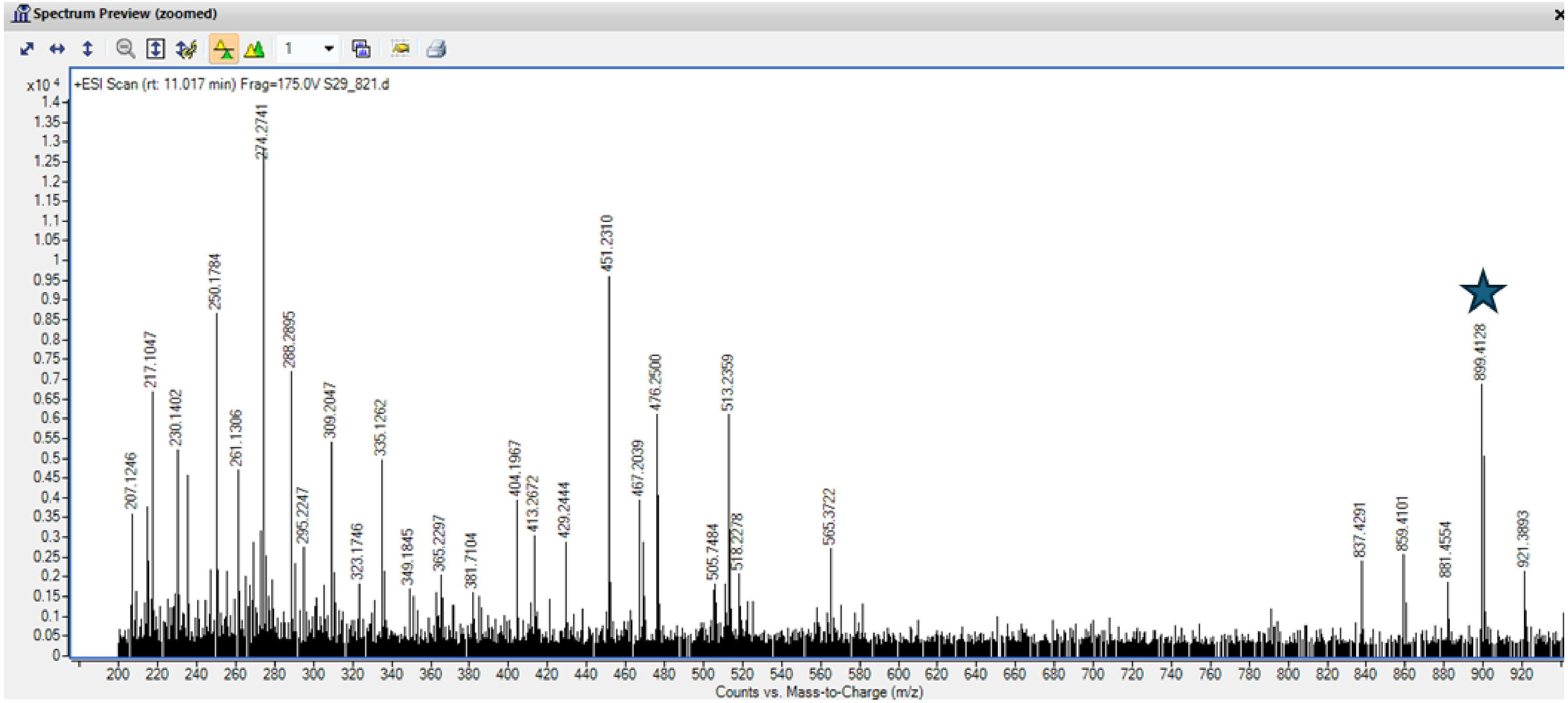

Figure S25. HRMS of ferintoic acid C *m/z* 899.4128 $[M+H]^+$.

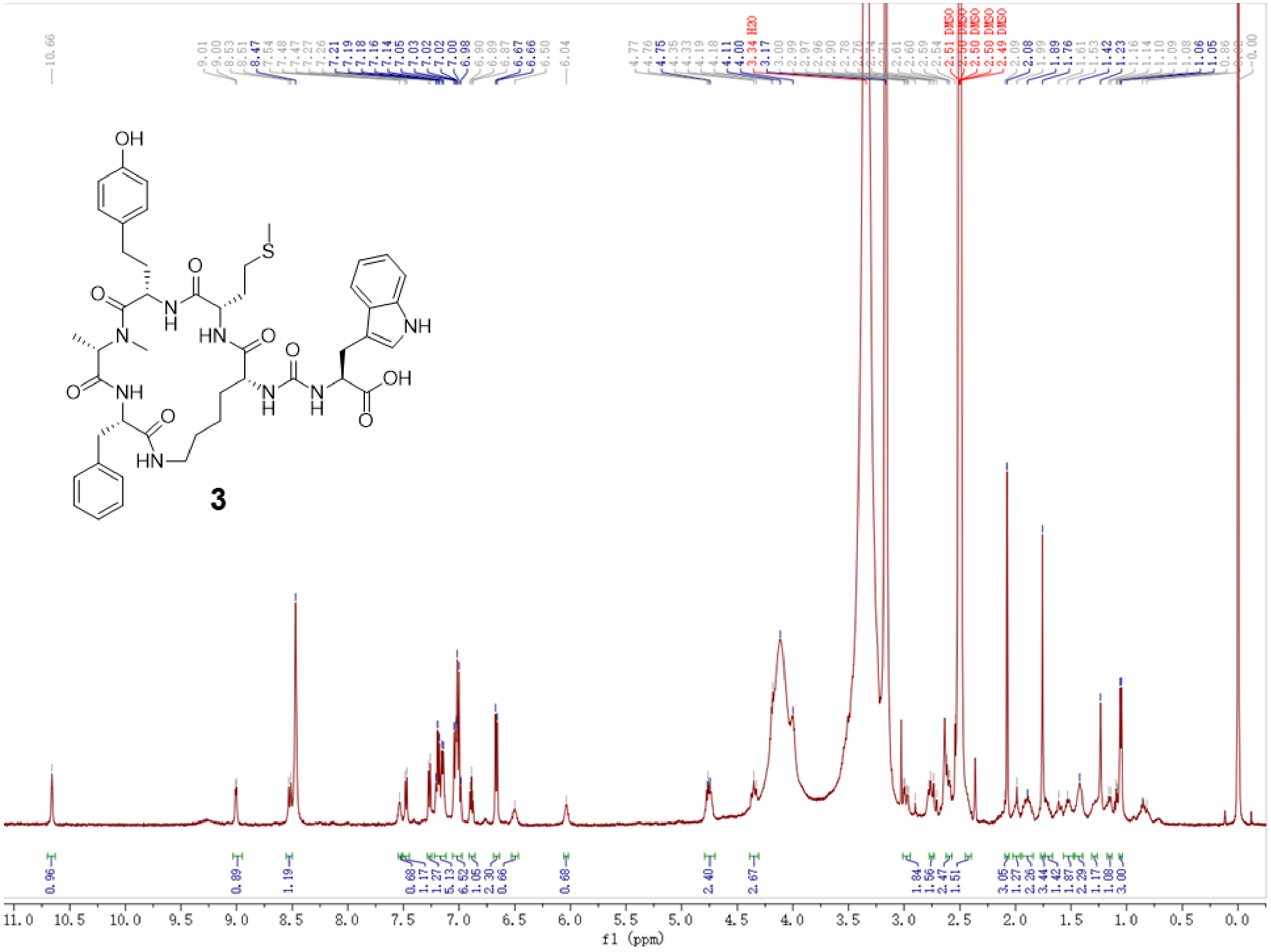

Figure S26. ^{1}H NMR (500 MHz, DMSO-d_6) of ferintoic acid C (**3**).

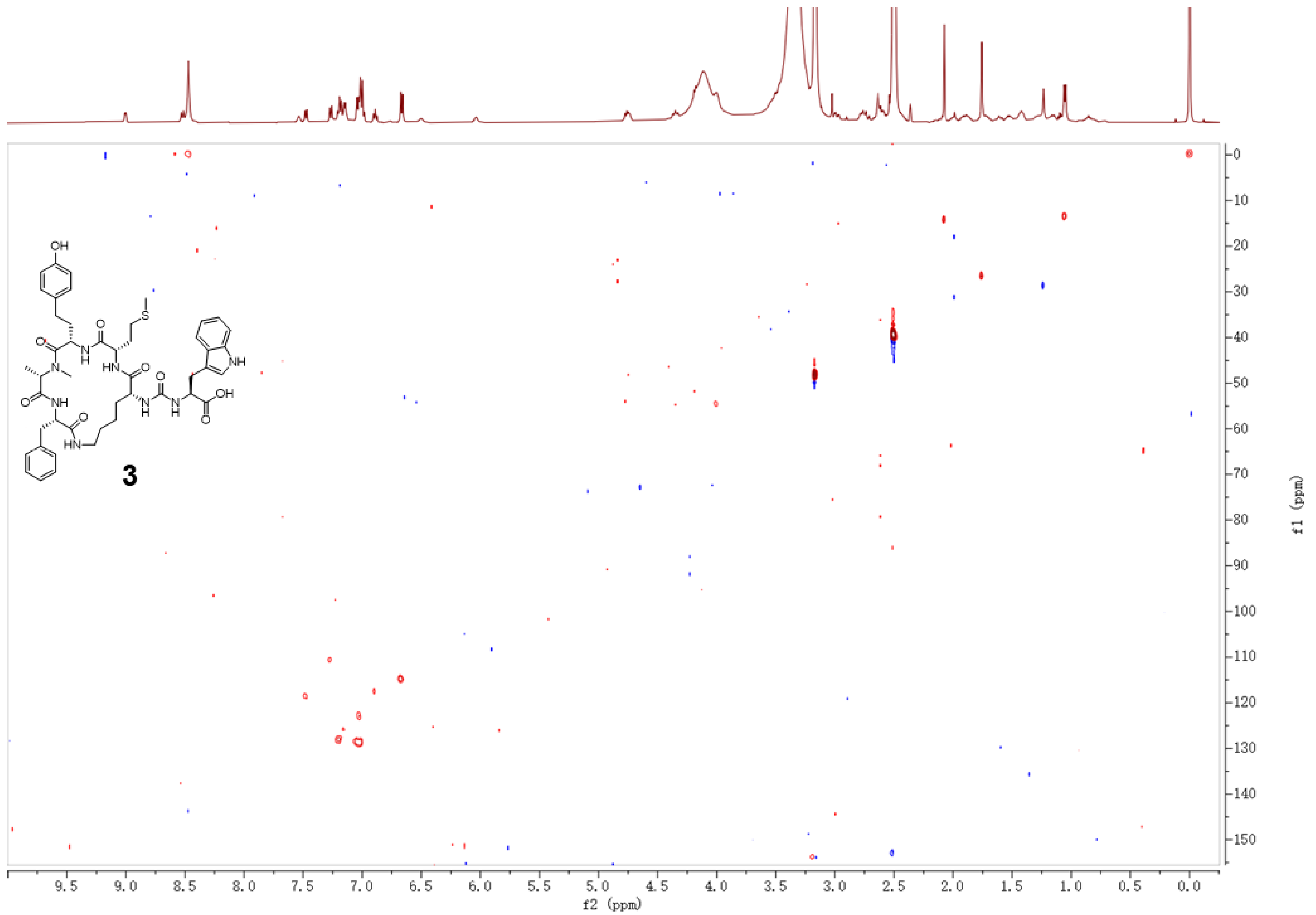

Figure S27. Multiplicity-edited HSQC of ferintoic acid C (**3**).

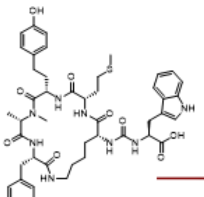

3

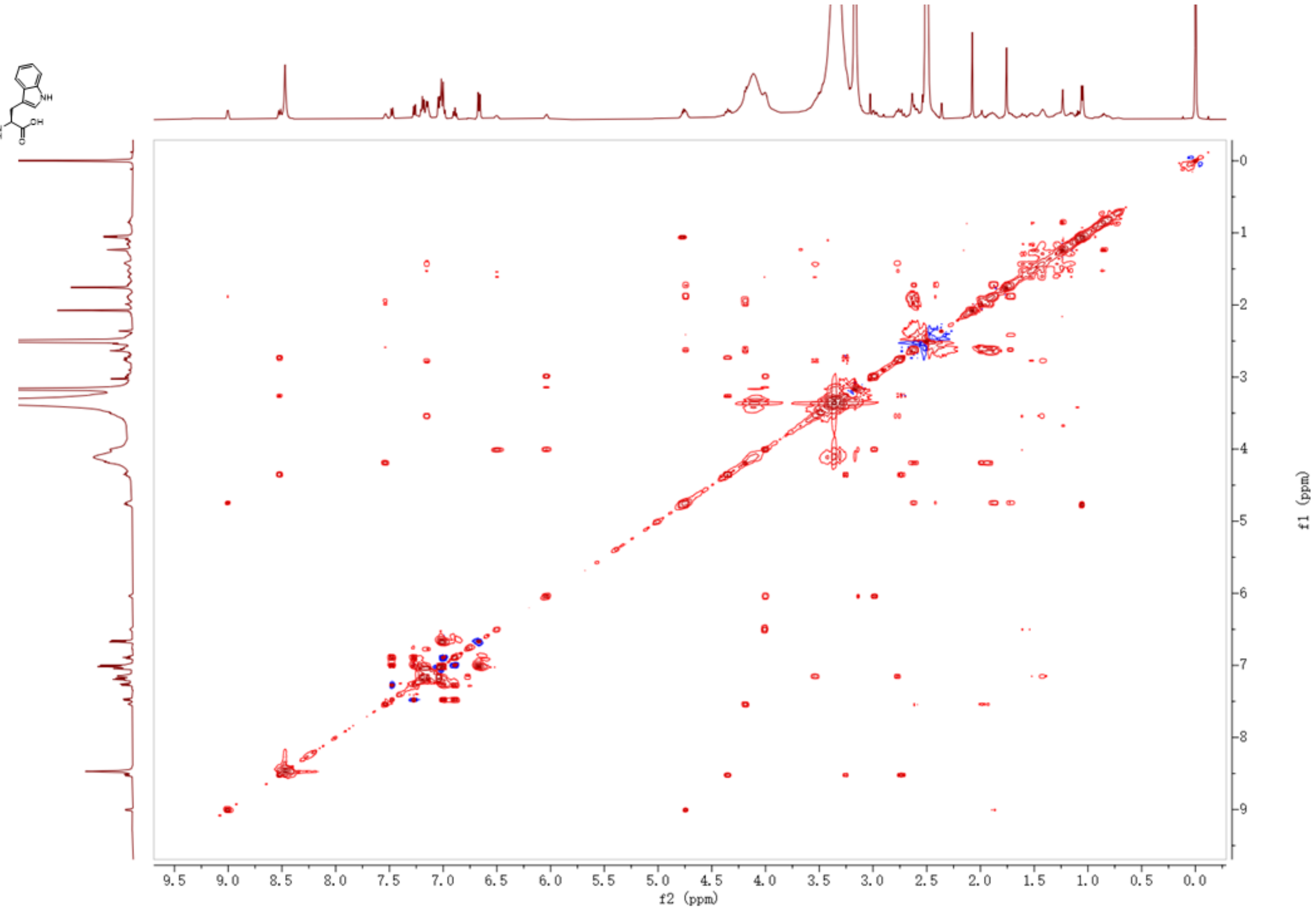

Figure S28. TOCSY of ferintoic acid C (**3**).

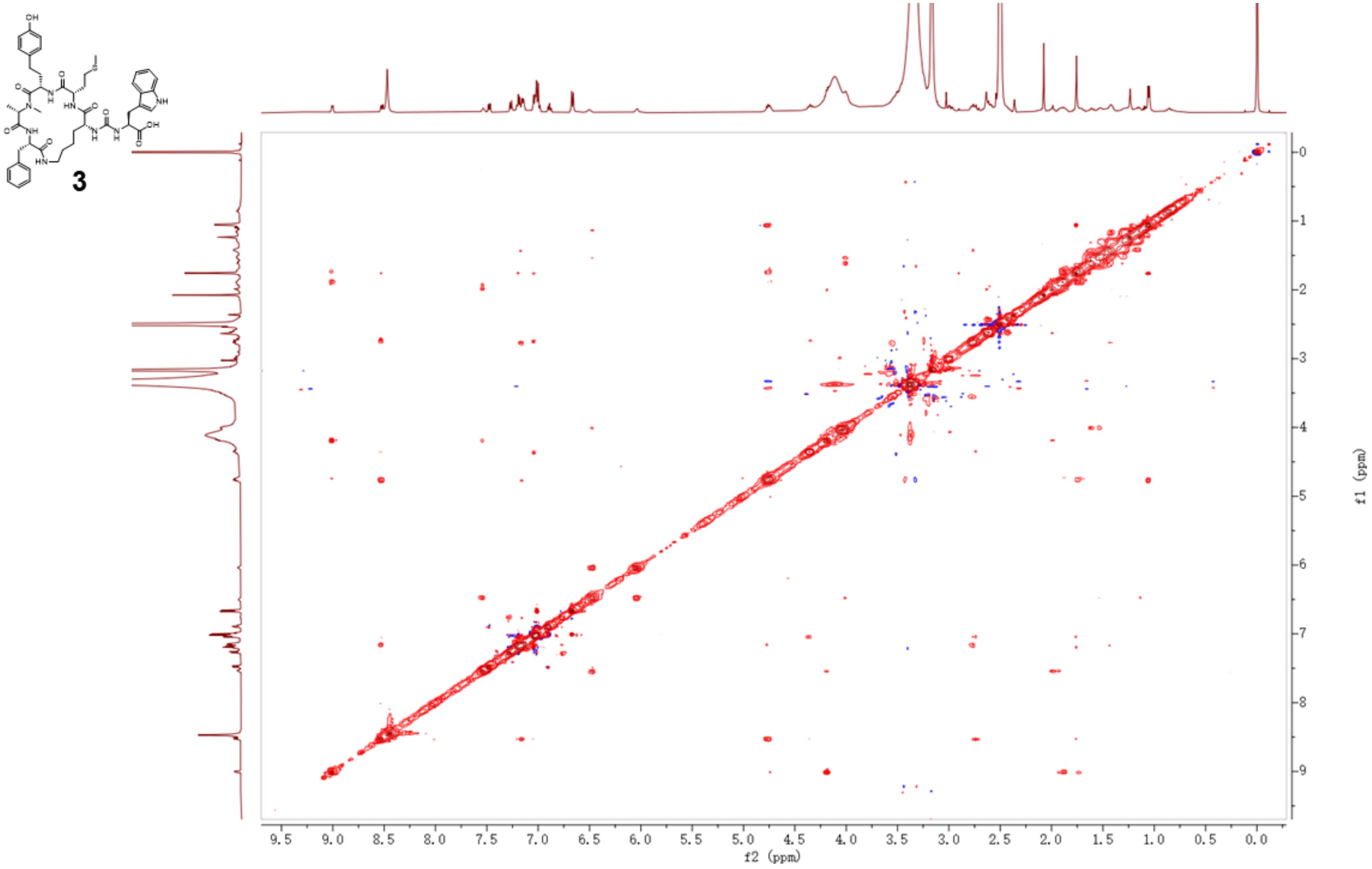

Figure S29. NOESY of ferintoic acid C (**3**).

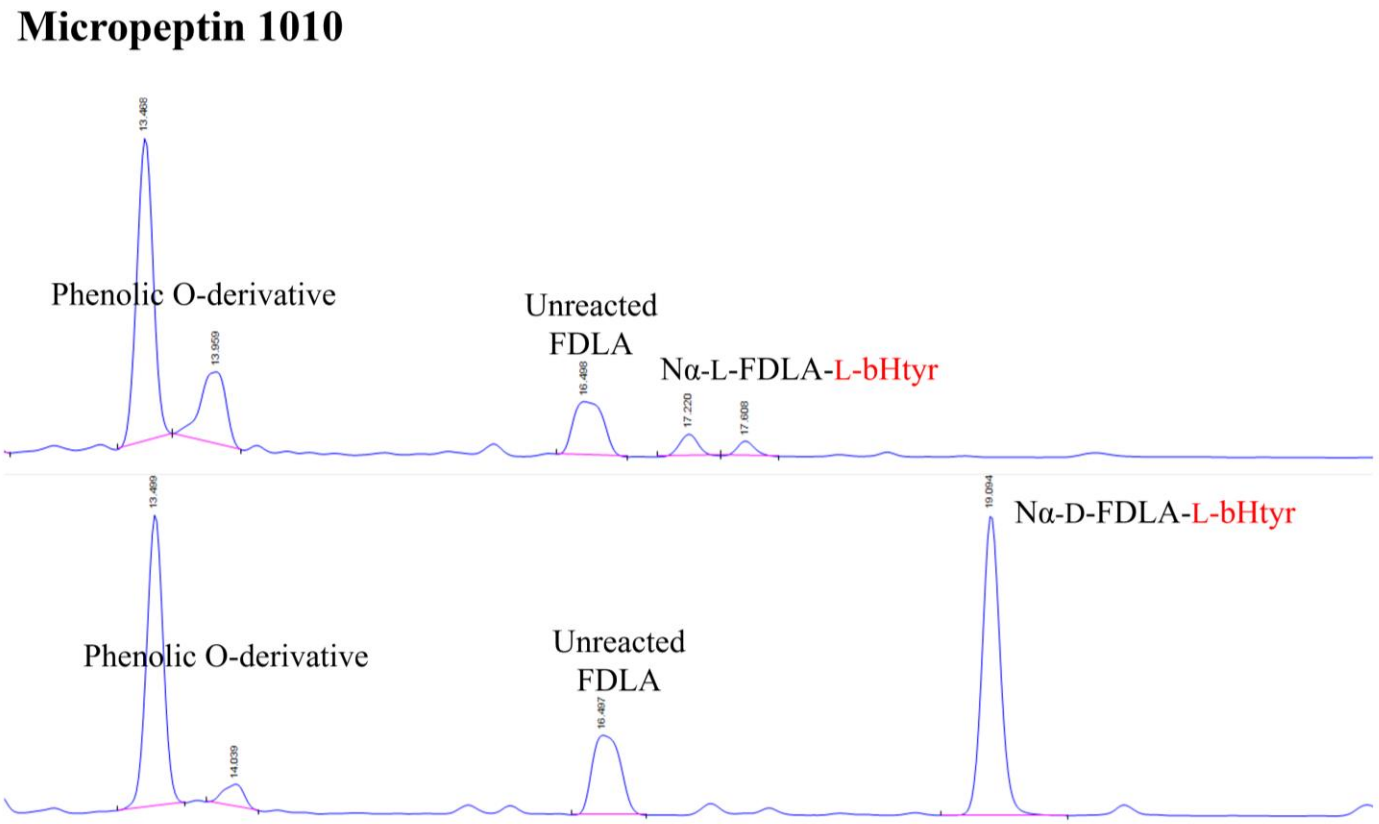

Figure S30. LC-MS analysis of the hydrolysate of **1** reacted with L-FDLA (top panel) and D-FDLA (bottom panel) to determine the configuration of the bHtyr in **1**.

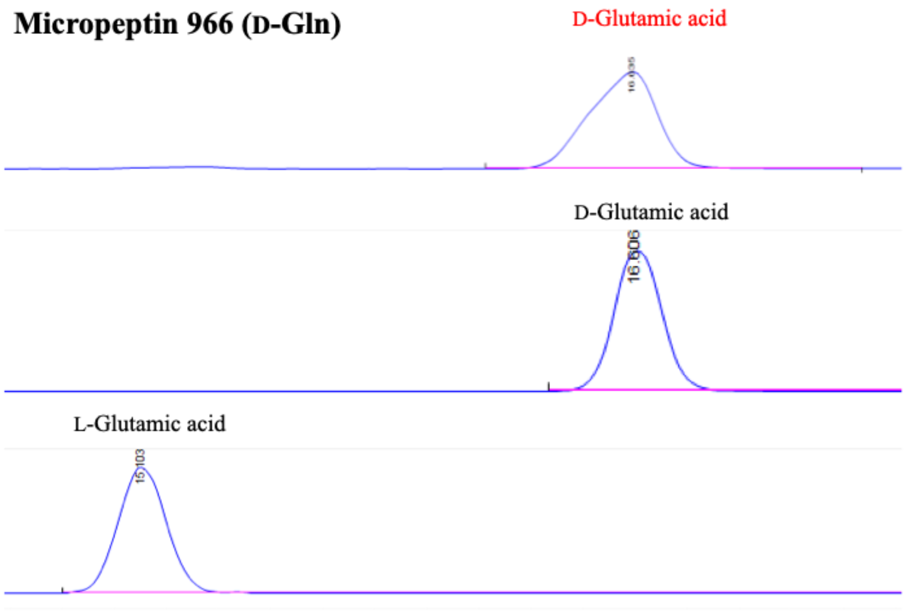

Figure S31. LC-MS analysis of the hydrolysate of **2** (top panel) and L- and D-Glutamic acid (middle and bottom panel, respectively) derivatized with L-FDVA.

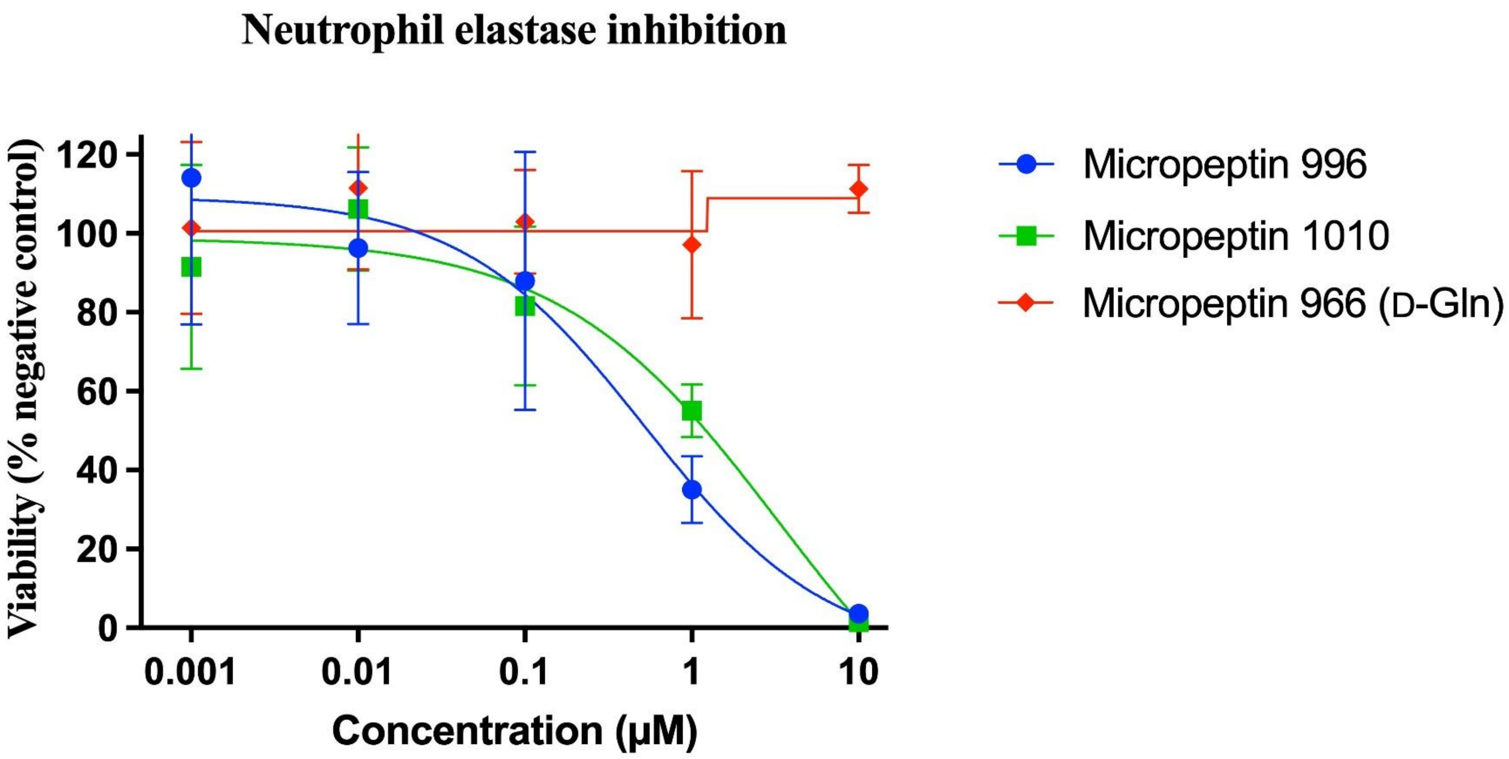

Figure S32. Activity of micropeptin 996 (L-Gln), micropeptin 1010 (**1**), and micropeptin 996 (D-Gln) (**2**) against human neutrophil elastase.

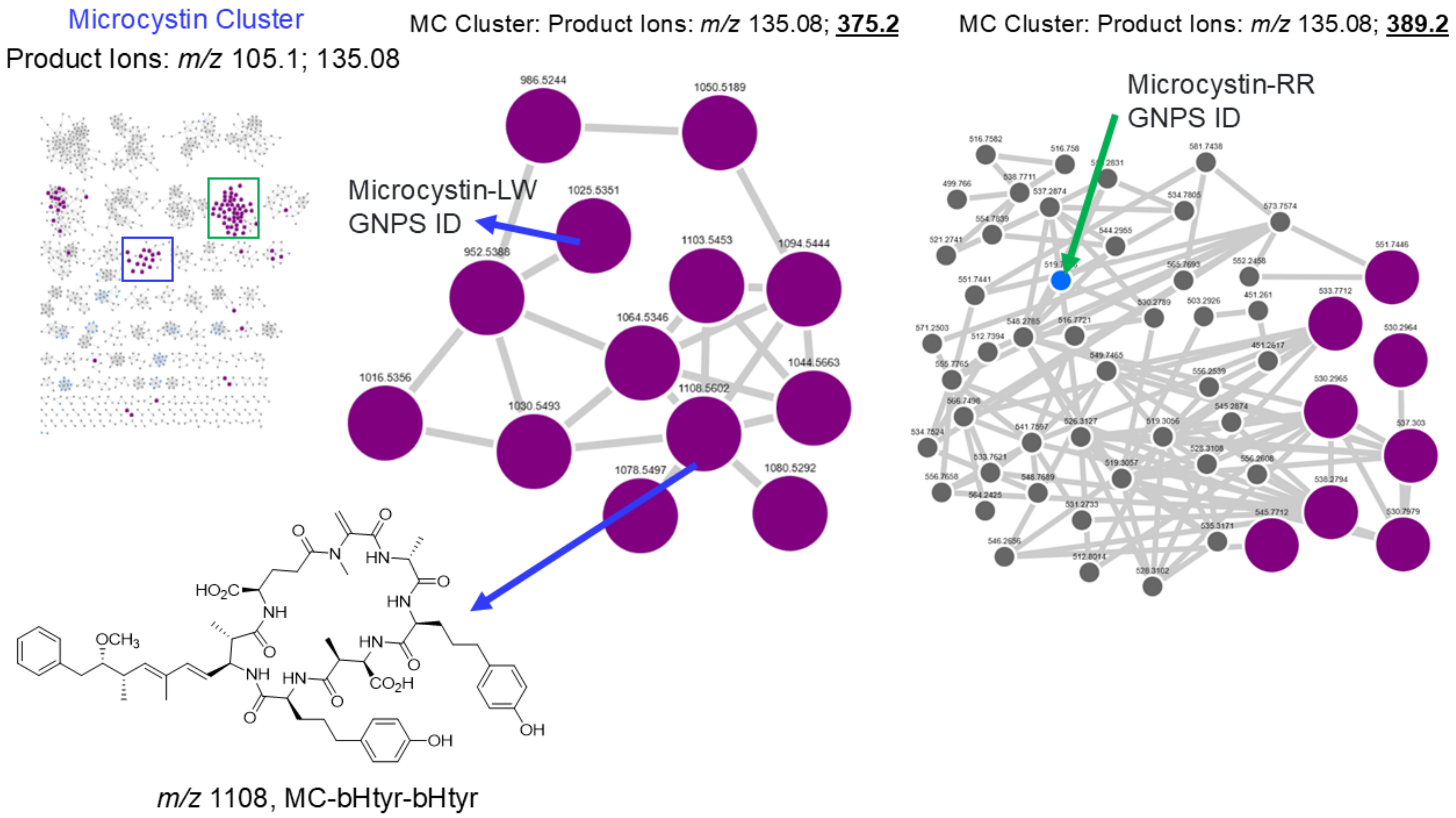

Figure S33. Microcystin clusters in MS/MS molecular network subjected to two different product ion searches: *m/z* 135.08 and *m/z* 375.2 and then *m/z* 135.08 and *m/z* 389.02 to illustrate microcystins with likely [Glu(OCH_3)^6 modifications, which is also supported by the annotation of library microcystins shown (*m/z* 1108).

Anabaenopeptin/Ferintoic acid cluster: Product ions: *m/z* 114.055; *m/z* 405.2

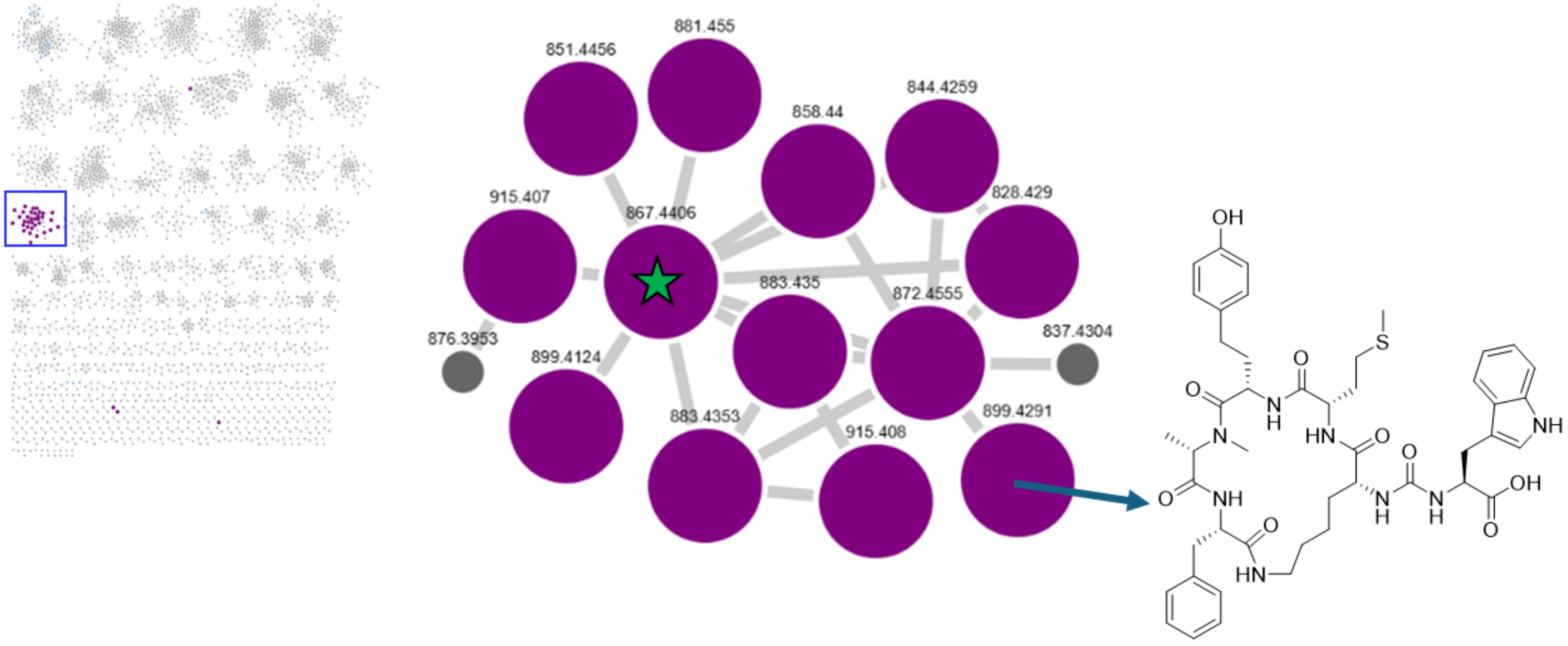

m/z 899 Ferintoic Acid C (**3**)

Figure S34. MS/MS cluster of anabaenopeptins/ferintoic acids annotated via product ion searching. Ferintoic acid A (green star) and ferintoic acid C (**3**) were validated using our standard library.

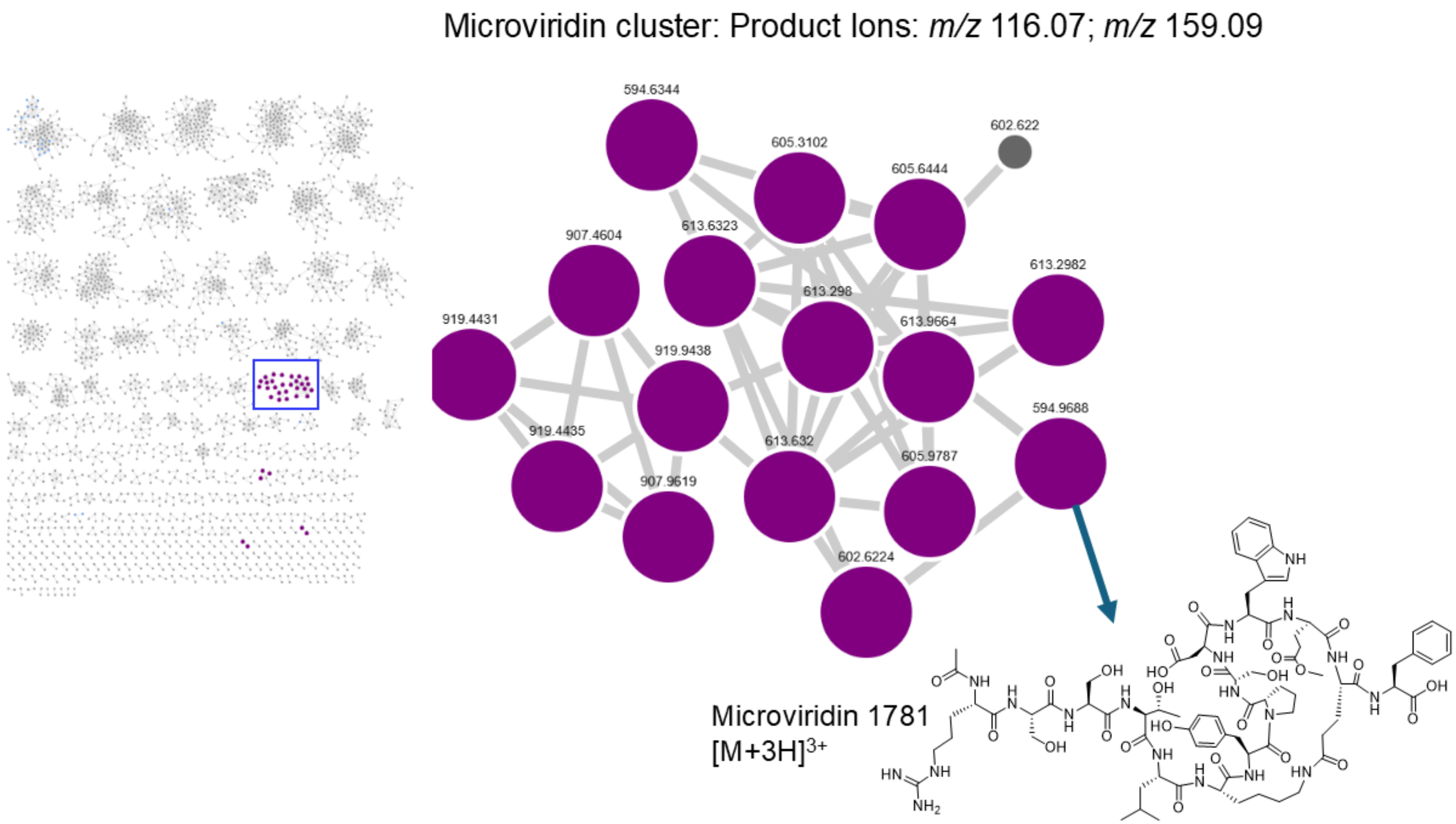

Figure S35. Microviridin cluster annotated using product ion searching in MS/MS network. Standard compound microviridin 1781 was used for validation $[M+3H]^{3+}$ ion *m/z* 594.

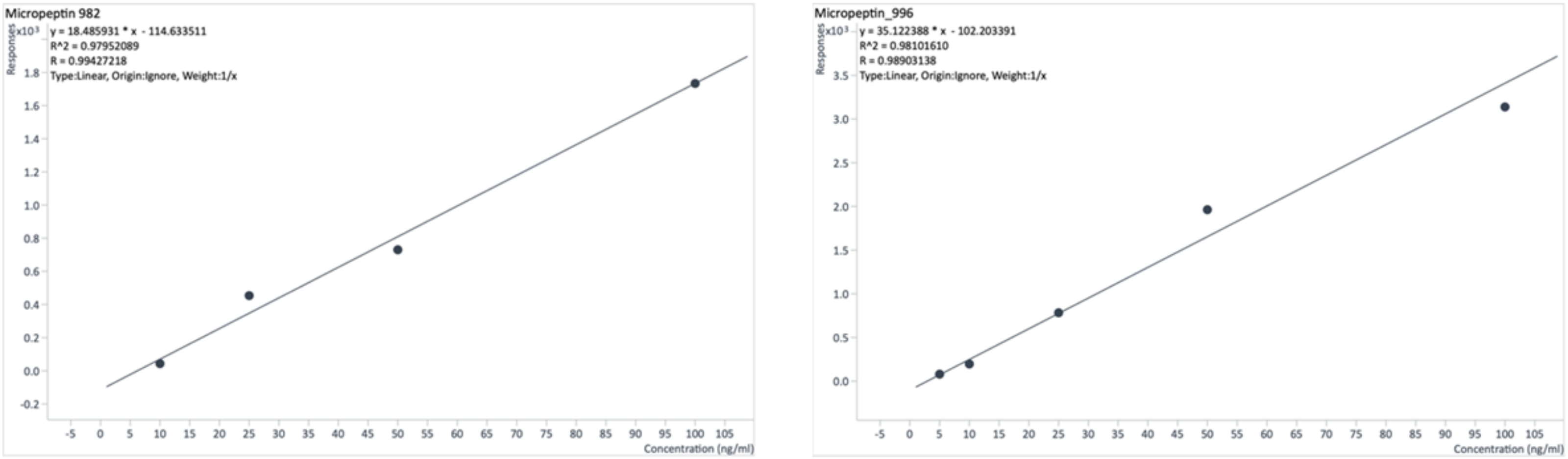

Figure S36. Calibration curves of micropeptin 982 and 996.

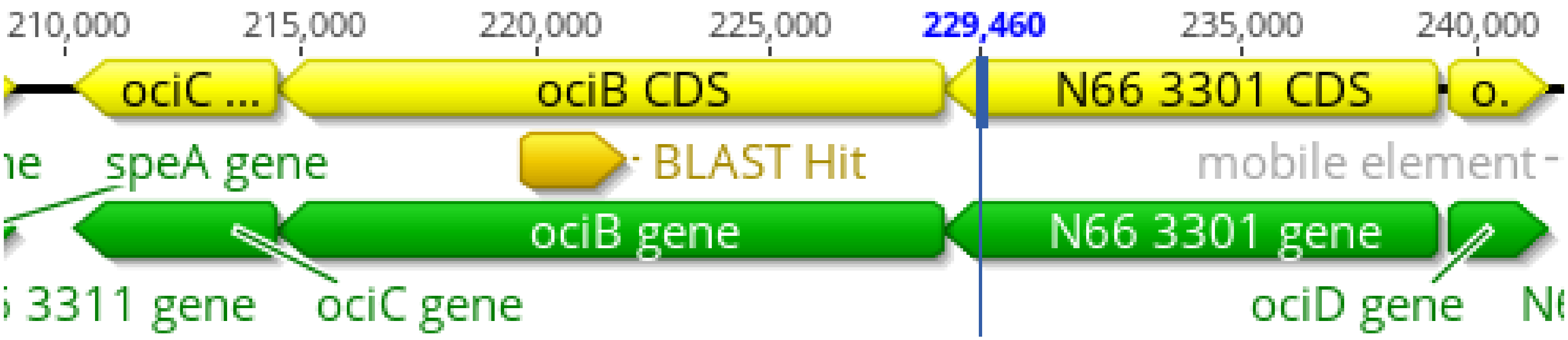

Figure S37. Blast hit to the *ociB* gene (cyanopeptolin biosynthetic pathway) in the metagenomic sequence data from Lake Erie (Showse Park).

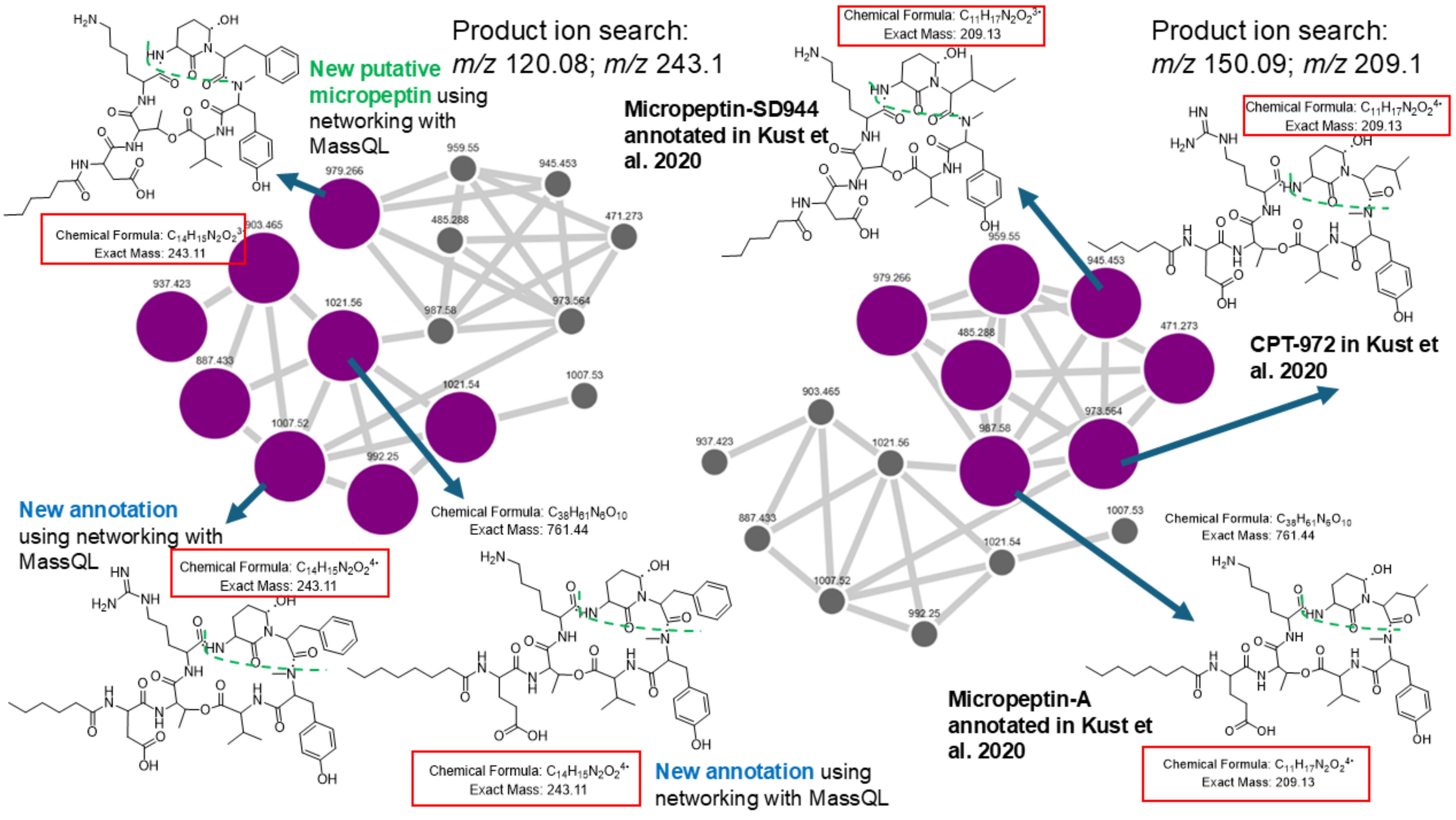

Figure S38. Mass QL annotations of original networks from Kust et al. 2020. Data were publicly available at MassIVE: MSV000085840. The networks were remade according to the previously published methods and the original annotations of CPT-972, micropeptin-A and micropeptin-SD944 were noted. Product ion searches differentiated leucine/isoleucine containing molecules (*m/z* 209.1) from those with phenylalanine (*m/z* 243.1) and new annotations were made for the previously characterized molecules cyanopeptolin 1020B (*m/z* 1021) and cyanopeptolin 1006 (*m/z* 1007). We could additionally propose a new molecule in the annotated network as cyanopeptolin 978B (*m/z* 979).

REFERENCES

1) Fujii, K.; Sivonen, K.; Kashiwagi, T.; Hirayama, K.; Harada, K. -I. Nostophycin, a Novel Cyclic Peptide from the Toxic Cyanobacterium *Nostoc* sp. 152. *J. Org. Chem*. **1999**, *64* (16), 5777–5782.
2) Kust, A.; Řeháková, K.; Vrba, J.; Maicher, V.; Mareš, J.; Hrouzek, P.; Chiriac, M.-C.; Benedová, Z.; Tesařová, B.; Saurav, K. Insight into Unprecedented Diversity of Cyanopeptides in Eutrophic Ponds Using an MS/MS Networking Approach. *Toxins* **2020**, *12* (9), 561.
